# Harnessing Dynamic Heteroleptic Complexation for Self-Assembly of Robust Nested Metallo-Supramolecular Cages

**DOI:** 10.1021/jacs.5c10891

**Published:** 2025-09-09

**Authors:** Soumyakanta Prusty, Hung-Kai Hsu, Mahesh Madasu, Alisha Rani, Jun-Hao Fu, Lin-Ting Lin, Ming-Hao Lee, Ming-Wen Chu, Chun-Hong Kuo, Yi-Tsu Chan

**Affiliations:** † Department of Chemistry, 34881 National Taiwan University, Taipei 106319, Taiwan; ◧ Center for Condensed Matter Sciences and Center of Atomic Initiative for New Materials, National Taiwan University, Taipei 106319, Taiwan; § Department of Applied Chemistry, 34914 National Yang Ming Chiao Tung University, Hsinchu 30010, Taiwan

## Abstract

The exclusive formation of artificial multicomponent assemblies remains a significant challenge, in contrast to the well-established organization observed in natural systems, due to intrinsic entropic constraints. To overcome this limitation, recent efforts have been focused on developing precision self-assembly strategies for the rational construction of such architectures. Here, we construct an ideal complementary pair of 2,2′:6′,2″-terpyridine (tpy)-based ligands by fine-tuning the substituent bulkiness, which enables the quantitative formation of robust nested cages through efficient dynamic heteroleptic complexation with multivalent coordination. The multivalent ligand design proves essential for successful self-assembly, as the smaller incarcerated cage cannot be independently synthesized in an exclusive manner. Notably, the improved solubility and exceptional stability of the nested cage even at low concentrations allow for structural characterization by high-field nuclear magnetic resonance (NMR) spectroscopy, solution-based small-angle X-ray scattering (SAXS), and scanning transmission electron microscopy (STEM). Moreover, its well-defined internal cavity permits the in situ reductive formation of gold nanoparticles, demonstrating its potential as a functional nanoreactor.

## Graphical abstract

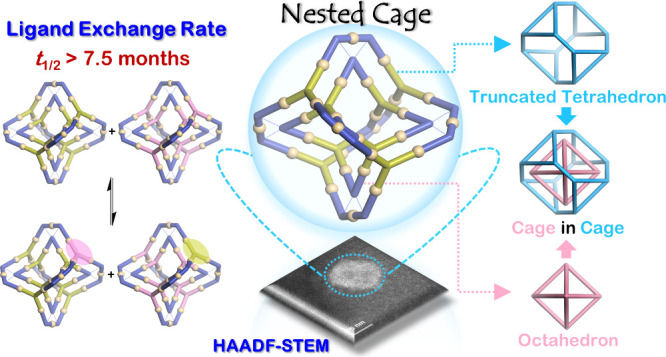

## Introduction

Nested architectures are a fundamental feature of many natural systems, where precise spatial organization enables distinct biological and mechanical functions. For instance, viral capsids^1^ efficiently encapsulate genetic materials, multilamellar lipid vesicles^2,3^ provide compartmentalization for cellular processes, and biomineralized skeletons^4,5^ exhibit hierarchical strength and resilience. These examples illustrate the critical interplay between structure and function, achieved through precise molecular self-assembly. Inspired by this natural sophistication, chemists have sought to mimic such hierarchical architectures in artificial systems, aiming to create functional nanomaterials for catalysis,^6,7^ drug delivery,^8–10^ molecular sensing,^11,12^ etc. Among these artificial systems, nested cage structures generated from pillared architecture design,^13–18^ entwined ligand coordination,^19,20^ π-π interactions,^21,22^ metal nanoclusters,^23^ and metal–organic frameworks (MOFs)^24,25^ are particularly intriguing, characterized by multiple internal cavities within double-shell scaffolds. These structures echo the complexity of natural systems, where nested compartments optimize spatial efficiency and functional diversity. Nevertheless, the rational design and controlled synthesis of such multicomponent architectures remain formidable challenges,^26^ often hindered by undesired competing reactions and the intrinsic difficulty of achieving precise spatial control at the molecular level.

To address these challenges, metal–ligand coordination-driven self-assembly has emerged as a powerful approach for constructing complex supramolecular systems.^27–30^ Among various strategies, dynamic heteroleptic complexation^31,32^ offers high structural precision and modularity, making it particularly suited for multicomponent assemblies. For instance, phenanthroline-based heteroleptic complexes have been utilized in preparation of mechanically interlocked molecules^33,34^ and molecular machines.^35^ Our group has previously demonstrated that 2,2′:6′,2″-terpyridine (tpy)-based complementary ligand pairs can be utilized to construct heteroleptic polygons,^36,37^ polyhedrons,^38^ and copolymers^39^ in one-pot reactions. This approach, when combined with multivalent ligand design,^40^ enables the quantitative formation of ring-in-ring structures^36^ and multicompartment cages,^18^ mimicking the selective and cooperative binding observed in biological systems.^41^ However, as the number of building blocks increases, nonlabile homoleptic complexation^42^ can significantly impede the formation of more intricate architectures, highlighting the need for more precise and delicate ligand design.

In this study, we present a second-generation tpy-based complementary ligand pair, specifically engineered to fully suppress irreversible homoleptic complexation through the strategic incorporation of bulky 6,6″-terpyridyl substituents. This design drives the exclusive formation of heteroleptic complexes with Cd^II^ ions under ambient conditions and, in combination with multivalent coordination, enables the self-assembly of a nested molecular cage featuring an inner octahedron encapsulated by an outer truncated tetrahedron. The resulting bilayered architecture, bridged by 12 octamethylenedioxy chains, exhibits excellent solubility and remarkable stability even at low concentrations, facilitating detailed structural characterization by high-field nuclear magnetic resonance (NMR) spectroscopy, small-angle X-ray scattering (SAXS), and scanning transmission electron microscopy (STEM). Moreover, the well-defined internal cavity provides a confined environment for the in situ reductive formation of gold nanoparticles, implying its potential as a functional nanoreactor.

## Results and Discussion

### Investigation of Substituent Effects on Complementary Ligand Pairing

Our previous studies^31,36–38,43,44^ have demonstrated that tpy-based complementary ligand pairing can be applied to the quantitative preparation of various well-defined heteroleptic metallo-supramolecules. In stoichiometric complexation with metal ions, the formation of heteroleptic complexes is driven by additional ion-dipole interactions and π-stacking stabilization, primarily due to the presence of 2,6-dimethoxyphenyl substituents.^36^ However, when 6,6″-di­(2,6-dimethoxyphenyl)-substituted tpy ligands, such as **L** ^**a**^ (Figure 1a), are used at a ligand-to-metal molar ratio of 2:1, homoleptic complexes can be still formed (Figures S21–S23). To completely eliminate such undesired nonlabile homoleptic complexes^42^ in a multicomponent self-assembly system, the influence of the 6,6″-terpyridyl substituent on the dynamic heteroleptic complexation was carefully investigated. It was assumed that a bulkier group than 2,6-dimethoxyphenyl could prevent homodimer formation entirely. To verify this assumption, the 2,6-diisopropoxyphenyl group was chosen as a substituent for the following reasons: (1) the increased steric hindrance would further decelerate homodimer formation; (2) metal centers could still engage in ancillary ion-dipole interactions with the isopropoxy units; and (3) the bulkier group would enhance the solubility of the resultant complex. Hence, ligand **L** ^**b**^ (Figure 1a) was synthesized via the Suzuki-Miyaura coupling reaction between 2,6-diisopropoxyphenylboronic acid and 2-acetyl-6-bromopyridine, followed by a modified Kröhnke reaction with *p*-anisaldehyde (Scheme S1). As expected, upon treatment of **L** ^**b**^ with 0.5 equiv of Cd^II^ ions, no homoleptic complex [Cd**L** ^**b**^_2_] was formed. Instead, the reaction yielded [Cd**L** ^**b**^] along with uncoordinated **L** ^**b**^, even after heating at 80 °C for 3 days (Figures S27 and S28).

**Figure 1:**
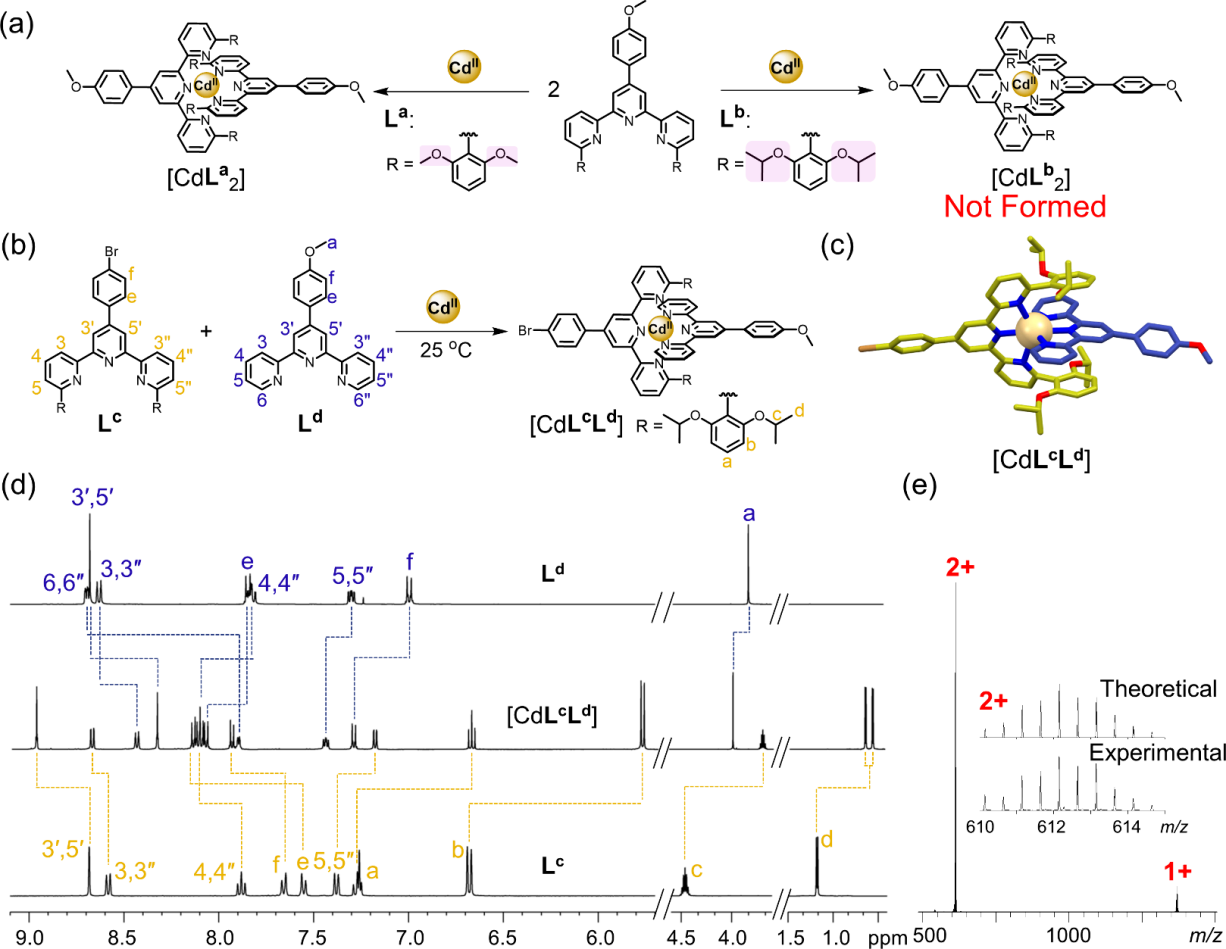
Chemical modification of complementary ligand pairs toward ideal heteroleptic complexation. (a) Schematic representation of distinct homoleptic complexation behaviors for ligands **L** ^**a**^ and **L** ^**b**^ in the presence of Cd^II^ ions. (b) Spontaneous formation of the heteroleptic complex [Cd**L** ^**c**^ **L** ^**d**^] under ambient conditions. (c) X-ray crystal structure of [Cd**L** ^**c**^ **L** ^**d**^]. Hydrogen atoms, PF_6_^–^ ions, and solvent molecules are omitted for clarity. (d) ^1^H NMR spectra of **L** ^**c**^, [Cd**L** ^**c**^ **L** ^**d**^], and **L** ^**d**^. (e) ESI-MS spectrum of [Cd**L** ^**c**^ **L** ^**d**^] and isotope patterns of [M – 2PF_6_]^2+^.

For testing the heteroleptic complexation, the bromo-substituted ligand **L** ^**c**^ was chosen over the methoxy-substituted ligand **L** ^**b**^ to avoid potential overlapping of the ^1^H NMR signals between the methoxy units of **L** ^**b**^ and **L** ^**d**^. Ligand **L** ^**c**^ was synthesized using a procedure similar to that of **L** ^**b**^, while ligand **L** ^**d**^ was prepared following a literature protocol.^45^ A mixture of **L** ^**c**^, **L** ^**d**^, and Cd^II^ in a 1:1:1 ratio resulted in the quantitative formation of the heteroleptic complex [Cd**L** ^**c**^ **L** ^**d**^] under ambient conditions (Figure 1b). The ^1^H NMR spectrum of the complex displayed three sharp singlets at δ = 8.96 (3′,5′-tpy*H*s of **L** ^**c**^), 8.33 (3′,5′-tpy*H*s of **L** ^**d**^), and 3.99 (OC*H*_3_ of **L** ^**d**^) ppm, along with a septet at δ = 3.70 (−C*H*- of isopropoxy group of **L** ^**c**^) ppm, which are indicative of heteroleptic complex formation (Figure 1d). The presence of two doublets assigned to the −C*H*_3_ groups of the isopropoxy moieties was attributed to the restricted rotation of the C–O bonds after heteroleptic complexation, creating two distinct chemical environments. Complete assignments of the ^1^H NMR spectrum for [Cd**L** ^**c**^ **L** ^**d**^] were made with the assistance of COSY and ROESY spectral data (Figures S31–S34). Formation of [Cd**L** ^**c**^ **L** ^**d**^] was further confirmed by electrospray ionization mass spectrometry (ESI-MS), which revealed two peaks at *m*/*z* = 1369.4740 and 612.1437 corresponding to [M – PF_6_]^+^ and [M – 2PF_6_]^2+^, respectively (Figure 1e). The structure of [Cd**L** ^**c**^ **L** ^**d**^] was conclusively validated by single-crystal X-ray diffraction analysis. Single crystals suitable for X-ray data collection were obtained via vapor diffusion of diethyl ether into an MeCN solution of the complex. The complex crystallized in a monoclinic system with the space group *P2*_*1*_ */c*. X-ray crystallographic analysis (Figure 1c) revealed that the Cd^II^ center is situated in a pseudo-octahedral geometry. π-Stacking interactions between the two 2,6-diisopropoxyphenyl rings and the central pyridine unit of **L** ^**d**^ provide extra stability for the heteroleptic structure compared to homoleptic [Cd**L** ^**d**^_2_]. Moreover, the formation constant (1.44 ± 0.27 × 10^15^ M^–2^) for [Cd**L** ^**b**^ **L** ^**d**^]­(NTf_2_)_2_ in MeCN was determined by isothermal titration calorimetry (Figure S37), demonstrating that the heteroleptic complex possesses significant thermodynamic stability.

### Self-Assembly of Heteroleptic Cages

The stepwise construction of a metallo-octahedron has been demonstrated using *C*_3_-symmetrical *tris*-tpy and 60°-bent V-shaped *bis*-tpy ligands in combination with Ru^II^ and Cd^II^ ions.^46^ To develop a more efficient synthetic approach, the one-pot self-assembly of such an octahedral cage utilizing the aforementioned complementary ligand pair was explored. For this purpose, *tris*-tpy **F** ^**1**^ and *bis*-tpy **L** ^**e**^ were designed and synthesized (Figure 2a and Scheme S1). A mixture of **F** ^**1**^, **L** ^**e**^, and Cd­(NO_3_)_2_·4H_2_O in a stoichiometric ratio of 2:3:6 was stirred at 25 °C for 30 min, after which excess NH_4_PF_6_ was added to convert the counteranions from NO_3_^–^ to PF_6_^–^. The counterion-exchanged product was redissolved in MeCN, and the solution was stirred at 80 °C for 24 h. ESI-MS analysis of the final complex suggested the formation of the target cage [Cd_12_ **F** ^**1**^_4_ **L** ^**e**^_6_], along with other assemblies, including [Cd_18_ **F** ^**1**^_6_ **L** ^**e**^_9_] and [Cd_6_ **F** ^**1**^_2_ **L** ^**e**^_3_]. ^1^H and DOSY NMR spectra (Figures S38, S42, and S43), recorded at different concentrations, revealed a concentration-dependent assembly behavior.^47,48^ At higher concentrations, the larger assembly [Cd_18_ **F** ^**1**^_6_ **L** ^**e**^_9_] was generated, whereas at lower concentrations, the smaller assembly, [Cd_6_ **F** ^**1**^_2_ **L** ^**e**^_3_] was observed. In both cases, [Cd_12_ **F** ^**1**^_4_ **L** ^**e**^_6_] was also present. The ^1^H NMR signals corresponding to [Cd_6n_ **F** ^**1**^_2n_ **L** ^**e**^_3n_] (n = 1–3) were carefully assigned based on 2D COSY and ROESY experiments (Figures S39–S41). Furthermore, ESI-MS analysis performed at varying concentrations (Figure S44) corroborated the NMR observations. Despite the inherent structural flexibility of **L** ^**e**^, the failure to exclusively form the expected octahedron likely arose from its bend angle deviating from the ideal dihedral angle of 70.5° at a vertex.

**Figure 2:**
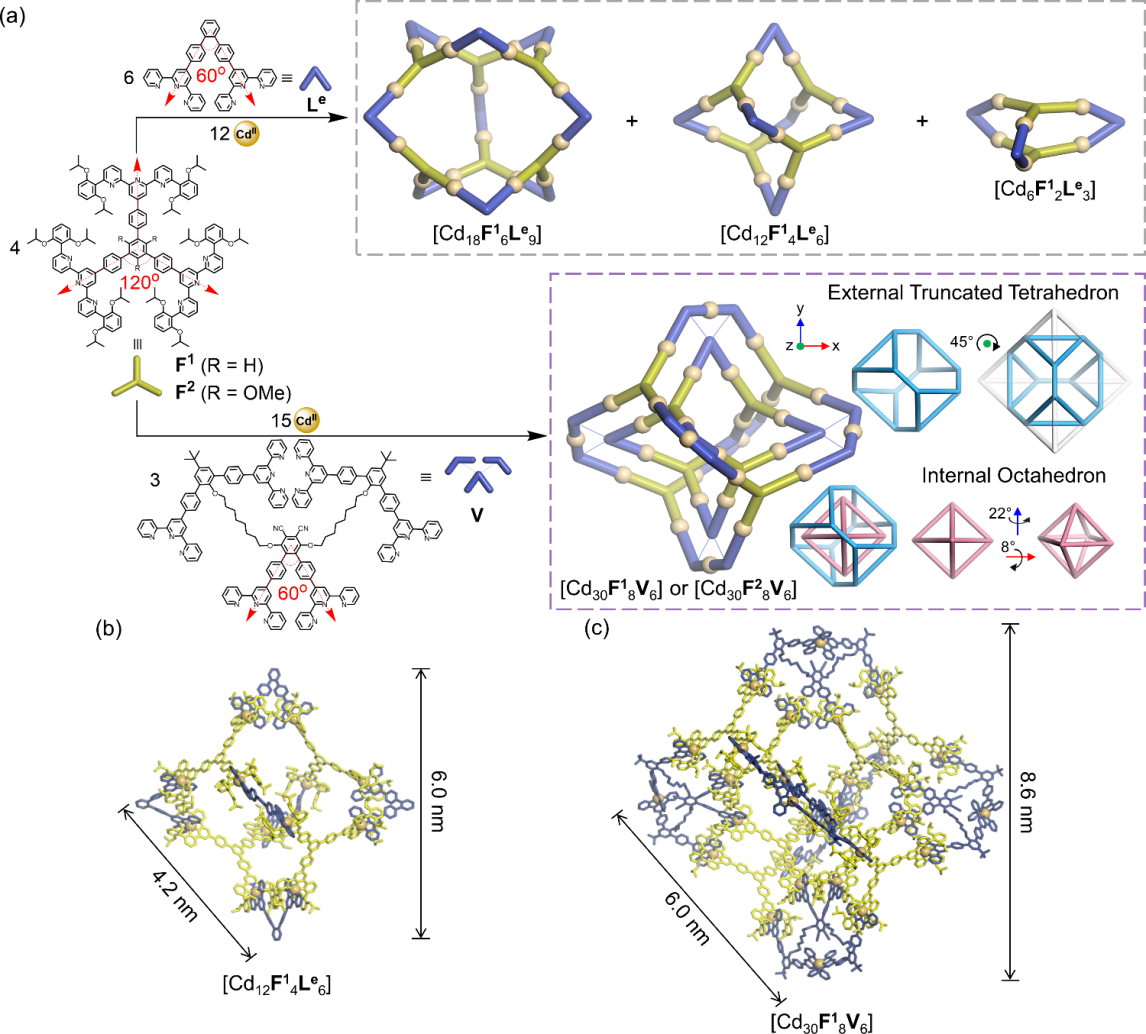
Self-assembly and molecular models of the metallo-supramolecular cages. (a) Ligand combinations for self-assembly of [Cd_6n_ **F** ^**1**^_2n_ **L** ^**e**^_3n_] (*n* = 1–3), [Cd_30_ **F** ^**1**^_8_ **V**_6_], and [Cd_30_ **F^2^**_8_ **V**_6_]. Geometry-optimized structures and dimensions of (b) [Cd_12_ **F** ^**1**^_4_ **L** ^**e**^_6_] and (c) [Cd_30_ **F** ^**1**^_8_ **V**_6_].

### Self-Assembly of Nested Cages

Multivalency and cooperativity^49^ are recognized as critical factors in both natural and artificial self-assembly processes, facilitating the construction of complex yet well-defined structures. Based on this principle, it was hypothesized that the exclusive formation of a three-dimensional cage could be achievable through the rational design of multivalent ligands. To test this hypothesis, the multivalent ligand **V** was selected as a replacement for the *bis*-tpy ligand (**L** ^**e**^). Ligand **V**, consisting of two 120°-bent *bis*-tpy units linked to a central 60°-bent *bis*-tpy through octamethylenedioxy spacers (Figure 2a), was synthesized following a previously reported procedure.^40^ Notably, **V** is capable of undergoing intramolecular homoleptic complexation with Cd^II^ ions, leading to the formation of a tetratopic metalloligand with two pairs of parallel coordinative tpy sites. This specific structural arrangement serves as the key design element for the creation of a nested metallo-supramolecular cage, which comprises an external truncated tetrahedron encapsulating an internal octahedron (Figure 2a). Accordingly, a stoichiometric mixture of **F** ^**1**^, **V**, and Cd­(NO_3_)_2_·4H_2_O in a molar ratio of 4:3:15 was stirred at ambient temperature for 30 min in CHCl_3_/MeOH (1/1, v/v). Excess NH_4_PF_6_ was then added to exchange the counteranions. After heating the resultant complex in CD_3_CN at 80 °C for 2 days, the ^1^H NMR spectrum (800 MHz) revealed sharp and well-resovled peaks with five distinct sets of terpyridyl signals, providing preliminary evidence for the formation of the target structure, [Cd_30_ **F** ^**1**^_8_ **V**_6_] (Figure 3a). It is noteworthy that under identical reaction conditions, the 6,6″-di­(2,6-dimethoxyphenyl)-substituted *tris*-tpy ligand yielded insoluble precipitates, suggesting that the isopropoxy groups in **F** ^**1**^ significantly enhanced solubility during the self-assembly process. The DOSY NMR experiment (Figure 3b) confirmed the prencence of a single species with a diffusion coefficient of 1.50 × 10^–10^ m^2^ s^–1^ in CD_3_CN. All the ^1^H NMR signals from the complex were carefully assigned using COSY and ROESY experiments (Figures S46–S55). The complex was found to be stable across a range of concentrations (1–32 mg mL^–1^ in CD_3_CN), as observed by ^1^H NMR (Figure S56). Furthermore, the ^113^Cd NMR spectrum of the complex (Figure 3c) showed three sharp peaks, which were in accord with the molecular symmetry of [Cd_30_ **F** ^**1**^_8_ **V**_6_]. When compared to [Cd**L** ^**d**^_2_] and [Cd**L** ^**c**^ **L** ^**d**^] (Figure S36), the resonance at δ = 266.97 ppm was assigned to the homoleptic Cd^II^ center, while the signals at δ = 239.38 and 236.96 ppm were attributed to the heteroleptic Cd^II^ nuclei.

**Figure 3:**
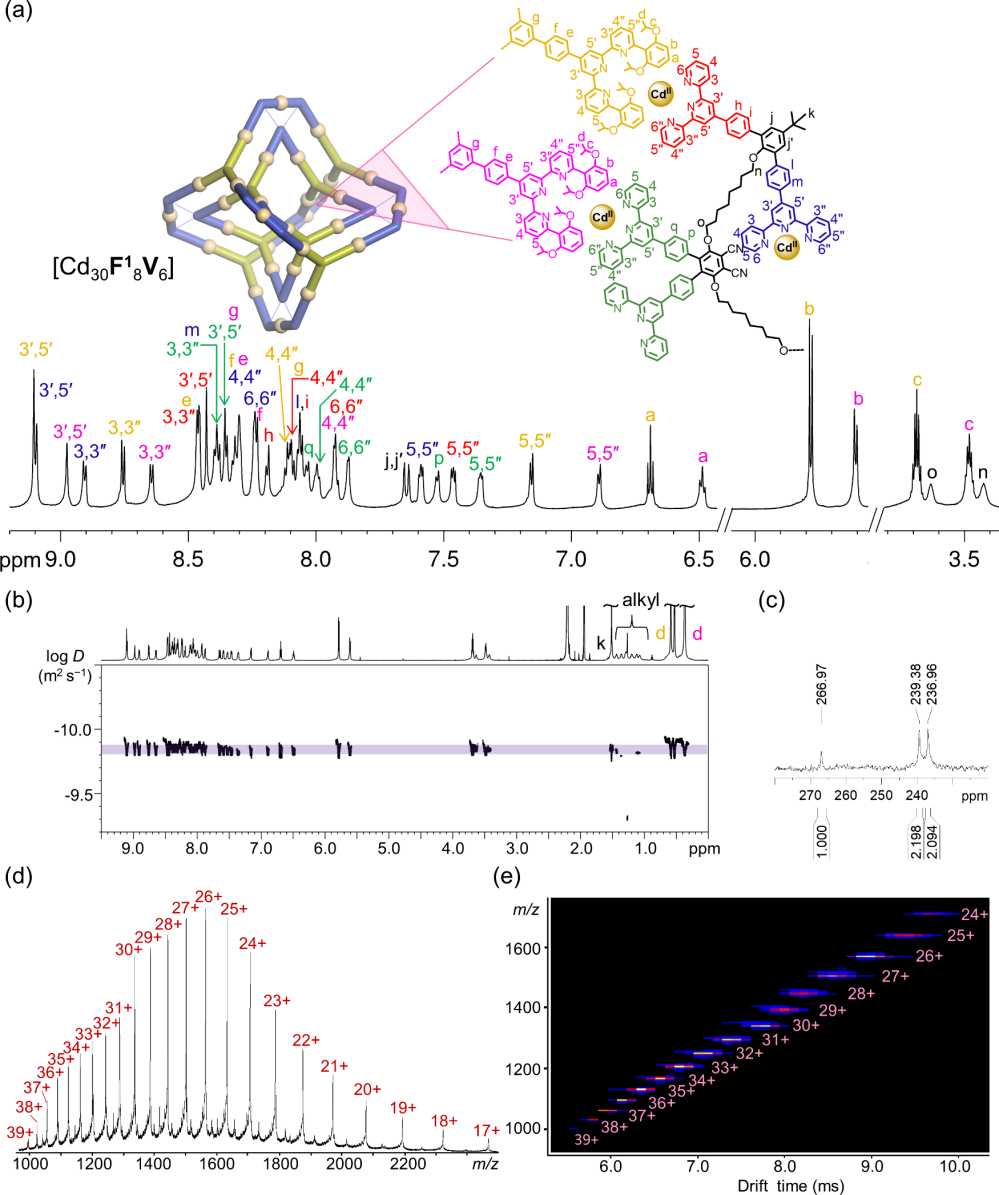
Structural characterization of [Cd_30_ **F** ^**1**^_8_ **V**_6_]. (a) ^1^H (800 MHz), (b) DOSY (500 MHz), and (c) ^113^Cd (500 MHz) NMR spectra in CD_3_CN at 25 °C. (d) ESI-MS spectrum and (e) TWIM-MS plot.

Finally, ESI-MS analysis confirmed the chemical composition of [Cd_30_ **F** ^**1**^_8_ **V**_6_], which has a molecular weight of 44,451 *Da*, revealing major peaks corresponding to gas-phase ions with charge states ranging from 17+ to 39+ (Figure 3d). To gain further structural insights into the nested architecture, electrospray ionization coupled with traveling wave ion-mobility mass spectrometry (ESI-TWIM-MS)^50–53^ was employed. The average experimental collision cross-section (4214.3 ± 110.6 Å^2^), deduced from the drift times, closely matched the theoretical value (4192.2 ± 144.9 Å^2^) obtained from annealing simulation and trajectory calculations^54^ (Table S2). The absence of other isomers was supported by the narrow drift time distributions observed for the 24+ to 39+ species in the ESI-TWIM-MS plot (Figure 3e).

### Experiments to Evaluate Molecular Stability

To assess kinetic stability of the ligand exchange process between cages, the isostructural cage [Cd_30_ **F** ^**2**^_8_ **V**_6_] (Figure 2a) was constructed using the tritopic ligand **F** ^**2**^, which contains three additional methoxy groups compared to **F** ^**1**^. This modification allowed for a clear distinction in molecular weight between [Cd_30_ **F** ^**2**^_8_ **V**_6_] and [Cd_30_ **F** ^**1**^_8_ **V**_6_], enabling accurate monitoring of the ligand exchange process. The newly constructed cage was thoroughly characterized using NMR and ESI-MS (Figures S58–S63) to confirm its structure and composition. The difference in molecular weight between the two cages was utilized to measure the ligand exchange rate via ESI-MS analysis^55^ (Figures S64–S66). Experimental results indicated that the half-life for the ligand exchange process between two cages was a markedly prolonged half-life, estimated to exceed 7.5 months at 25 °C. This exceptional stability contrasts sharply with the substantially shorter half-life of 34 s observed for the mononuclear heteroleptic complex [Cd**L** ^**c**^ **L** ^**d**^], as determined by ^1^H exchange spectroscopy (EXSY) NMR experiments (Figure S67). The dramatic difference in half-lives illustrates the enhanced kinetic stability of the multicomponent supramolecular cages, which is presumably attributed to the intricate multivalent interactions within the cage architecture.

### Structure Characterization by Microscopy and X-ray Scattering

Various microscopy techniques were employed to visualize these well-defined nanoobjects and gain deeper structural insights. Atomic force microscopy (AFM) images of the cage (Figure S68) displayed two major average heights of 8.5 ± 0.4 nm and 5.9 ± 0.4 nm, which could be ascribed to different orientations of the molecules on the surface, as suggested by the molecular modeling (Figure 2c). Cryogenic electron microscopy (cryo-EM) analysis of [Cd_30_ **F** ^**1**^_8_ **V**_6_] was conducted in MeCN/H_2_O (1/4, v/v) at a concentration of 3 × 10^–6^ M. The corresponding cryo-EM micrograph (Figure 4a) exhibited chain-like aggregates composed of individual molecules with an average size of 7.5 ± 0.8 nm. This aggregation was presumably induced by the ionic nature of metal complexes. Notably, when the solvent composition was adjusted to a more MeCN-rich mixture (MeCN/H_2_O = 3/1, v/v), dissociated single molecules were observed (Figure 4b) possibly because of improved solubility in MeCN. To further visualize single molecules, scanning transmission electron microscopy (STEM) was utilized. High-resolution high-angle annular dark-field (HAADF)-STEM images (Figures 4c–e and S69) clearly revealed both the dimeric aggreagtion and the single-molecule framework of [Cd_30_ **F** ^**1**^_8_ **V**_6_], closely resembling the corresponding geometry-optimized CPK models (Figure 4f). Although soft materials are typically vulnerable to high-voltage electron beams,^56^ the significant robustness of [Cd_30_ **F** ^**1**^_8_ **V**_6_] allowed for the acquisition of single-molecule images even under such harsh conditions.

**Figure 4:**
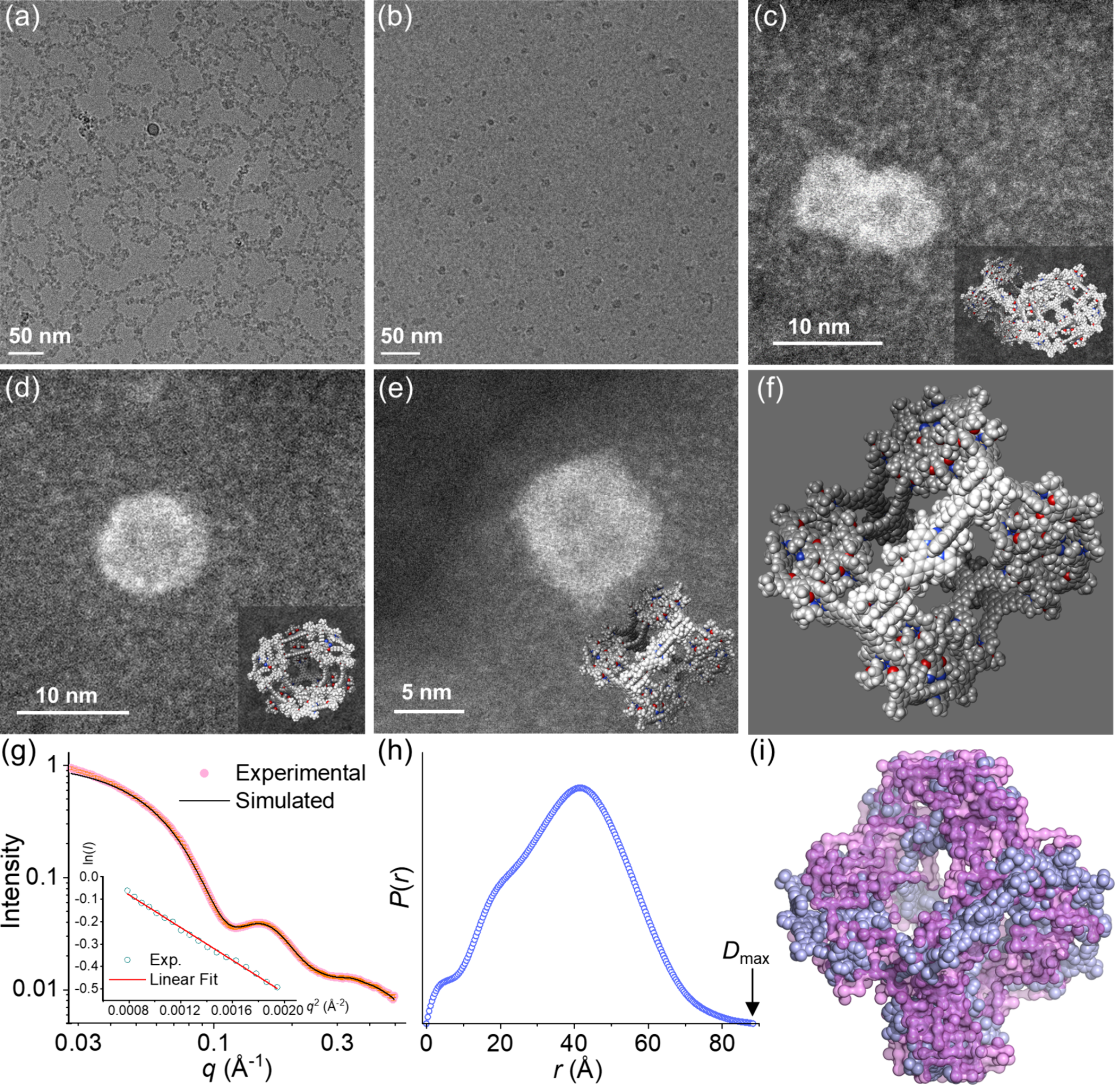
Electron microscopy and SAXS analysis of [Cd_30_ **F** ^**1**^_8_ **V**_6_]. (a, b) Cryo-EM images. (c–e) HAADF-STEM images with the corresponding possible geometry-optimized CPK models shown at the bottom right of each panel. Image c depicts the aggregation of two nested cages. (f) Geometry-optimized CPK model of [Cd_30_ **F** ^**1**^_8_ **V**_6_] showing structural resemblance to the features observed in image e. (g) SAXS profiles derived from the experiments (pink dot) and the bead model (black line). The error bars represent the uncertainties in the scattering intensity. The inset is the Guinier plot obtained from the experimental data (green circle) at *qR*_g_ < 1.3 and the fitted linear regression curve (red line). (h) Pair distance distribution function generated from the experimental SAXS data. (i) Superposed image of the simulated bead model (pink) and the geometry-optimized structure (light blue). The overlapped part is shown in purple.

The morphological features of [Cd_30_ **F** ^**1**^_8_ **V**_6_] in MeCN was further examined under dilute conditions using small-angle X-ray scattering (SAXS). The nanoscopic shape and size of the cage were evaluated by fitting the experimental SAXS profile to a specific topological model. The linearity of the Guinier plot in the range of *qR*_g_ < 1.3 consistently indicated the presence of a monodisperse solution of [Cd_30_ **F** ^**1**^_8_ **V**_6_] (Figure 4g). The corresponding radius of gyration (*R*_g_), derived from the Guinier approximation, was estimated to be 29.26 ± 0.14 Å, which was in good agreement with the simulated value of 29.41 ± 0.53 Å deduced from the energy-minimized structures after annealing. In addition, the experimental pair distance distribution function (PDDF), *P*(*r*) (Figure 4h), obtained through indirect Fourier transformation of the SAXS data, revealed the maximum particle distance (*D*_max_) of 8.8 nm, which closely aligned with the theoretical dimensions of [Cd_30_ **F** ^**1**^_8_ **V**_6_]. The primary peak and shoulder features observed in the *P*(*r*) curve were consistent with the spatial arrangement of the homoleptic metal center relative to the metal nodes in the inner and outer cages, as illustrated in the 2D-unfolded structural diagram (Figure S74). The structural consistency between experimental and theoretical analyses was further confirmed by superposing the bead model^57^ derived from the SAXS profile with the geometry-optimized structure (Figure 4i).

### Synthesis of Cage-Encapsulated Gold Nanoparticles (Au@Cage)

The void spaces of the nested cage were analyzed using MoloVol,^58^ and the computaional results indicated the presence of two distinct cavities with the respective volumes of 3,280 ± 94 and 14,847 Å^3^. The central cavity, formed by the internal octahedron, is surrounded by four smaller void spaces, which originate from the bilayered compartments (Figures 5h and S70 and Video S1). Building on the unique cavities and robustness of the cage framework, it was hypothesized that the in situ reduction of tetrachloroaurate ions (AuCl_4_^–^) within the cationic scaffold might lead to the formation of cage-encapsulated gold nanoparticles (Au@Cage).^59^ To verify this hypothesis, a mixture of HAuCl_4_·3H_2_O and [Cd_30_ **F** ^**1**^_8_ **V**_6_] in MeCN was treated with NaBH_4_, resulting in a color change to red without producing any precipitates, indicating the reduction of gold­(III) to gold(0). TEM images (Figure 5a,b) confirmed the formation of Au nanoparticles with an average diameter of 2.8 ± 0.4 nm (Figure 5d), which were randomly embedded in the cage matrix, exhibiting a darker contrast than the grid carbon film, as further evidenced by dark-field imaging (Figure 5e). The observed Au nanoparticle size, approximately 12,000 Å^3^, closely matched the calculated central cavity volume, strongly supporting that nanoparticle growth was confined within the cage. The high-resolution TEM image (Figure 5c) revealed a lattice interlayer distance of 0.24 nm, corresponding to the *d*-spacing of the (111) plane of Au. Furthermore, the X-ray photoelectron spectroscopy (XPS) spectrum (Figure S71) displayed Au 4f_7/2_ and Au 4f_5/2_ peaks with binding energies at 82.8 and 86.6 eV, respectively, confirming the presence of reduced gold(0).^60^ Additionally, energy-dispersive X-ray spectroscopy (EDS) analysis revealed the spatial distributions of Au and Cd (Figure 5f), as well as C, N, and O (Figure 5g), in the obtained Au@Cage, again suggesting that the Au nanoparticles were encapsulated in the cage framework.

**Figure 5:**
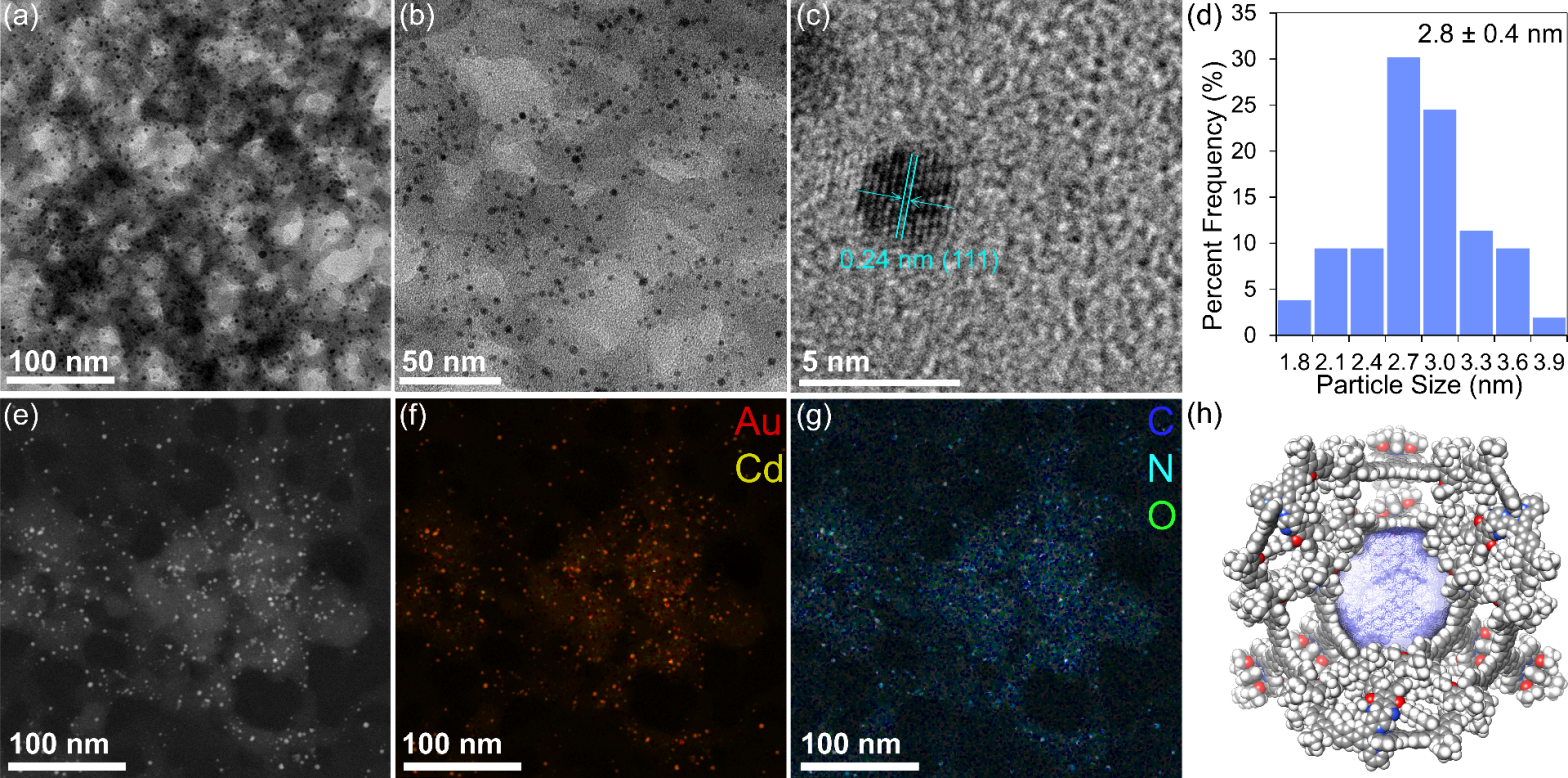
TEM and EDS analyses of Au@Cage. (a-c) TEM images and (d) particle size distribution histogram. (e) Dark-field TEM image and (f, g) EDS elemental maps overlaid on image e, illustrating the spatial distributions of Au and Cd in image f, and C, N, and O in image g, respectively. (d) Computed central cavity of the cage, shown in purple, as generated using MoloVol.

To further investigate the role of cage cavity in the synthesis of Au nanoparticles, the flat metallomacrocycle (FM) [Cd_9_ **V**_3_][Bibr ref40] was utilized as a control. Accordingly, Au@FM and pure Au nanoparticles were synthesized using [Cd_9_ **V**_3_] and in the absence of metal complexes, respectively, following the same protocol used for the preparation of Au@Cage. The TEM images of Au@FM and Au nanoparticles revealed significantly larger average sizes of 7.7 ± 2.2 and 13.6 ± 2.7 nm, respectively, with broader size distributions (Figures S72 and S73). These observations suggest that the confined space and cationic framework of the cage played a crucial role in facilitating the formation of uniform cage-encapsulated Au nanoparticles.

## Conclusions

In summary, the nested metallo-supramolecular cages were successfully self-assembled through the deliberate combination of efficient dynamic heteroleptic complexation and rational multivalent ligand design. Undesired homoleptic complexation pathways, often detrimental to the selectivity and fidelity of self-assembly, were effectively suppressed by strategic control over ligand substituent bulkiness. This molecular-level control also improved solubility, thereby maintaining solution-phase homogeneity during the self-assembly process–a critical factor for ensuring structural uniformity. Kinetic studies revealed remarkable solution stability of the nested cages, with a ligand exchange half-life of >7.5 months. These characteristics enabled comprehensive structural elucidation via high-field NMR, solution-based SAXS, cryo-EM, and HAADF-STEM single-molecule imaging. Furthermore, the well-defined internal cavity of the multicompartment architecture was employed as a confined nanoreactor for the in situ reductive formation of uniform cage-encapsulated gold nanoparticles, whose electrochemical catalytic properties are currently under investigation. This study demonstrated a versatile and generalizable approach for constructing hierarchically organized multicomponent metallo-supramolecular architectures, offering a powerful platform for the bottom-up design of functional materials with programmable structures, enhanced stability, and potential applications in nanoreactors, catalysis, and molecular devices.

## Supplementary Material





## Notes

Video S1: energy-minimized model and MoloVol cavity size analysis of [Cd_30_ **F^1^**_8_ **V**_6_] (MP4)
Detailed experimental procedures, synthesis, ligand exchange studies, characterization data (NMR, MS, ITC, SAXS, AFM, STEM), and X-ray crystallographic information (PDF)
