# Supplementary material for: Harnessing Dynamic Heteroleptic Complexation for Self-Assembly of Robust Nested Metallo-Supramolecular Cages
Source: J Am Chem Soc. 2025 Sep 9;147(37):33914–22. doi: 10.1021/jacs.5c10891 (PMC12447513; doi:10.1021/jacs.5c10891)
Supplement: Supplementary file 1 [file ja5c10891_si_001.pdf]

Supporting Information

Harnessing Dynamic Heteroleptic Complexation for Self-Assembly of Robust Nested Metallo-Supramolecular Cages

Soumyakanta Prusty,[1‡] Hung-Kai Hsu,[1‡] Mahesh Madasu,[1] Alisha Rani,[1] Jun-Hao Fu,[1] Lin-Ting Lin,[1] Ming-Hao Lee,[2] Ming-Wen Chu,[2] Chun-Hong Kuo,[3] and Yi-Tsu Chan[1]*

[1]Department of Chemistry, National Taiwan University, Taipei 106319, Taiwan. [2]Center for Condensed Matter Sciences and Center of Atomic Initiative for New Materials, National Taiwan University, Taipei 106319, Taiwan. [3]Department of Applied Chemistry, National Yang Ming Chiao Tung University, Hsinchu 30010, Taiwan. [‡]These authors contributed equally. *e-mail: ytchan@ntu.edu.tw

Table of Contents

Materials and Methods S2

Synthesis of Ligands S5

General Procedure for Complexation S19

Self-Assembly of [Cd_{30}**F**$^{\mathbf{1}}_{8}$**V**$_6$] and [Cd_{30}**F**$^{\mathbf{2}}_{8}$**V**$_6$] S20

Isothermal Titration Calorimetry Experiments S30

DOSY NMR Experiments S33

Ligand Exchange Experiments S46

AFM and HAADF-STEM Images S49

Synthesis of Au@Cage and Control Experiments S51

X-ray Crystallographic Data S53

Experimental and Theoretical Collision Cross-Sections S54

References S55

Materials and General Methods. Unless mentioned, reagents and solvents were purchased from Fisher Scientific, AK Scientific, and Sigma-Aldrich and used without further purification. Column chromatography was conducted using silica gel (45-75 μm) from Fuji Silysia GS series and basic Al_2O_3 (50-200 μm) from Acros. ^1H and ^{13}C NMR spectra were recorded at 25 °C on Bruker DPX-400, Bruker AVIII-400, Bruker AVIII-500, and Bruker AVIII-800 NMR spectrometers, where chemical shifts (δ in *ppm*) were determined with respect to the nondeuterated solvents as a reference. The ^{113}Cd NMR spectra were recorded at 25 °C on a Bruker AVIII-500 NMR spectrometer and were referenced to an external standard of $Cd(ClO_4)_2$ (0 ppm). The 2D COSY, ROESY, and DOSY spectra were recorded at 25 °C on Bruker AVIII-400 and Bruker AVIII-500 NMR spectrometers. The atomic force microscopy (AFM) images were recorded on a Bruker Dimension Icon system in standard tapping mode under ambient conditions. Bruker SCANASYST-AIR probes with a resonance frequency of 70 kHz and a force constant of 0.4 N/m were utilized, and the data were processed by NanoScope Analysis version 1.5 (Bruker Software, Inc.). Samples for AFM were prepared by spin-coating (500 rpm for 1 min) a sample solution (1×10^{-8} M) on a freshly cleaved mica surface. Transmission electron microscopy (TEM) and energy-dispersive X-ray spectroscopy (EDS) analyses were conducted on a JEOL JEM-2100F microscope operating at 200 kV. High-angle annular dark-field (HAADF)-scanning transmission electron microscopy (STEM) experiments was conducted on a spherical-aberration corrected JEOL 2100FX microscope operating at 200 kV. Samples for TEM and STEM measurements were prepared by drop-casting a sample solution (1×10^{-7} M) onto a carbon-coated copper grid and dried *in vacuo* for 24 h. Cryo-EM images were collected on the Gatan UltraScan 4000 CCD detector at a nominal magnification of 62000×, resulting in a calibrated sampling of 1.326 Å per pixel, by using an FEI Tecnai G2 F20 TWIN microscope operating at 200 kV. Samples for cryo-EM measurements were prepared according to the reported method.[1] High-resolution X-ray photoelectron spectra were acquired on a ULVAC-PHI PHI Quantera II spectrometer.

Mass Spectrometry and Ion Mobility. ESI mass spectrometry and traveling wave ion-mobility (TWIM) experiments were conducted on a Waters Synapt HDMS G2 instrument with a LockSpray ESI source using the literature parameters.[2] Matrix-assisted laser desorption/ionization coupled with a time-of-flight detector (MALDI-TOF) mass spectrometry was conducted on a Bruker autoflex™ speed spectrometer with a 355 nm frequency tripled Nd:YAG SmartBeam® laser. 1 μL of 2,5-dihydroxybenzoic acid (DHB) matrix solution (10 mg/mL in MeCN) or 1 μL of α-cyano-

4-hydroxycinnamic acid (CHCA) matrix solution (10 mg/mL in a mixture of MeCN/H_2O/TFA = 50/49.9/0.1 wt%) was deposited on a MALDI plate and air-dried. Aliquots of sample solution (1 mg/mL in CHCl3) were added onto the matrix spots for the measurements acquired in reflection mode.

Determination of MS Charge States. Due to the high charge states and the high molecular weight, the isotope pattern for each charge state could not be resolved even with a high-resolution TOF detector, but the major consecutive peaks agreed well with the simulated *m/z* ratios. To determine the charge states and molecular weights of these multivalent ions in the absence of isotopic resolution, we followed the method described by Covey, which is based on the *m/z* values of adjacent peaks.[3] The charge state (n_2) corresponding to a given ion at $m/z = m_2$, was determined using Eq. (1):

$$n_2 = \frac{m_1 - X}{m_2 - m_1} \qquad (1)$$

where m_1 and m_2 are the *m/z* values of two adjacent peaks (with $m_2 > m_1$), and X is the mass of the charged species lost or gained—in our case, one PF_6^- anion (144.96 Da), so $X = -144.96$. Once the charge state was determined, the molecular weight (M) of the species was calculated using Eq. (2):

$$M = n_2(m_2 - \mathrm{X}) \qquad (2)$$

For example, in the case of [$Cd_{30}$$\mathbf{F^1}_8\mathbf{V}_6$]$(PF_6)_{60}$, two adjacent peaks were observed at m_1 = 1633.0560 and m_2 = 1707.0869. This analysis indicates that the peak at 1707.0869 corresponds to the 24+ charge state, while the adjacent lower *m/z* peak at 1633.0560 corresponds to the 25+ charge state. The same procedure was applied to assign charge states for the other complexes.

Small-Angle X-ray Scattering. For SAXS measurements, all the SAXS profiles were acquired at the TLS 23A beamline of the National Synchrotron Radiation Research Center (NSRRC) in Taiwan. The X-ray with a wavelength of λ = 0.827 Å (15 keV) and a typical current of 360 mA was used. A 2D PILATUS 1M-F detector was used to capture the scattering patterns. The sample-to-detector distance is 2765.30 mm to give a q range of 0.03 to 0.49 Å^{-1}, where q is the scattering vector related to the scattering angle (2θ) and the photon wavelength (λ) by $q = 4\pi\sin(\theta)/\lambda$. The 2D

SAXS images were converted into 1D SAXS profiles $I(q)$ to q followed by background subtraction. SAS Data analysis software was used to analyze all SAXS data and the models were used for fitting the form-factor from the ATSAS package. The experimental scattering data were compared to a simulated scattering profile, created by the program CRYSOL using a geometry-optimized molecular model. Guinier regions and radius of gyration (R_g) estimates were derived from the Guinier approximation,[4] $I(q) \simeq I(0)\exp(-q^2R_g^2/3)$, where $qR_g < 1.3$, using the program PRIMUS. Pair distance distribution function, $P(r)$, obtained by the GNOM program was used to determine the maximum particle diameter (D_{max}) for bead modeling. Computation of the theoretical scattering profile was performed by the CRYSOL program. Bead modeling and fitting of the experimental profile were performed by using the DAMMIN program. All sample solutions and solvents were filtered into empty vials through a 0.22 μm syringe filter before the measurement. 100 μL of the sample solution was added into a quartz capillary (Hampton Research HR6-151) with an outer diameter of 2.5 mm and a wall thickness of 0.01 mm during the measurement. The analytical solutions (8.9 and 0.4 mg mL^{-1}) were prepared at 25 °C. Acetonitrile was used as blank to conduct background subtraction.

Molecular Modeling. Energy-minimized structures were obtained following the settings in the literature,[2, 5] Calculations were proceeded with Geometry Optimization and followed by Anneal in Forcite module of Materials Studio version 7.0 program (Accelrys Software, Inc.). For each structure, 200 conformations were generated after annealing and converted into the corresponding collision cross-sections (CCSs) using projection approximation (PA) and trajectory method (TM) in MOBCAL.[6]

X-ray Crystallography. Single-crystal X-ray data were collected on an Oxford Diffraction Gemini A CCD diffractometer and processed with CrysAlisPro software (Agilent Technologies). Graphite monochromated Cu-Kα radiation (λ = 1.54178 Å) at 200(2) K was used in the diffraction data collection. Empirical absorption correction was done by spherical harmonics in the SCALE3 ABSPACK. The structure was solved and refined by applying SHELXS-97 and SHELXL-2019/3 programs.[7] The structure was deposited at the Cambridge Crystallographic Data Center with CCDC number 2456141.

Synthesis of Ligands. 2-Acetyl-6-bromopyridine (**2**),[8] 4-methoxybenzaldehyde,[9] 1,3-diisopropoxybenzene (**4**),[10] 1,3,5-tribromo-2,4,6-trimethoxybenzene,[11] **L**[e],[12] and **V**[13] were synthesized following the reported procedures.

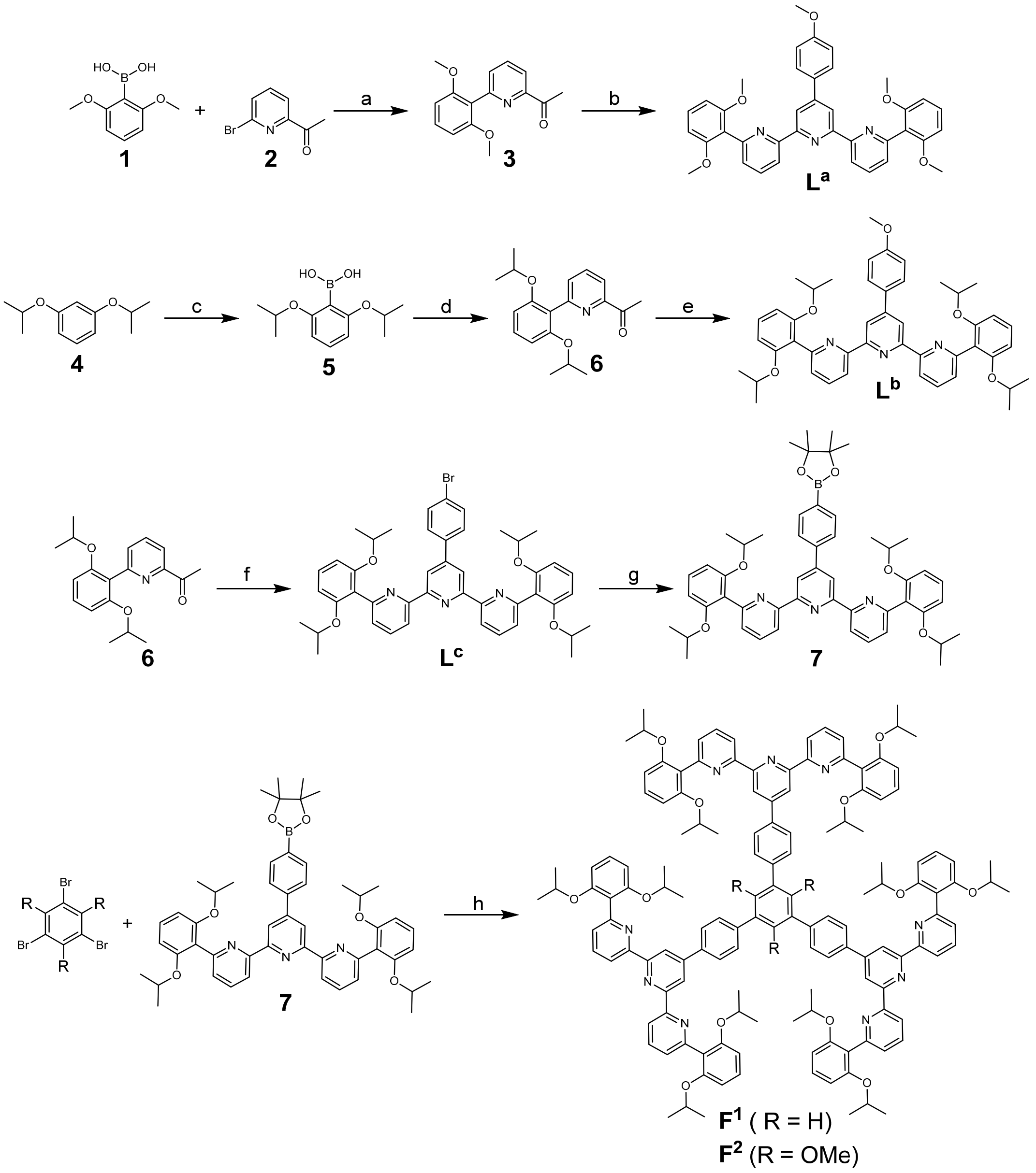

Scheme S1. Synthesis of ligands $\mathbf{L^a}$, $\mathbf{L^b}$, $\mathbf{L^c}$, $\mathbf{F^1}$, and $\mathbf{F^2}$. Reagents and conditions: (a) $Pd(PPh_3)_4$, Na_2CO_3, 1,4-dioxane/H_2O (2:1, v/v), reflux, 24 h; (b) (i) 4-methoxybenzaldehyde, $NaOH_{(aq)}$, EtOH, 24 h, (ii) $NH_4OH_{(aq)}$, reflux, 24 h; (c) (i) *n*-BuLi, THF, -20 to 25 °C, 2 h, (ii) trimethylborate, -65 to 25 °C, 12 h; (d) **2**, $Pd(PPh_3)_4$, Na_2CO_3, *t*-BuOH/PhMe/H_2O (1:3:3, v/v/v), reflux, 24 h; (e) (i) 4-methoxybenzaldehyde, $NaOH_{(aq)}$, EtOH, 24 h, (ii) $NH_4OH_{(aq)}$, reflux, 24 h; (f) (i) 4-bromobenzaldehyde, $NaOH_{(aq)}$, EtOH, 24 h, (ii) $NH_4OH_{(aq)}$, reflux, 24 h; (g) bis(pinacolato)diboron, KOAc, $Pd(dppf)Cl_2$, 1,4-dioxane, reflux, 3 h; (h) $Pd(PPh_3)_4$, Na_2CO_3, *t*-BuOH/PhMe/H_2O (1:3:3, v/v/v), reflux, 72 h.

Synthesis of 3. To a degassed two-neck flask containing **2** (10.0 g, 50.0 mmol), **1** (9.5 g, 50.2 mmol), Na_2CO_3 (15.9 g, 150.0 mmol), and $Pd(PPh_3)_4$ (1.1 g, 1.0 mmol), a mixed solvent (150 mL) of 1,4-Dioxane/H_2O (2:1, v/v) was added after being purged with N_2 for 30 min. The reaction mixture was refluxed for 24 h under N_2. After cooling to 25 °C, 1,4-dioxane was evaporated using rotary evaporator. The mixture was extracted with CH_2Cl_2 and the combined organic extract was dried over $MgSO_4$ and then evaporated to dryness under reduced pressure. The residue was recrystallized from hexane to give **3** (12.5 g, 93%). ^1H NMR (400 MHz, $CDCl_3$): δ (ppm) 7.97 (dd, J = 7.8 and 1.1 Hz, 1H), 7.83 (t, J = 7.8 Hz, 1H), 7.47 (dd, J = 7.8 and 1.1 Hz, 1H), 7.3 (t, J = 8.4 Hz, 1H), 6.69 (d, J = 8.4 Hz, 2H), and 2.7 (s, 3H). ^{13}C NMR (100 MHz, $CDCl_3$): δ (ppm) 200.91, 158.15, 153.89, 153.39, 136.17, 129.94, 129.76, 119.52, 118.89, 104.60, 56.05, and 25.92. MALDI-TOF-MS: calcd for $C_{15}H_{16}NO_3$ $[M + H]^+$: m/z = 258.1130; found: 258.1132.

Synthesis of L^a. To a mixture of **3** (5.0 g, 19.4 mmol) and 4-methoxybenzaldehyde (1.2 g, 8.8 mmol) in absolute EtOH (120 mL), NaOH (2.5 g, 62.5 mmol) was added. After being stirred for 24 h at 25 °C, NH_4OH (28%, 6.3 mL) was added to the reaction mixture, which was further refluxed for 24 h. The mixture was filtered and washed by H_2O for several times. The residue was further recrystallized from MeCN/$CHCl_3$ to give compound **L^a** as a white solid (3.5 g, 65%). ^1H NMR (400 MHz, $CDCl_3$): δ (*ppm*) 8.64 (s, 2H), 8.59 (d, J = 7.8 Hz, 2H), 7.89 (t, J = 7.8 Hz, 2H), 7.77 (d, J = 7.8 Hz, 2H), 7.35 (m, 4H), 6.95 (d, J = 7.8 Hz, 2H), 6.70 (d, J = 8.6 Hz, 2H), 3.8 (s, 3H), and 3.76 (s, 12H). ^{13}C NMR (100 MHz, $CDCl_3$): δ (ppm) 160.14, 158.42, 156.35, 156.32, 153.18, 149.13, 136.32, 131.45, 129.54, 128.55, 126.08, 120.07, 119.49, 118.75, 114.11, 104.74, 56.22, and 55.31. MALDI-TOF-MS: calcd for $C_{38}H_{33}N_3NaO_5$ $[M + Na]^+$: m/z = 634.2318; found: 634.2315.

Synthesis of 5. To a degassed two-neck flask containing 1,3-diisopropoxybenzene (26.4 g, 136.4 mmol), 100 mL of dry THF was added. *n*-BuLi in hexane (60.0 mL, 150.0 mmol) was added dropwise to the reaction mixture at -20 ˚C, which was subsequently stirred at 25 °C for 2 h. Trimethylborate (19.5 mL, 175.0 mmol) was added to the mixture at -65 °C, which was further stirred at 25 ˚C for 12 h. The reaction mixture was quenched upon addition of conc. H_2SO_4. The solvent was evaporated using rotary evaporator. The mixture was extracted with ethyl acetate and H_2O. The combined organic extract was dried over $MgSO_4$ and then evaporated to dryness under reduced pressure. The crude product was purified by chromatography on silica eluting with

hexane/ethyl acetate (4/1, v/v), and the solvent was evaporated to afford **5** (31.0 g, 95%). ^{1}H NMR (400 MHz, $CDCl_3$): δ (ppm) 7.50 (s, 2H), 7.32 (t, J = 8.4 Hz, 1H), 6.59 (d, J = 8.4 Hz, 2H), 4.69 (septet, J = 6.1 Hz, 2H), and 1.40 (d, J = 6.0 Hz, 12H). ^{13}C NMR (100 MHz, $CDCl_3$): δ (ppm) 164.05, 132.65, 106.51, 71.70, and 22.14. MALDI-TOF-MS: calcd for $C_{12}H_{20}BO_4$ $[M + H]^+$: *m/z* = 239.1451; found: 239.1497.

Synthesis of 6. To a degassed two-neck flask containing **2** (20.0 g, 100.3 mmol), **5** (27.0 g, 113.7 mmol), Na_2CO_3 (35.5 g, 335.0 mmol), and $Pd(PPh_3)_4$ (1.2 g, 1.0 mmol), a mixed solvent (140 mL) of *t*-BuOH/PhMe/H_2O (1:3:3, v/v/v) was added after being purged with N_2 for 30 min. The reaction mixture was refluxed for 24 h under N_2. After cooling to 25 °C, the mixture was extracted with CH_2Cl_2 and the combined organic extract was dried over $MgSO_4$ and then evaporated to dryness under reduced pressure. The residue was subjected to column chromatography (SiO_2, Hexane/EA = 9:1) and then recrystallized from hexane to give **6** (29.6 g, 94%). ^{1}H NMR (400 MHz, $CDCl_3$): δ (ppm) 7.94 (d, J = 7.8 Hz, 1H), 7.79 (t, J = 7.8 Hz, 1H), 7.46 (d, J = 7.8 Hz, 1H), 7.26 (t, J = 8.4 Hz, 1H), 6.64 (d, J = 8.4 Hz, 2H), 4.45 (septet, J = 6.1 Hz, 2H), 2.71 (s, 3H), and 1.16 (d, J = 6.1 Hz, 12H). ^{13}C NMR (100 MHz, $CDCl_3$): δ (ppm) 201.37, 156.94, 154.66, 153.13, 135.78, 130.23, 129.66, 121.73, 11921, 107.52, 71.15, 26.08, and 22.20. MALDI-TOF-MS: calcd for $C_{19}H_{23}NNaO_3$ $[M + Na]^+$: *m/z* = 336.1570; found: 336.1603.

Synthesis of L^b. To a mixture of **6** (3.2 g, 10.1 mmol) and 4-methoxybenzaldehyde (0.6 g, 4.6 mmol) in absolute EtOH (70 mL), NaOH (1.1 g, 27.6 mmol) was added. After being stirred at 25 °C for 24 h, NH_4OH (28%, 3.2 mL) was added to the mixture, which was further refluxed for an additional 24 h. The mixture was filtered and washed by H_2O for several times. The residue was recrystallized from MeCN/$CHCl_3$ to give $\mathbf{L^b}$ (2.0 g, 60%) as a white solid. ^{1}H NMR (400 MHz, $CDCl_3$): δ (ppm) 8.68 (s, 2H), 8.58 (d, J = 7.8 Hz, 2H), 7.87 (t, J = 7.8 Hz, 2H), 7.75 (d, J = 8.2 Hz, 2H), 7.36 (d, J = 8.2 Hz, 2H), 7.27 (t, J = 8.4 Hz, 2H), 6.94 (d, J = 8.4 Hz, 2H), 6.68 (d, J = 8.4 Hz, 4H), 4.45 (septet, J = 6.1 Hz 4H), 3.80 (s, 3H), and 1.17 (d, J = 6.1 Hz, 24H). ^{13}C NMR (100 MHz, $CDCl_3$): δ (ppm) 160.11, 157.00, 156.49, 155.57, 154.34, 149.16, 135.74, 131.22, 129.04, 128.36, 126.44, 123.05, 118.76, 118.35, 114.01, 108.06, 71.30, 55.20, and 22.15. MALDI-TOF-MS: calcd for $C_{46}H_{50}N_3O_5$ $[M + H]^+$: *m/z* = 724.3750; found: 724.3745, calcd for $C_{46}H_{49}N_3NaO_5$ $[M + Na]^+$: *m/z* = 746.3570; found: 746.3573.

Synthesis of L^c. To a mixture of **6** (5.2 g, 16.7 mmol) and 4-bromobenzaldehyde (1.4 g, 7.7 mmol) in absolute EtOH (300 mL), NaOH (2.0 g, 51.8 mmol) was added. After being stirred at 25 °C for 24 h, NH_4OH (28%, 5.5 mL) was added to the mixture, which was refluxed for an additional 24 h. The mixture was filtered and washed by H_2O for several times. The residue was recrystallized from MeCN/$CHCl_3$ to give $\mathbf{L^c}$ (4.0 g, 67%) as a white solid. ^1H NMR (400 MHz, $CDCl_3$): δ (ppm) 8.68 (s, 2H), 8.57 (d, J = 7.8 Hz, 2H), 7.88 (t, J = 7.8 Hz, 2H), 7.65 (d, J = 8.2 Hz, 2H), 7.55 (d, J = 8.2 Hz, 2H), 7.37 (d, J = 7.6 Hz, 2H), 7.27 (t, J = 8.4 Hz, 2H), 6.68 (d, J = 8.4 Hz, 24H), 4.46 (septet, J = 6.1 Hz 4H), and 1.17 (d, J = 6.1 Hz, 12H). ^{13}C NMR (100 MHz, $CDCl_3$): δ (ppm) 157.20, 156.98, 155.47, 154.62, 148.67, 138.12, 136.04, 131.98, 129.32, 129.02, 126.85, 123.11, 123.05, 119.00, 118.83, 108.16, 71.46, and 22.36. MALDI-TOF-MS: calcd for $C_{45}H_{47}BrN_3O_4$ $[M + H]^+$: m/z = 774.2727; found: 774.2763.

Synthesis of 7. To a degassed flask containing $\mathbf{L^c}$ (2.0 g, 2.6 mmol), bis(pinacolato)diboron (1.2 g, 4.7 mmol), KOAc (1.0 g, 10.4 mmol), and $Pd(dppf)_2Cl_2$ (230 mg, 0.3 mmol), anhydrous 1,4-dioxane (20 mL) was added. The mixture was stirred at 80 °C for 4 h under N_2. After cooling to 25 ˚C, the reaction mixture was poured into water and extracted with CH_2Cl_2. The combined organic extract was dried over anhydrous $MgSO_4$ and evaporated to dryness under reduced pressure. The residue was precipitated from MeCN to give **7** (1.6 g, 73%). ^1H NMR (400 MHz, $CDCl_3$): δ (ppm) 8.73 (s, 2H), 8.57 (d, J = 7.8 Hz, 2H), 7.89–7.79 (m, 6H), 7.37 (d, J = 7.7 Hz, 2H), 7.26 (t, J = 8.4 Hz, 2H), 6.67 (d, J = 8.4 Hz, 4H), 4.46 (septet, J = 6.1 Hz, 4H), 1.36 (s, 12H), and 1.18 (d, J = 6.1 Hz, 24H). ^{13}C NMR (100 MHz, $CDCl_3$): δ (ppm) 157.19, 156.90, 155.69, 154.54, 149.64, 141.64, 136.00, 135.27, 129.28, 126.78, 126.63, 123.04, 119.18, 119.04, 108.15, 84.02, 71.45, 25.04, and 22.36. MALDI-TOF-MS: calcd for $C_{51}H_{59}BN_3O_6$ $[M + H]^+$: m/z = 820.4497; found: 820.4482.

Synthesis of F^1. To a degassed two-neck flask containing 1,3,5-tribromobenzene (119.3 mg, 0.3 mmol), **7** (1.5 g, 1.8 mmol), and Na_2CO_3 (1.2 g, 11.3 mmol), a mixed solvent (28 mL) of *t*-BuOH/PhMe/H_2O (1:3:3, v/v/v) was added. After being purged with N_2 for 30 min, $Pd(PPh_3)_4$ (68.1 mg, 0.06 mmol) was added into the mixture, which was refluxed for 72 h under N_2. After cooling to 25 °C, the mixture was extracted with CH_2Cl_2 and the combined organic extract was dried over $MgSO_4$ and then evaporated to dryness under reduced pressure. The residue was subjected to column chromatography (Al_2O_3, CH_2Cl_2/Hexane = 3:2) and then precipitated from

CH_3CN to give **F¹** (432.7 mg, 53%) as a white solid. ^{1}H NMR (400 MHz, $CDCl_3$): *δ* (ppm) 8.79 (s, 6H), 8.60 (d, *J* = 7.8 Hz, 6H), 7.93 (d, *J* = 8.4 Hz, 6H), 7.89 (t, *J* = 7.8 Hz, 6H), 7.86 (s, 3H), 7.78 (d, *J* = 8.4 Hz, 6H), 7.39 (d, *J* = 7.8 Hz, 6H), 7.26 (t, *J* = 8.3 Hz, 6H), 6.68 (d, *J* = 8.3 Hz, 12H), 4.47 (septet, *J* = 6.1 Hz, 12H), and 1.20 (d, *J* = 6.1 Hz, 72H). ^{13}C NMR (100 MHz, $CDCl_3$): *δ* (ppm) 157.22, 156.90, 155.68, 154.60,149.35, 142.00, 141.35, 138.37, 136.04, 129.27, 127.91, 127.78, 126.77, 125.37, 123.15, 119.03, 108.22, 71.52, 29.83, and 22.39. MALDI-TOF-MS: calcd for $C_{141}H_{141}N_9O_{12}$ $[M]^+$: *m/z* = 2153.0733; found: 2153.0746.

Synthesis of F². By a similar procedure to that for **F¹**, **F²** (1.0 g, 0.5 mmol) was obtained in 94% yield from 1,3,5-tribromo-2,4,6-trimethoxybenzene (0.2 g, 0.5 mmol), **7** (1.6 g, 1.9 mmol), Na_2CO_3 (1.8 g, 17.2 mmol), and $Pd(PPh_3)_4$ (0.1 g, 0.1 mmol). ^{1}H NMR (500 MHz, $CDCl_3$): *δ* (ppm) 8.78 (s, 6H), 8.61 (dd, *J* = 7.9 and 1.0 Hz, 6H), 7.89 (t, *J* = 7.8 Hz, 6H), 7.84 (d, *J* = 8.4 Hz, 6H), 7.62 (d, *J* = 8.4 Hz, 6H), 7.39 (dd, *J* = 7.8 and 1.0 Hz, 6H), 7.26 (t, *J* = 8.3 Hz, 6H), 6.68 (d, *J* = 8.4 Hz, 12H), 4.47 (septet, *J* = 6.1 Hz, 12H), 3.09 (s, 9H), and 1.19 (d, *J* = 6.1 Hz, 72H). ^{13}C NMR (125 MHz, $CDCl_3$): *δ* (ppm) 157.20, 156.75, 156.21, 155.69, 154.54, 149.99, 137.93, 136.04, 134.81, 131.03, 129.26, 127.07, 126.73, 125.62, 123.12, 119.31, 118.97, 108.23, 71.52, 60.74, and 22.35. MALDI-TOF: calcd for $C_{144}H_{148}N_9O_{15}$ $[M + H]^+$: *m/z* = 2244.1128; found: 2244.1296.

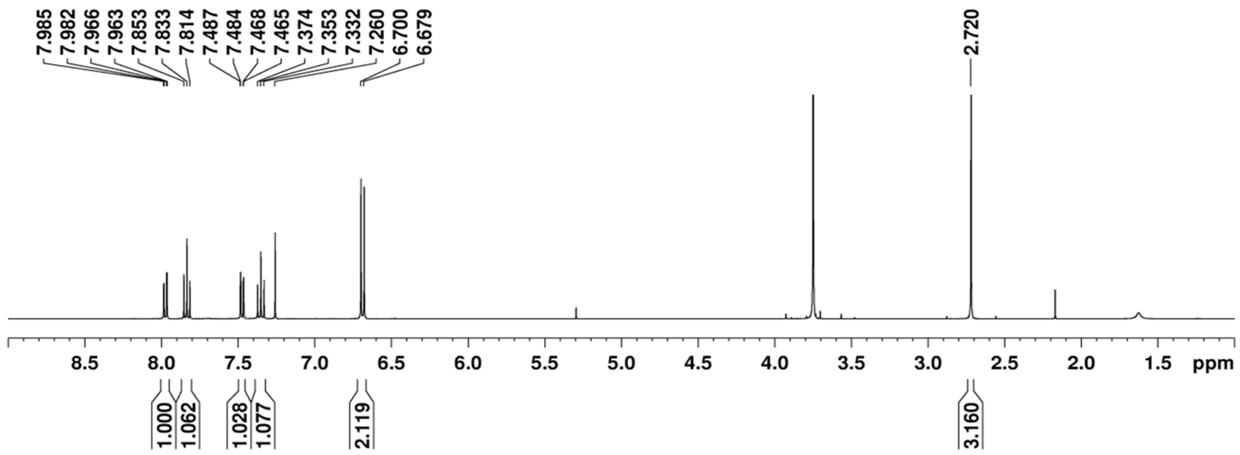

Figure S1. ^{1}H NMR spectrum (400 MHz, $CDCl_3$) of **3**.

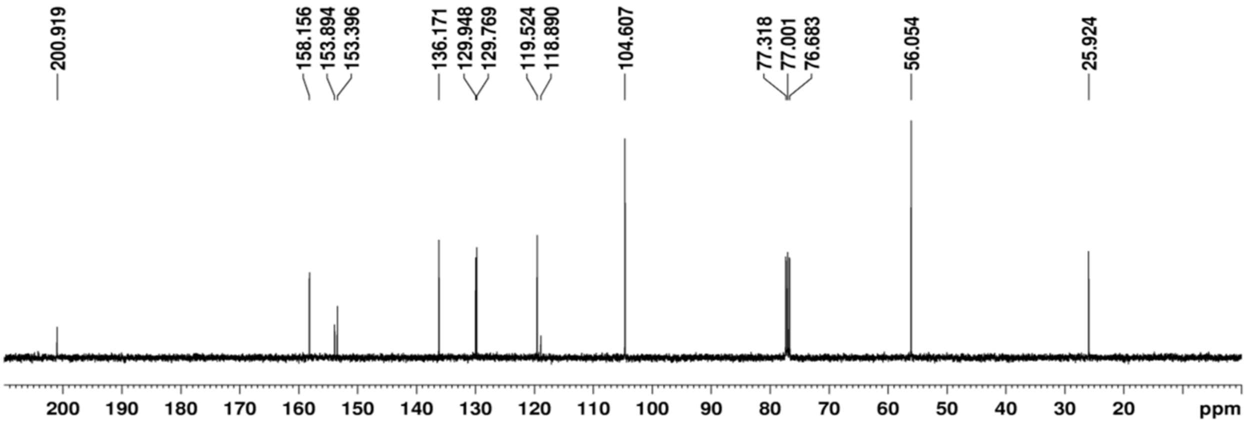

Figure S2. ^{13}C NMR spectrum (100 MHz, $CDCl_3$) of **3**.

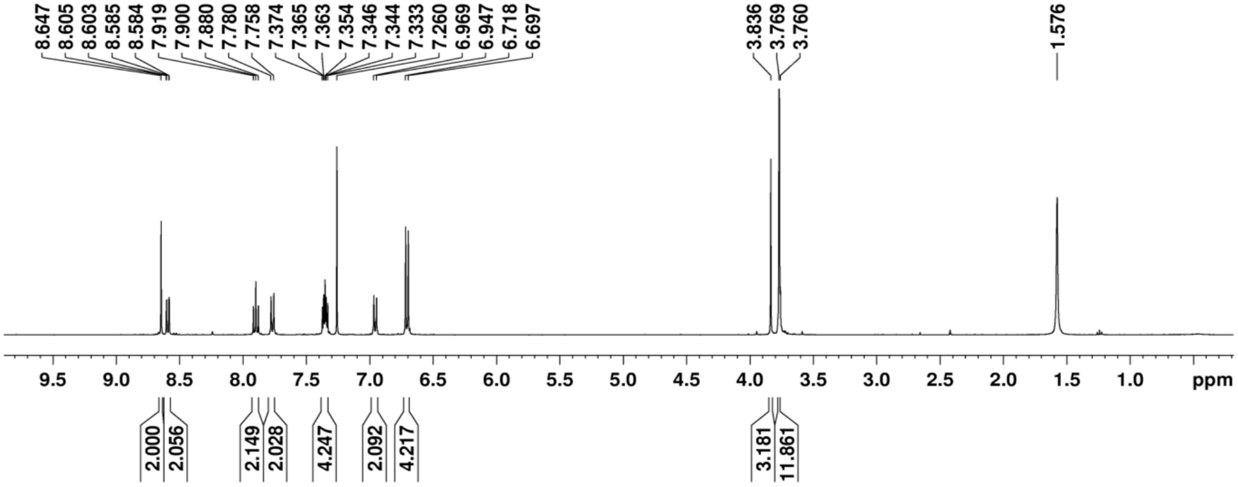

Figure S3. ^{1}H NMR spectrum (400 MHz, $CDCl_3$) of **L^a**.

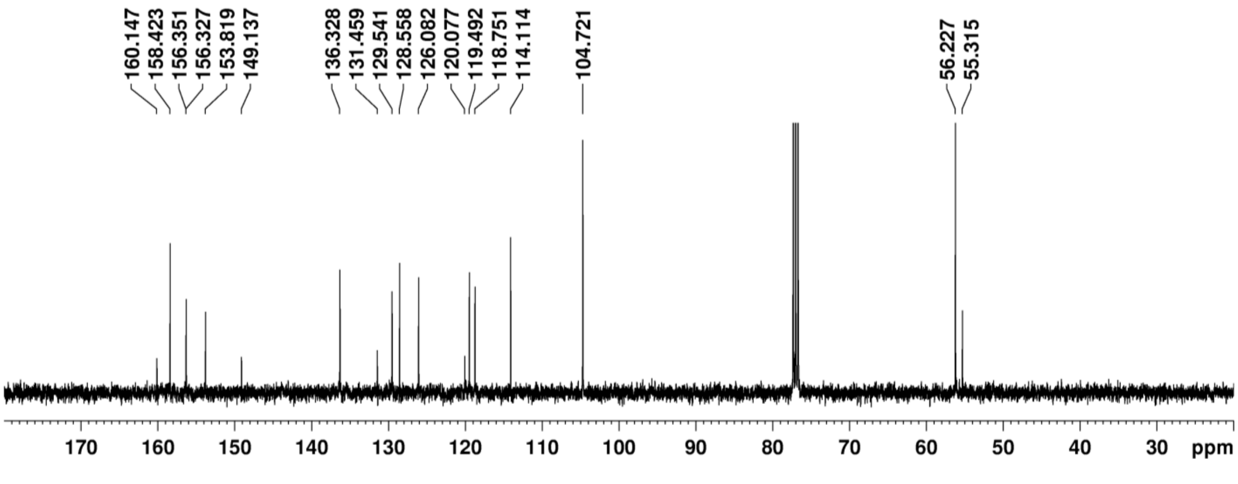

Figure S4. ^{13}C NMR spectrum (100 MHz, $CDCl_3$) of **L^a**.

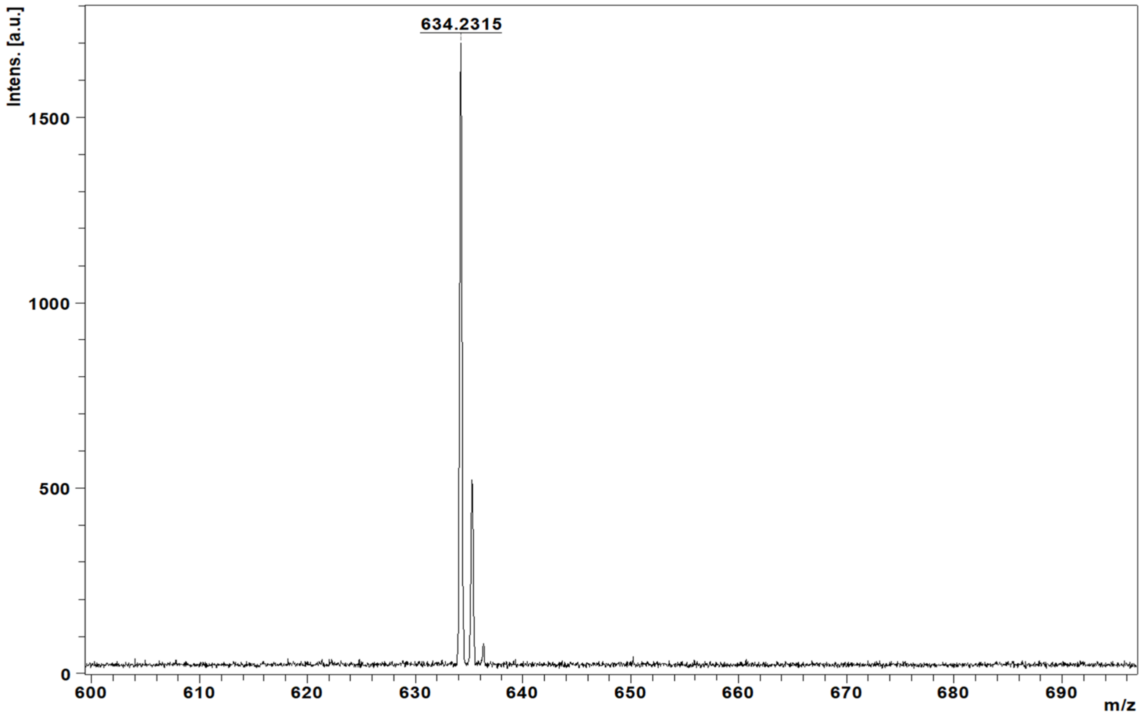

Figure S5. MALDI-TOF MS spectrum of **L^a**.

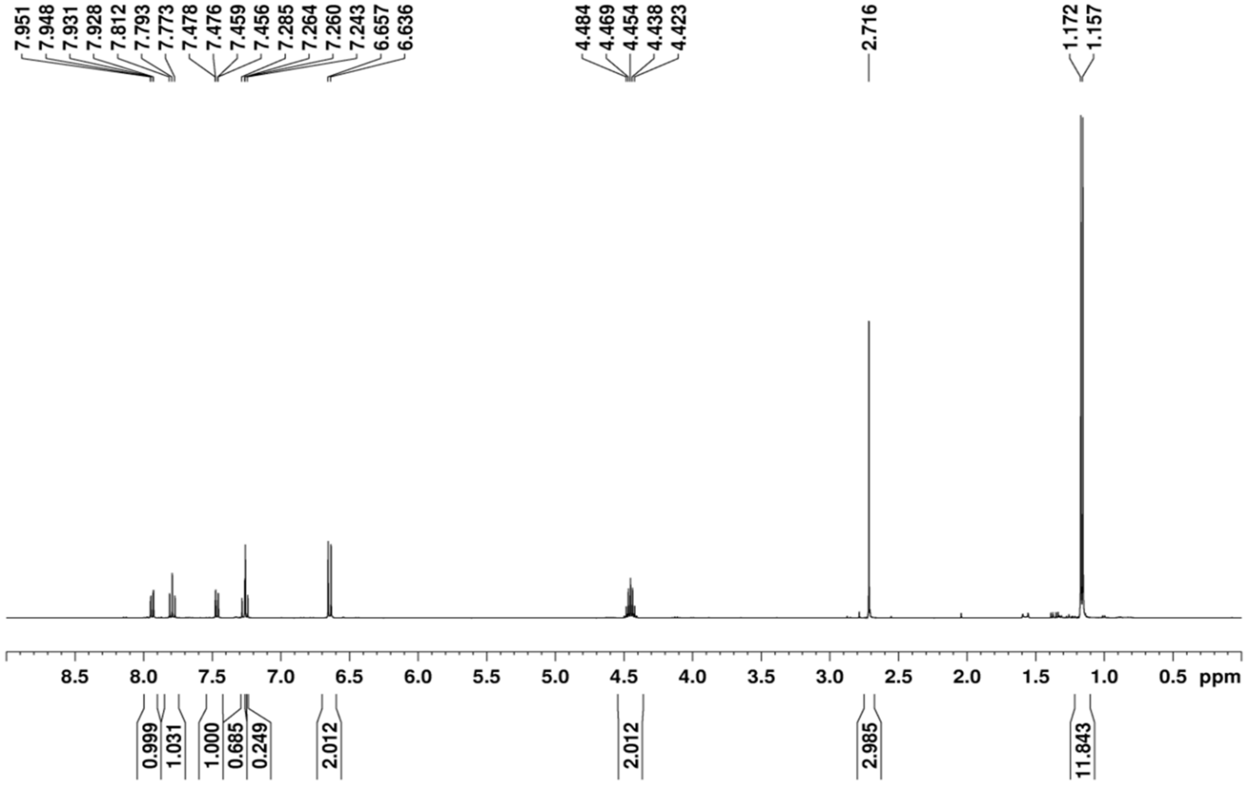

Figure S6. ^{1}H NMR spectrum (400 MHz, $CDCl_3$) of **6**.

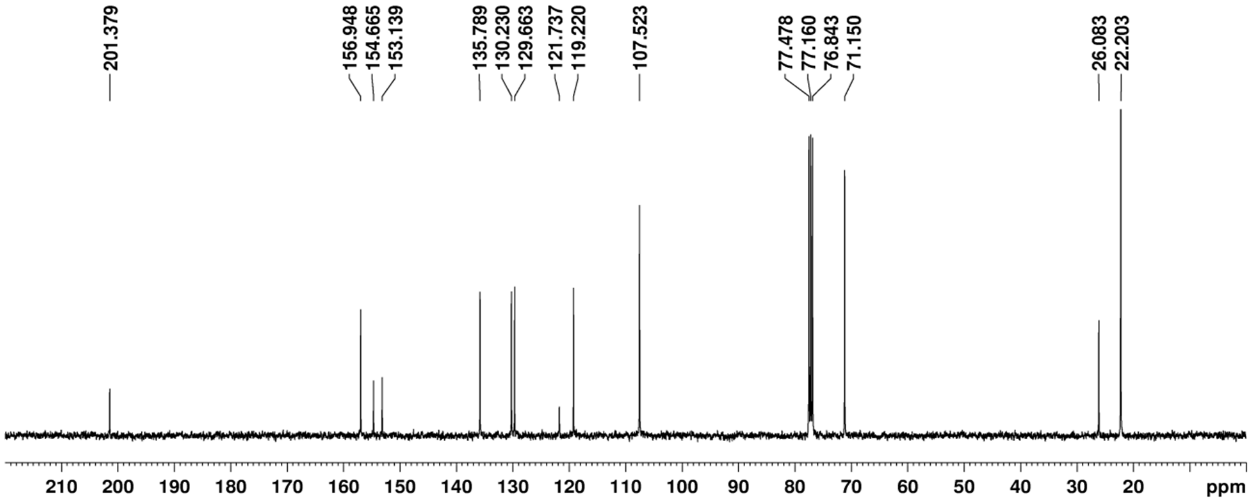

Figure S7. ^{13}C NMR spectrum (100 MHz, $CDCl_3$) of **6**.

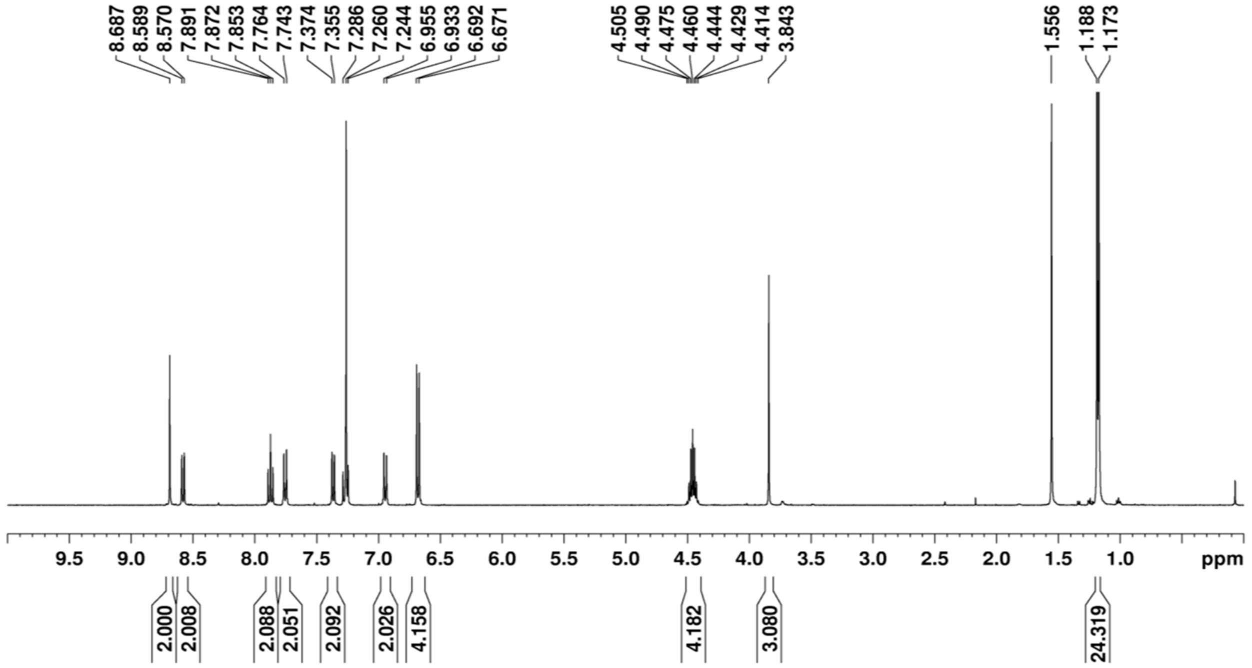

Figure S8. ^{1}H NMR spectrum (400 MHz, $CDCl_3$) of $\mathbf{L^b}$.

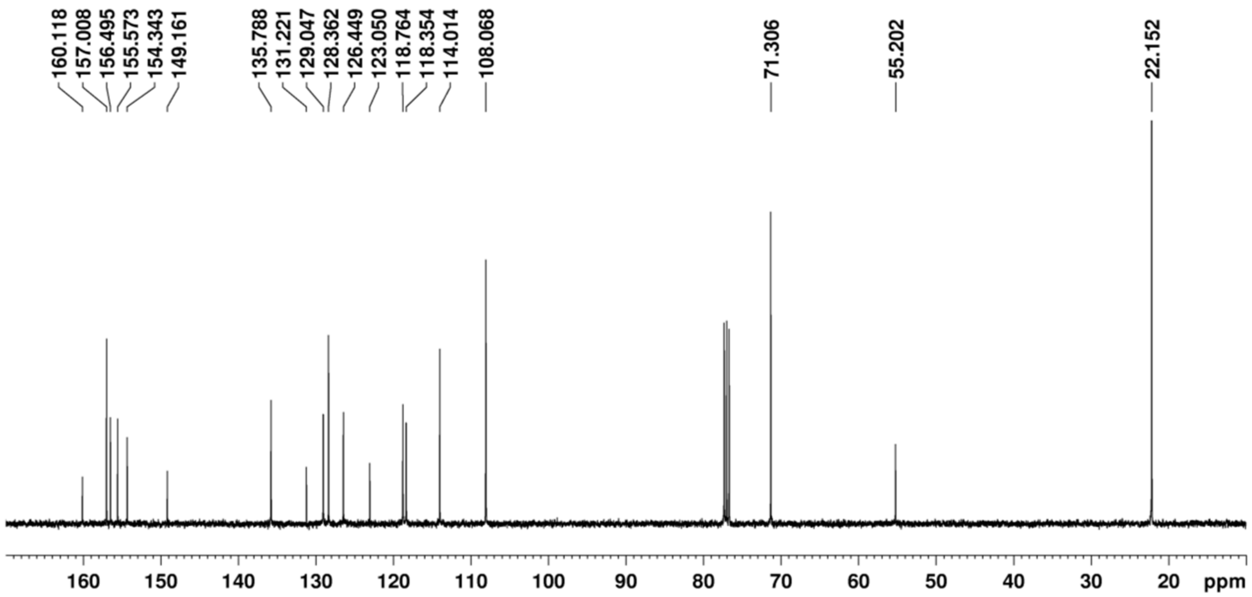

Figure S9. ^{13}C NMR spectrum (100 MHz, $CDCl_3$)of $\mathbf{L^b}$.

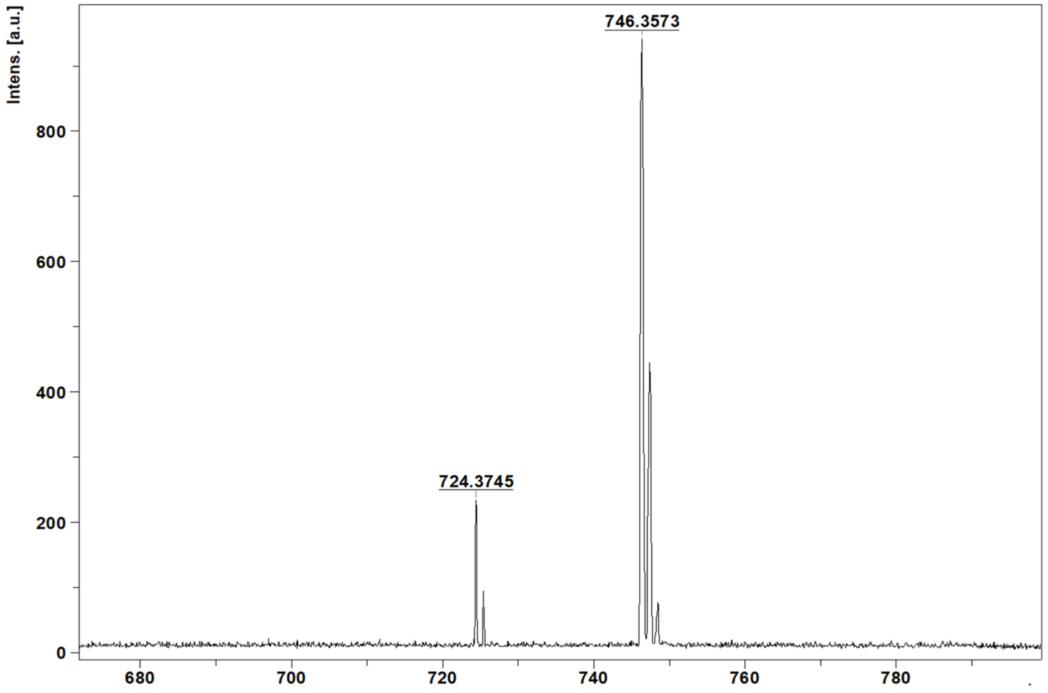

Figure S10. MALDI-TOF MS spectrum of $\mathbf{L^b}$.

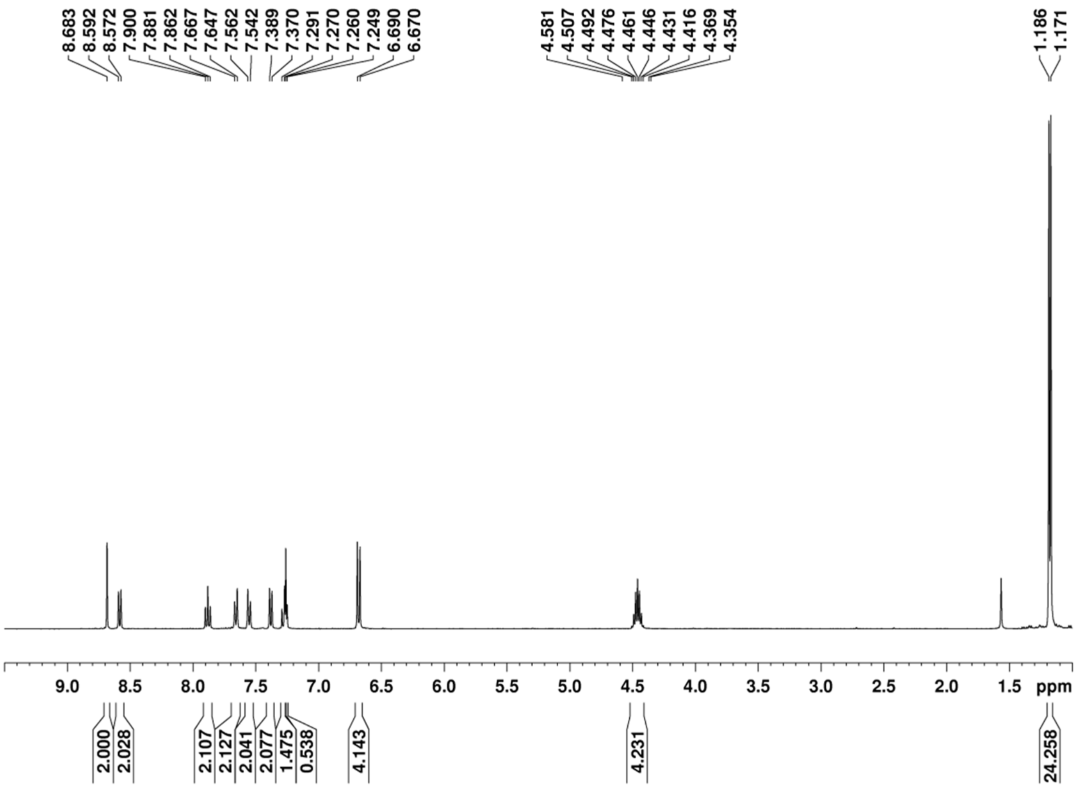

Figure S11. ^{1}H NMR spectrum (400 MHz, $CDCl_3$) of **L^{c}**.

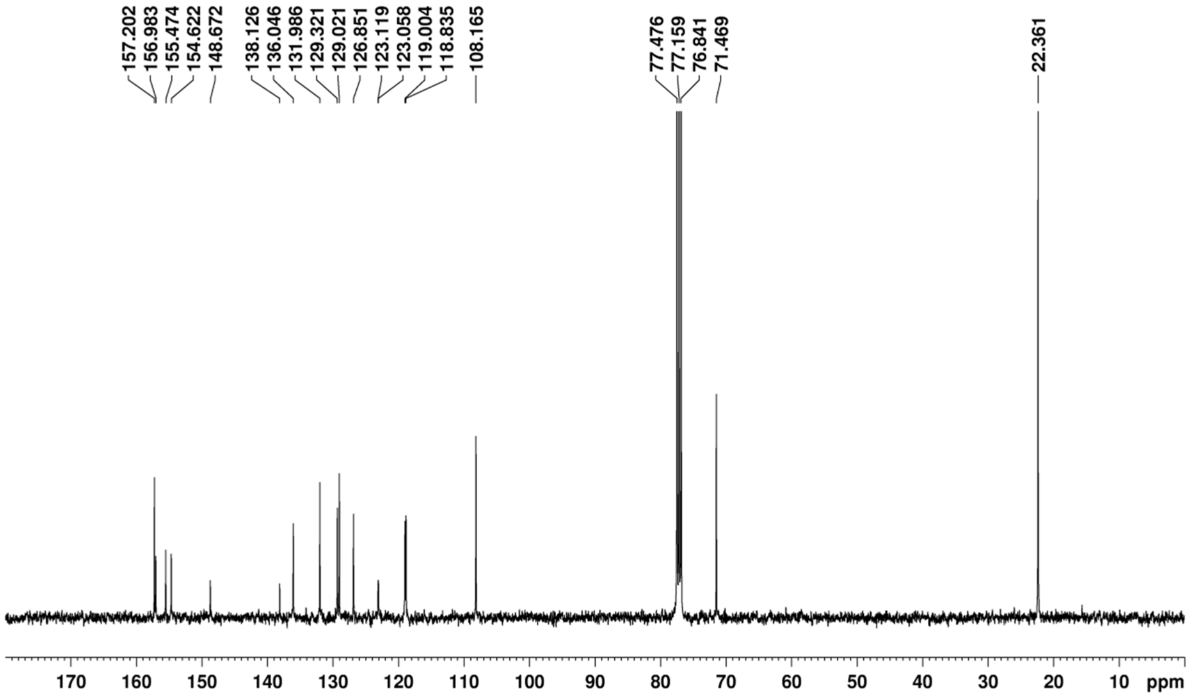

Figure S12. ^{13}C NMR spectrum (100 MHz, $CDCl_3$) of **L^{c}**.

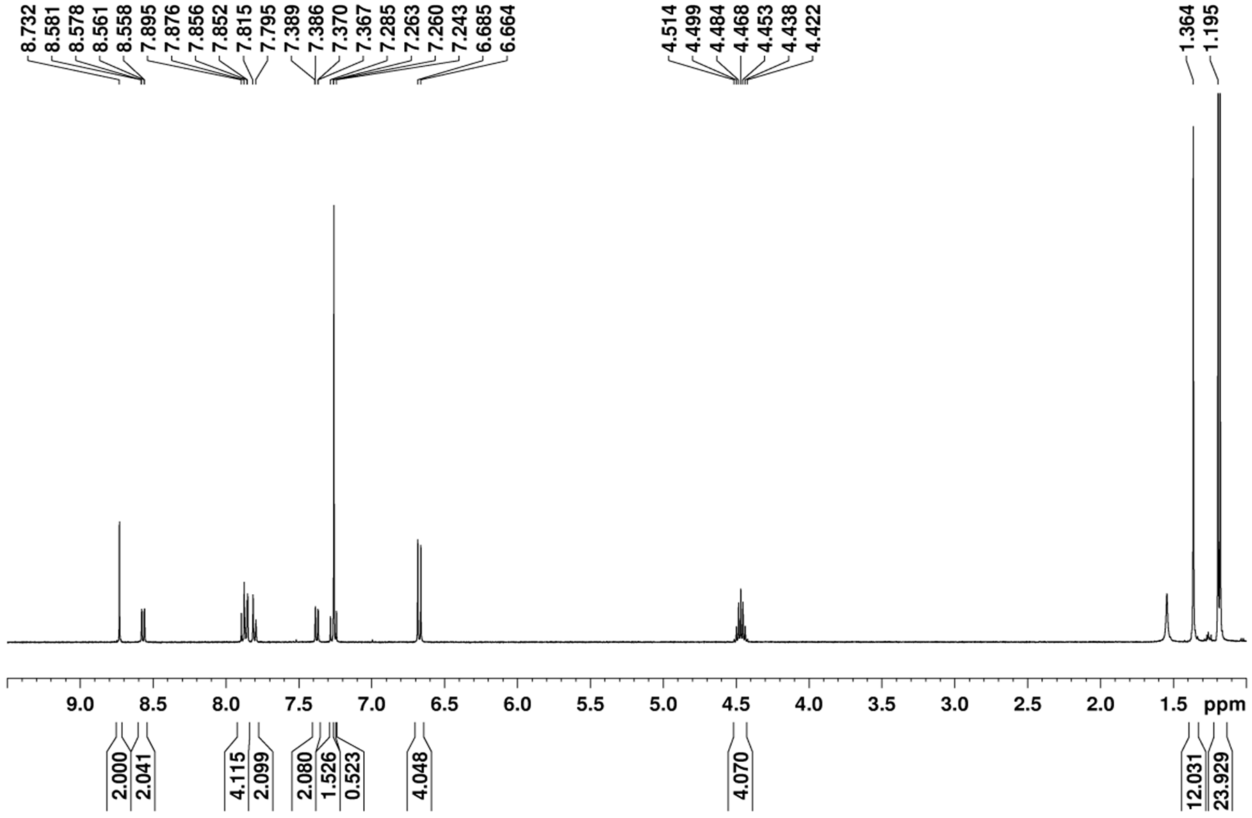

Figure S13. ^{1}H NMR spectrum (400 MHz, $CDCl_3$) of **7**.

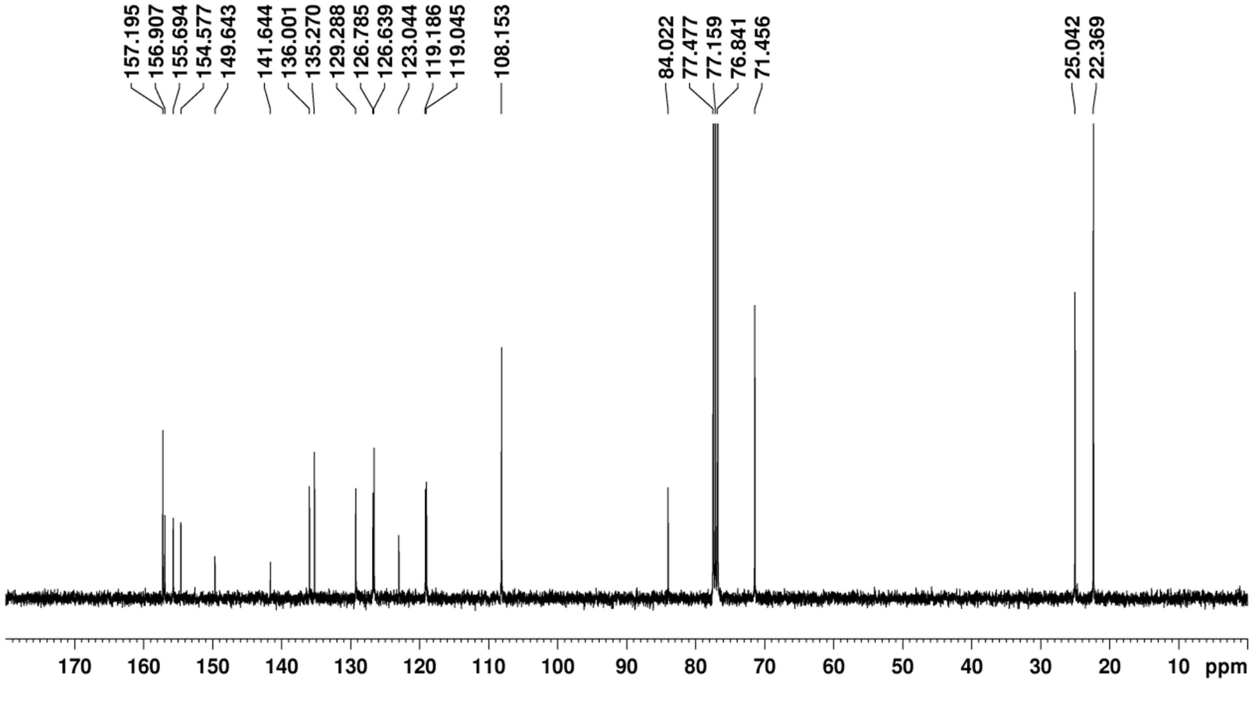

Figure S14. ^{13}C NMR spectrum (100 MHz, $CDCl_3$) of **7**.

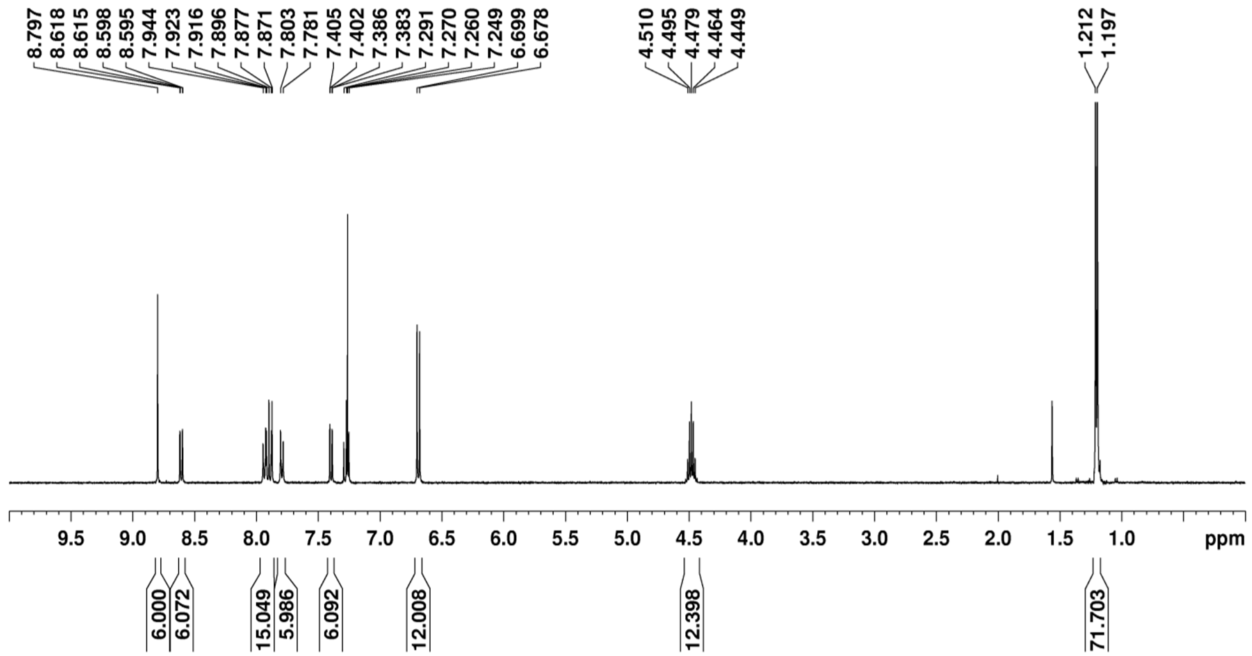

Figure S15. ^{1}H NMR spectrum (400 MHz, $CDCl_3$) of **F[1]**.

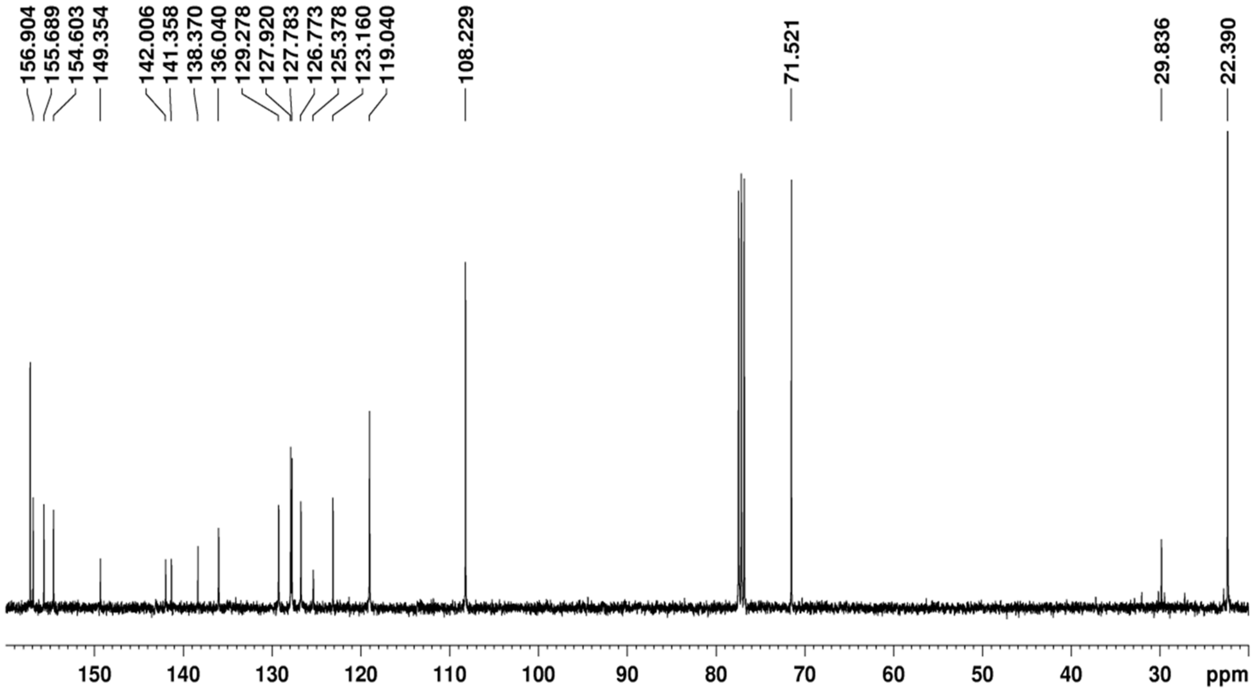

Figure S16. ^{13}C NMR spectrum (100 MHz, $CDCl_3$) of **F[1]**.

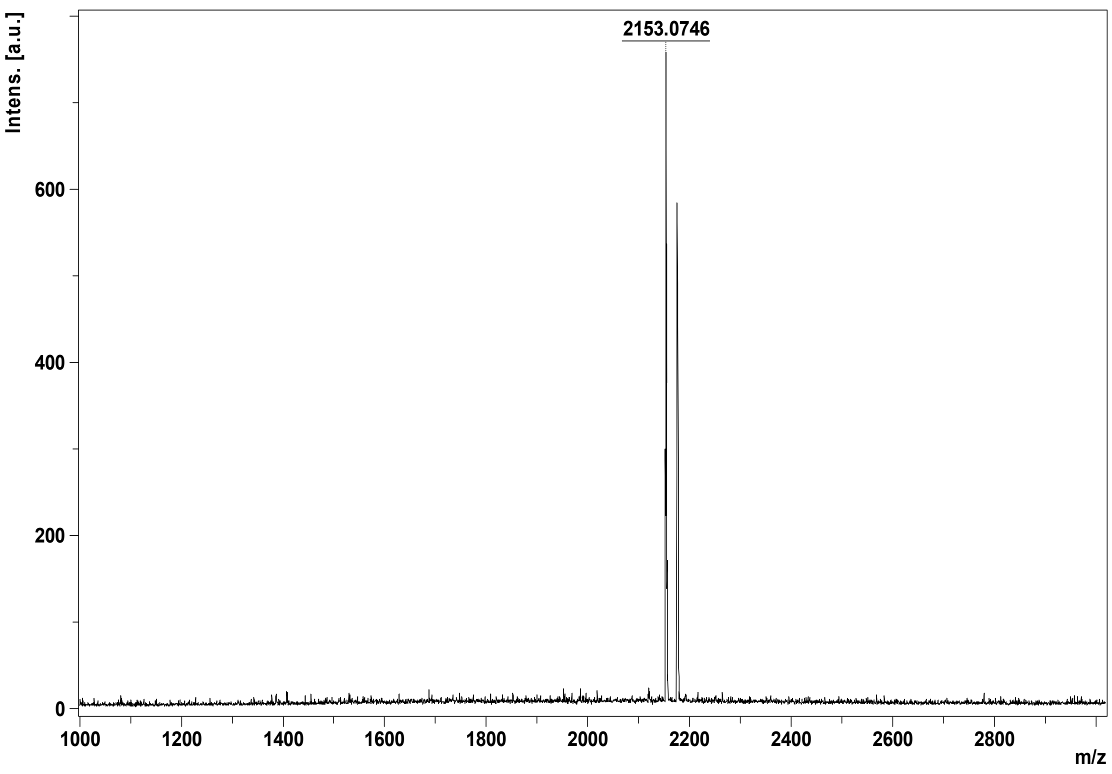

Figure S17. MALDI-TOF MS spectrum of $\mathbf{F^1}$.

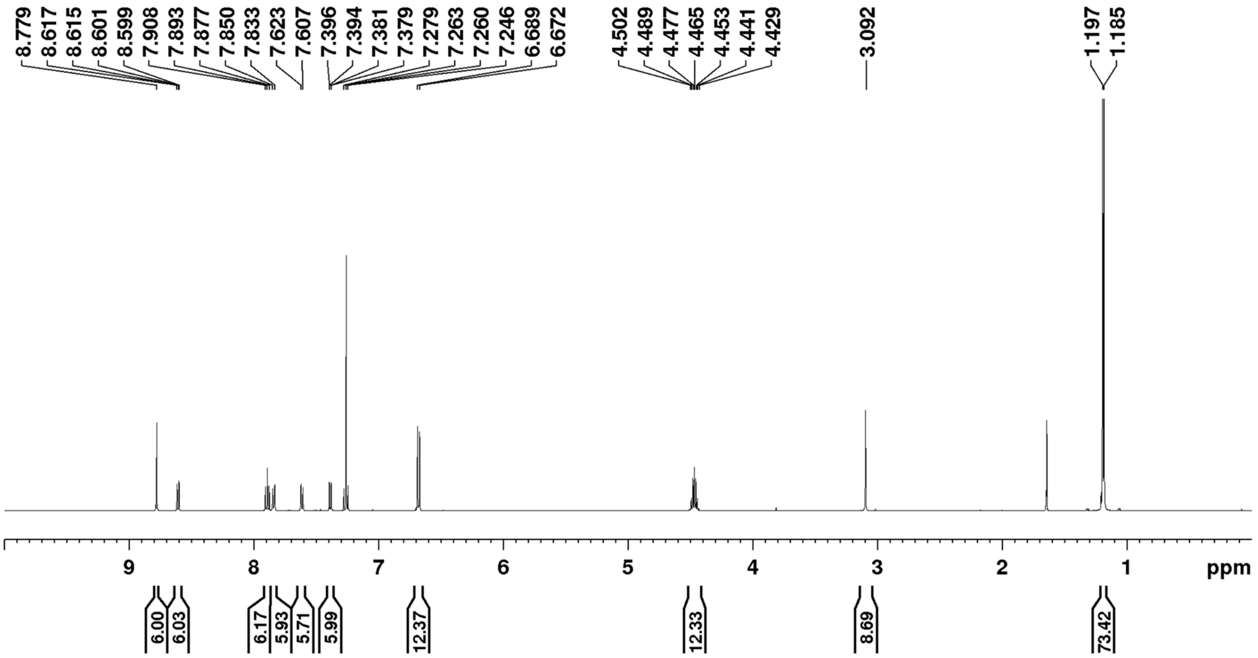

Figure S18. ^{1}H NMR spectrum (500 MHz, $CDCl_3$) of $\mathbf{F^2}$.

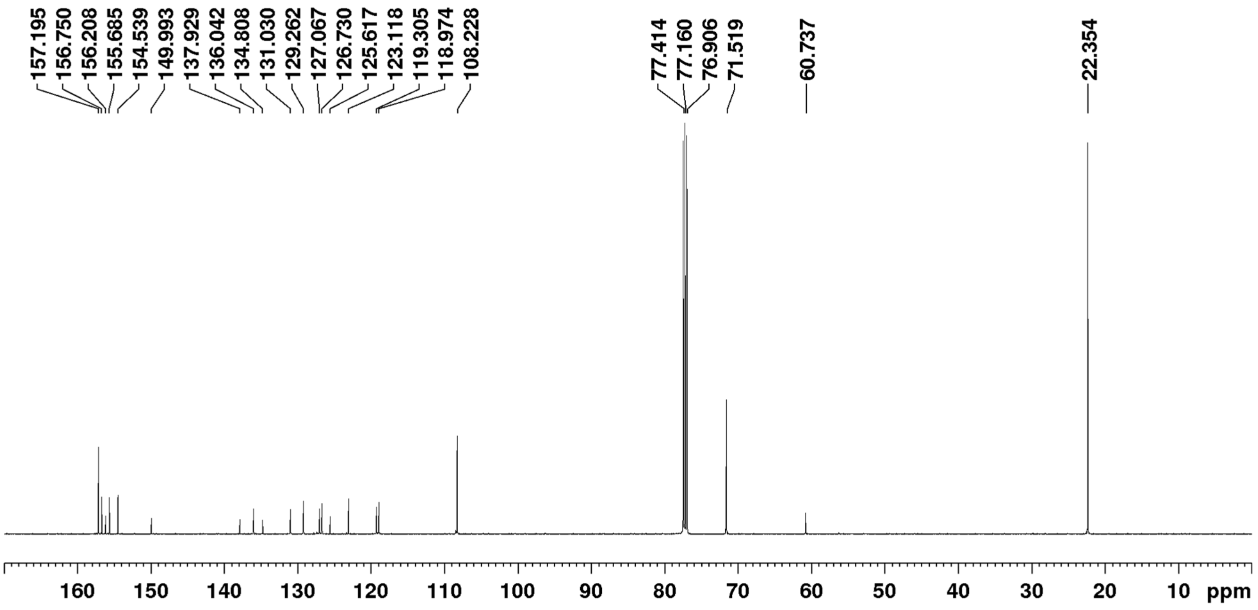

Figure S19. ^{13}C NMR spectrum (125 MHz, $CDCl_3$) of $\mathbf{F^2}$.

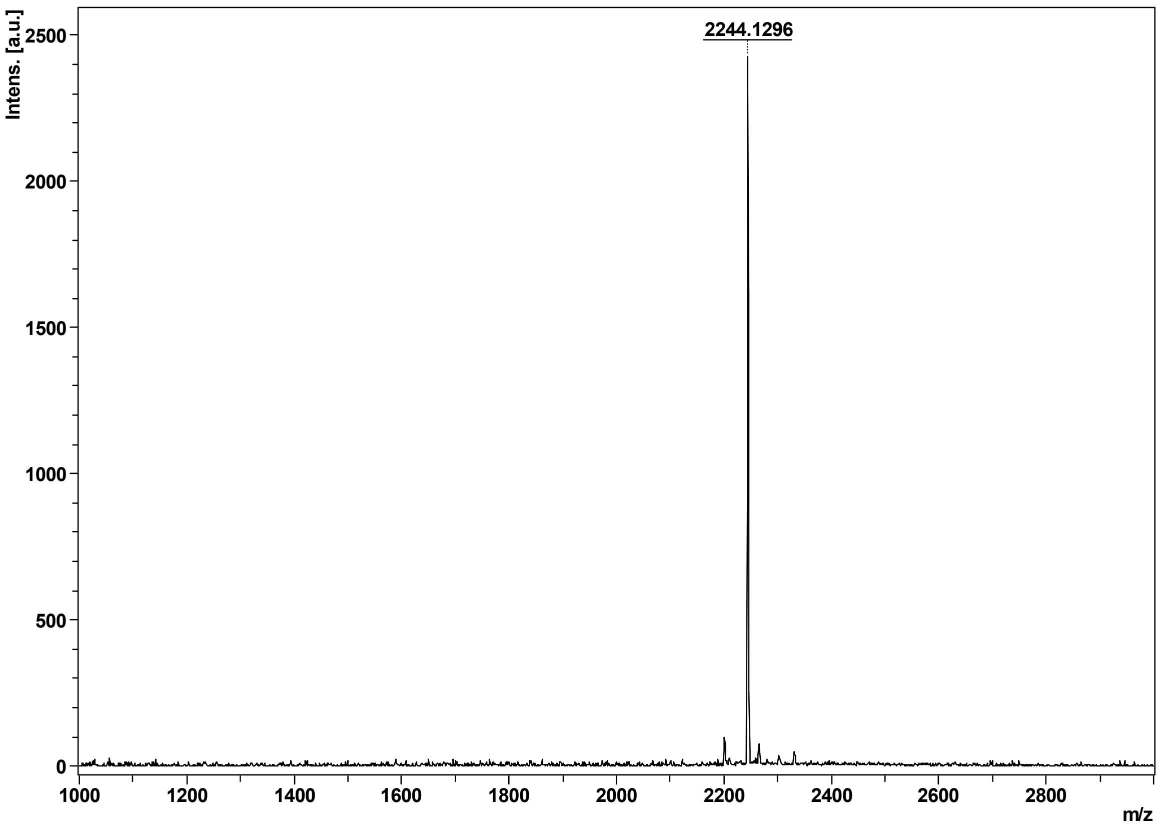

Figure S20. MALDI-TOF MS spectrum of $\mathbf{F^2}$.

General Procedure for Complexation. To a stirred solution of $Cd(NO_3)_2·4H_2O$ in MeOH, a solution of ligand(s) in $CHCl_3$ was added. The mixture was stirred at 25 °C for 30 min and then a slight excess of NH_4PF_6 was added to precipitate the counterion-exchanged complex (PF_6^-), which was filtered, washed with H_2O and MeOH, and then dried *in vacuo*. An MeCN solution of the resultant complex was heated at 80 ˚C for a certain period. The reaction was monitored by NMR and ESI-MS.

Synthesis of [CdL$^a{}_2$](OTf)$_2$. To an MeCN solution (2 mL) of $Cd(OTf)_2$ (3.4 mg, 8.4 µmol), **L**^{a} (10.0 mg, 16.3 µmol) was added. The mixture was stirred at 60 °C for 1 h. The solvent was evaporated to give the residue, which was washed with H_2O/MeOH and then dried *in vacuo* to afford [Cd**L**$^a{}_2$](OTf)$_2$ as a white solid in 98% yield (13.0 mg, 8.0 µmol). ^{1}H NMR (400 MHz, CD_3CN): δ (ppm) 8.48 (d, J = 8.0 Hz, 4H), 8.33 (s, 4H), 8.11 (d, J = 8.8 Hz, 4H), 8.0 (t, J = 8.0 Hz, 4H), 7.34 (d, J = 8.8 Hz, 4H), 7.0 (d, J = 8.0 Hz, 4H), 6.3 (t, J = 8.5 Hz, 4H), 5.97 (d, J = 8.0 Hz, 8H), 4.0 (s, 6H), and 2.8 (s, 24H). ^{13}C NMR (100 MHz, CD_3CN): δ (ppm) 162.84, 158.64, 157.60, 153.38, 150.19, 149.80, 140.13, 131.79, 130.88, 130.41, 130.20, 122.70, 121.98, 116.60, 115.96, 103.88, 56.42, and 54.96. ESI-MS (*m/z*): 1485.3309 [M – OTf]$^+$ (calcd *m/z* = 1485.3394) and 668.1987 [M – 2OTf]$^{2+}$ (calcd *m/z* = 668.1936).

Synthesis of [CdL^b](OTf)$_2$. To an MeCN solution (2 mL) of $Cd(OTf)_2$ (34.2 mg, 83.3 µmol), **L**^{b} (56.9 mg, 73.6 µmol) was added. The mixture was stirred at 25 °C for 30 min. The solvent was evaporated to give the residue, which was washed with H_2O/MeOH and then dried *in vacuo* to afford [Cd**L**^{b}](OTf)$_2$ as a white solid in quantitative yield (83.0 mg, 73.2 µmol). ^{1}H NMR (400 MHz, CD_3CN): δ (*ppm*) 8.78 (s, 2H), 8.68 (d, J = 8.0 Hz, 2H), 8.25 (t, J = 8.0 Hz, 2H), 8.11 (d, J = 8.6 Hz, 2H), 7.55 (d, J = 8.0, 2H), 7.40 (t, J = 8.0 Hz, 2H), 7.22 (d, J = 8.6 Hz, 2H), 6.79 (b, J = 8.0 Hz, 4H), 4.51 (septet, J = 6.0 Hz, 4H), 3.93 (s, 3H), 1.10 (d, J = 6.1 Hz, 12H), and 1.01 (d, J = 6.1 Hz, 12H). ^{13}C NMR (100 MHz, CD_3CN): δ (ppm) 163.04, 158.09, 157.69, 155.06, 150.48, 149.58, 141.20, 132.91, 131.23, 130.44, 129.11, 123.19, 121.68, 120.90, 115.82, 109.29, 73.09, 56.31, 22.52, and 21.90. ESI-MS (*m/z*): 986.2211 [M − OTf]$^+$ (calcd *m/z* = 986.2224) and 418.6379 [M − 2OTf]$^+$ (calcd *m/z* = 418.6351).

Synthesis of [CdL^{c}L^d](PF$_6$)$_2$. By the general procedure without heating, [Cd**L**^{c}**L**^{d}](PF$_6$)$_2$ was obtained in quantitative yield (105.5 mg, 69.7 µmol) from **L**^{c} (54.4 mg, 70.4 µmol), **L**^{d} (23.9 mg,

70.4 μmol), $Cd(NO_3)_2·4H_2O$ (22.8 mg, 73.9 μmol), NH_4PF_6 (280 mg, 1.7 mmol), and 10 mL of MeOH/$CHCl_3$ (1:1, v/v). ^{1}H NMR (400 MHz, CD_3CN): δ (ppm) 8.96 (s, 2H), 8.67 (d, J = 8.6 Hz, 2H), 8.44 (d, J = 8.5 Hz, 2H), 8.33 (s, 2H), 8.15–8.07 (m, 8H), 7.94 (d, J = 8.4 Hz, 2H), 7.90 (d, J = 8.5 Hz, 2H), 7.44 (dd, J = 8.6 and 4.6 Hz, 24H), 7.30 (d, J = 8.4 Hz, 2H), 6.68 (t, J = 8.5 Hz, 2H), 5.78 (d, J = 8.5 Hz, 4H), 3.98 (s, 3H), 3.70 (septet, J = 6.0 Hz, 4H), 0.62 (d, J = 6.1 Hz, 12H), and 0.54 (d, J = 6.1 Hz, 12H). ^{13}C NMR (125 MHz, CD_3CN): δ (ppm) 162.92. 157.88, 156.76, 154.79, 153.80, 152.84, 149.98, 149.45, 149.05, 141.49, 140.65, 136.47, 133.66, 131.78, 131.38, 130.93, 130.39, 129.96, 127.59, 126.08, 124.39, 123.10, 122.92, 121.64, 121.23, 115.95, 109.00, 71.99, 56.43, 22.62, and 21.12. ESI-MS (*m/z*): 1369.2379 $[M - PF_6]^+$ (calcd *m/z* = 1369.2719) and 612.1375 $[M - 2PF_6]^{2+}$ (calcd *m/z* = 612.1538).

Complexation of $\mathbf{F^1}$ and $\mathbf{L^e}$ with $\mathbf{Cd^{II}}$ ions. By the general procedure, after 24 h of heating at 80 °C, a mixture of complexes $[Cd_{18}\mathbf{F^1}_6\mathbf{L^e}_9](PF_6)_{36}$, $[Cd_{12}\mathbf{F^1}_4\mathbf{L^e}_6](PF_6)_{24}$, and $[Cd_6\mathbf{F^1}_2\mathbf{L^e}_3](PF_6)_{12}$ was obtained in quantitative yield from $\mathbf{F^1}$ (50.0 mg, 23.2 μmol), $\mathbf{L^e}$ (24.1 mg, 34.8 μmol), $Cd(NO_3)_2·4H_2O$ (22.0 mg, 71.3 μmol), NH_4PF_6 (250.0 mg, 1.5 mmol), and 10 mL of MeOH/$CHCl_3$ (1:1, v/v).

Synthesis of $\mathbf{[Cd_{30}F^1_8V_6](PF_6)_{60}}$. By the general procedure, $[Cd_{30}\mathbf{F^1}_8\mathbf{V}_6](PF_6)_{60}$ was obtained in 93% yield (51.7 mg, 1.2 μmol) from $\mathbf{F^1}$ (21.6 mg, 10.0 μmol), **V** (19.0 mg, 7.5 μmol), $Cd(NO_3)_2·4H_2O$ (12.2 mg, 39.5 μmol), and NH_4PF_6 (352.2 mg, 2.2 mmol). ^{1}H NMR (400 MHz, CD_3CN): δ (ppm) 9.10 (s, 48H), 8.97 (s, 24H), 8.90 (d, J = 8.2 Hz, 24H), 8.75 (d, J = 8.2 Hz, 24H), 8.64 (d, J = 8.2 Hz, 24H), 8.46 (d. 48H), 8.42 (s. 24H), 8.41–8.38 (b, 48H), 8.37–8.28 (b, 96H), 8.24–8.23 (b, 72H), 8.19 (d, J = 8.2 Hz, 24H), 8.13–8.09 (b, 48H), 8.07–8.02 (b, 72H), 7.99 (d, J = 8.1 Hz, 24H), 7.94–7.87 (b, 72H), 7.65 (s, 12H), 7.64 (s, 12H), 7.60–7.57 (m, 24H), 7.52 (d, J = 7.7 Hz, 24H), 7.47–7.45 (m, 24H), 7.37–7.34 (m, 24H), 7.15 (d, J = 7.1 Hz, 24H), 6.89 (d, J = 7.1 Hz, 24H), 6.69 (t, J = 8.2 Hz, 24H), 6.51 (t, J = 8.2 Hz, 24H), 5.78 (d, J = 8.2 Hz, 48H), 5.61 (d, J = 8.2 Hz, 48H), 3.71–3.64 (m, 48H), 3.49–3.44 (m, 48H), 1.51 (s, 108H), 1.45–1.05 (m, 144H), 0.55 (dd, 288H), and 0.37 (s, 288H). ^{13}C NMR (125 MHz, CD_3CN): δ (ppm) 157.74, 156.82, 155.91, 155.40, 153.91, 153.26, 152.78, 151.57, 150.87, 150.69, 150.13, 149.28, 148.72, 143.98, 142.27, 141.62, 140.71, 137.32, 136.80, 135.86, 132.26, 131.80, 131.35, 129.68, 128.81, 128.30, 127.75, 124.77, 123.10, 122.70, 122.20, 121.35, 109.04, 72.02, 35.40, 31.69, 30.64, 29.94, 29.49, 26.55, 26.20, 22.65, and 21.15. ^{113}Cd NMR (111 MHz, CD_3CN): δ (ppm) 266.97 (6Cd), 239.38

(12Cd), and 236.96 (12Cd). ESI-MS (*m/z*): 2633.1700 [M − 16PF$_6$]$^{16+}$ (calcd *m/z* = 2633.1796), 2469.7876 [M − 17PF$_6$]$^{17+}$ (calcd *m/z* = 2469.7066), 2324.5003 [M − 18PF$_6$]$^{18+}$ (calcd *m/z* = 2324.5015), 2194.4744 [M − 19PF$_6$]$^{19+}$ (calcd *m/z* = 2194.5247), 2077.5497 [M − 20PF$_6$]$^{20+}$ (calcd *m/z* = 2077.5542), 1971.6730 [M − 21PF$_6$]$^{21+}$ (calcd *m/z* = 1971.6726), 1875.5344 [M − 22PF$_6$]$^{22+}$ (calcd *m/z* = 1875.5034), 1787.6377 [M − 23PF$_6$]$^{23+}$ (calcd *m/z* = 1787.6602), 1707.0869 [M − 24PF$_6$]$^{24+}$ (calcd *m/z* = 1707.0902), 1633.0560 [M − 25PF$_6$]$^{25+}$ (calcd *m/z* = 1633.0490), 1564.7639 [M − 26PF$_6$]$^{26+}$ (calcd *m/z* = 1564.6610), 1501.4026 [M − 27PF$_6$]$^{27+}$ (calcd *m/z* = 1501.3411), 1442.5719 [M − 28PF$_6$]$^{28+}$ (calcd *m/z* = 1442.5466), 1387.7179 [M − 29PF$_6$]$^{29+}$ (calcd *m/z* = 1387.7716), 1336.7562 [M − 30PF$_6$]$^{30+}$ (calcd *m/z* = 1336.7122), 1288.8981 [M − 31PF$_6$]$^{31+}$ (calcd *m/z* = 1288.8828), 1244.1021 [M − 32PF$_6$]$^{32+}$ (calcd *m/z* = 1244.1078), 1202.0234 [M − 33PF$_6$]$^{33+}$ (calcd *m/z* = 1201.9517), 1162.4874 [M − 34PF$_6$]$^{34+}$ (calcd *m/z* = 1162.4017), 1125.0791 [M − 35PF$_6$]$^{35+}$ (calcd *m/z* = 1125.0195), 1089.7837 [M − 36PF$_6$]$^{36+}$ (calcd *m/z* = 1089.7413), and 1056.4008 [M − 37PF$_6$]$^{37+}$ (calcd *m/z* = 1056.3992).

Synthesis of [Cd$_{30}$F^2$_8$V$_6$](PF$_6$)$_{60}$. By the general procedure, [Cd$_{30}$**F^2**$_8$**V**$_6$](PF$_6$)$_{60}$ was obtained in 96% yield (56.2 mg, 1.2 μmol) from **F^2** (23.2 mg, 10.3 μmol), **V** (19.6 mg, 7.8 μmol), Cd(NO$_3$)$_2$·4H$_2$O (12.6 mg, 40.7 μmol), and NH$_4$PF$_6$ (366.0 mg, 2.2 mmol). ^{1}H NMR (CD$_3$CN, 500 MHz): *δ* (ppm) 9.13 (s, 24H), 9.09 (s, 24H), 9.04 (s, 24H), 8.91 (d, *J* = 7.9 Hz, 24H), 8.76 (d, *J* = 8.3 Hz, 24H), 8.65 (d, *J* = 8.3 Hz, 24H), 8.47–8.37 (m, 144H), 8.30 (b, 48H), 8.24 (d, *J* = 4.2 Hz, 24H), 8.20 (d, *J* = 8.1 Hz, 24H), 8.11–7.92 (m, 240H), 7.85 (b, 24H), 7.65 (s, 12H), 7.64 (s, 12H), 7.59–7.58 (b, 24H), 7.52 (d, *J* = 7.8 Hz, 24H), 7.46 (t, *J* = 6.0 Hz, 24H), 7.34–7.33 (b, 24H), 7.15 (d, *J* = 7.4 Hz, 24H), 6.87 (d, *J* = 7.2 Hz, 24H), 6.68 (t, *J* = 8.3 Hz, 24H), 6.48–6.47 (b, 24H), 5.78 (d, *J* = 8.5 Hz, 48H), 5.59 (d, *J* = 8.3 Hz, 48H), 3.70–3.63 (m, 72H), 3.47–3.45 (m, 72H), 3.35 (s, 36H), 3.25 (s, 36H), 1.51 (s, 108H), 1.41–1.09 (m, 144H), 0.57 (d, *J* = 5.8 Hz, 144H), 0.52 (d, *J* = 6.0 Hz, 144H), and 0.35 (d, *J* = 5.6 Hz, 288H). ^{13}C NMR (CD$_3$CN, 125 MHz): *δ* (ppm) 157.71, 157.53, 156.72, 156.47, 155.93, 155.49, 155.31, 153.81, 153.22, 152.79, 152.67, 151.58, 151.50, 150.84, 150.13, 150.07, 149.19, 149.08, 148.64, 143.16, 142.81, 142.22, 141.51, 140.63, 140.47, 138.39, 137.23, 136.90, 136.43, 136.00, 135.72, 135.25, 132.84, 132.50, 131.87, 131.70, 131.47, 131.22, 130.91, 128.65, 128.25, 127.70, 126.46, 124.80, 124.39, 122.90, 122.50, 122.11, 121.20, 120.96, 108.94, 77.04, 74.60, 71.91, 71.69, 61.69, 61.54, 35.36, 31.64, 30.75, 30.44, 30.19, 29.87, 26.70, 26.46, 22.57, 22.46, 21.09, and 20.95. ^{113}Cd (CD$_3$CN, 111 MHz): *δ* (ppm) 267.05 (6Cd), 239.55 (12Cd), and 237.33 (12Cd). ESI-MS (*m/z*): 3619.4133 [M − 12PF$_6$]$^{12+}$ (calcd *m/z* =

3619.2517), 3329.7988 [M − 13PF$_6$]$^{13+}$ (calcd m/z = 3329.6982), 3081.7615 [M − 14PF$_6$]$^{14+}$ (calcd m/z = 3081.5042), 2866.5522 [M − 15PF$_6$]$^{15+}$ (calcd m/z = 2866.4053), 2678.3374 [M − 16PF$_6$]$^{16+}$ (calcd m/z = 2678.1956), 2512.4036 [M − 17PF$_6$]$^{17+}$ (calcd m/z = 2512.1279), 2364.7959 [M − 18PF$_6$]$^{18+}$ (calcd m/z = 2364.5156), 2232.5752 [M − 19PF$_6$]$^{19+}$ (calcd m/z = 2232.4329), 2113.6985 [M − 20PF$_6$]$^{20+}$ (calcd m/z = 2113.5669), 2006.2152 [M − 21PF$_6$]$^{21+}$ (calcd m/z = 2006.0173), 1908.4277 [M − 22PF$_6$]$^{22+}$ (calcd m/z = 1908.2422), 1819.1971 [M − 23PF$_6$]$^{23+}$ (calcd m/z = 1818.9756), 1737.3308 [M − 24PF$_6$]$^{24+}$ (calcd m/z = 1737.1398), 1661.9641 [M − 25PF$_6$]$^{25+}$ (calcd m/z = 1661.8591), 1592.4720 [M − 26PF$_6$]$^{26+}$ (calcd m/z = 1592.3632), 1528.1892 [M − 27PF$_6$]$^{27+}$ (calcd m/z = 1528.0171), 1468.3798 [M − 28PF$_6$]$^{28+}$ (calcd m/z = 1468.2700), 1412.7711 [M − 29PF$_6$]$^{29+}$ (calcd m/z = 1412.6392), 1360.9288 [M − 30PF$_6$]$^{30+}$ (calcd m/z = 1360.7206), 1312.2572 [M − 31PF$_6$]$^{31+}$ (calcd m/z = 1312.1481), and 1266.6499 [M − 32PF$_6$]$^{32+}$ (calcd m/z = 1266.6157).

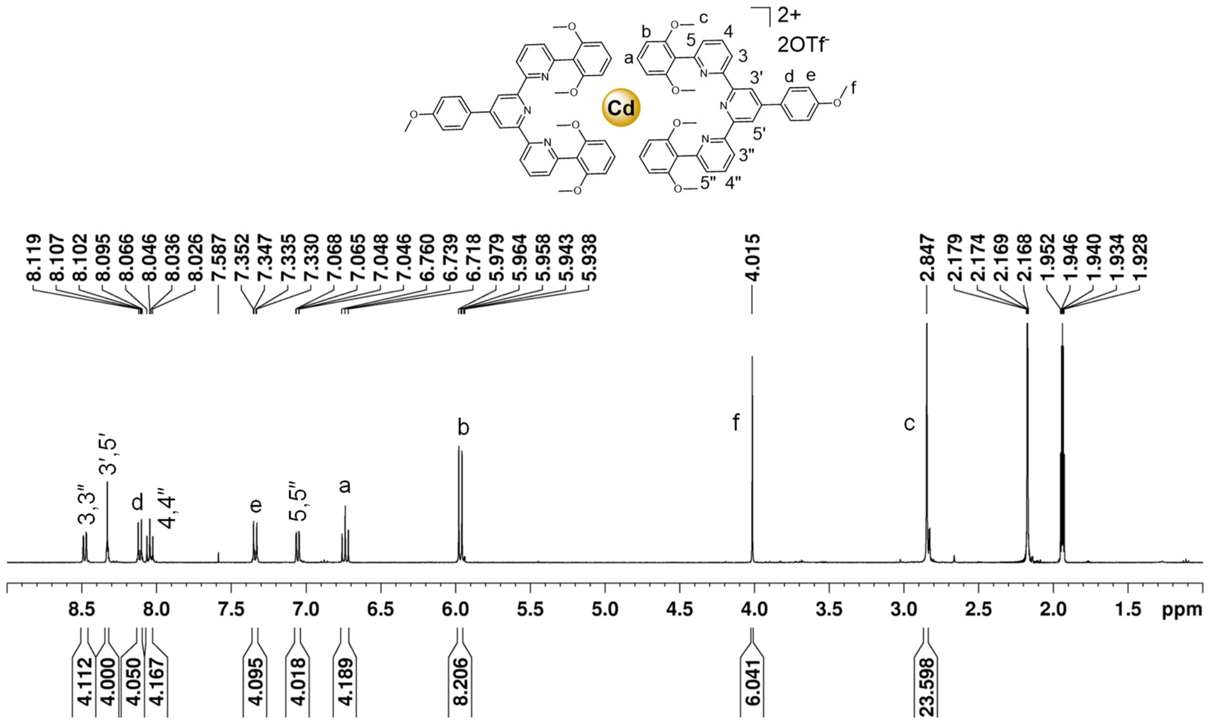

Figure S21. ^{1}H NMR spectrum (400 MHz, CD_3CN) of [Cd**L**$^{\mathbf{a}}$$_2$].

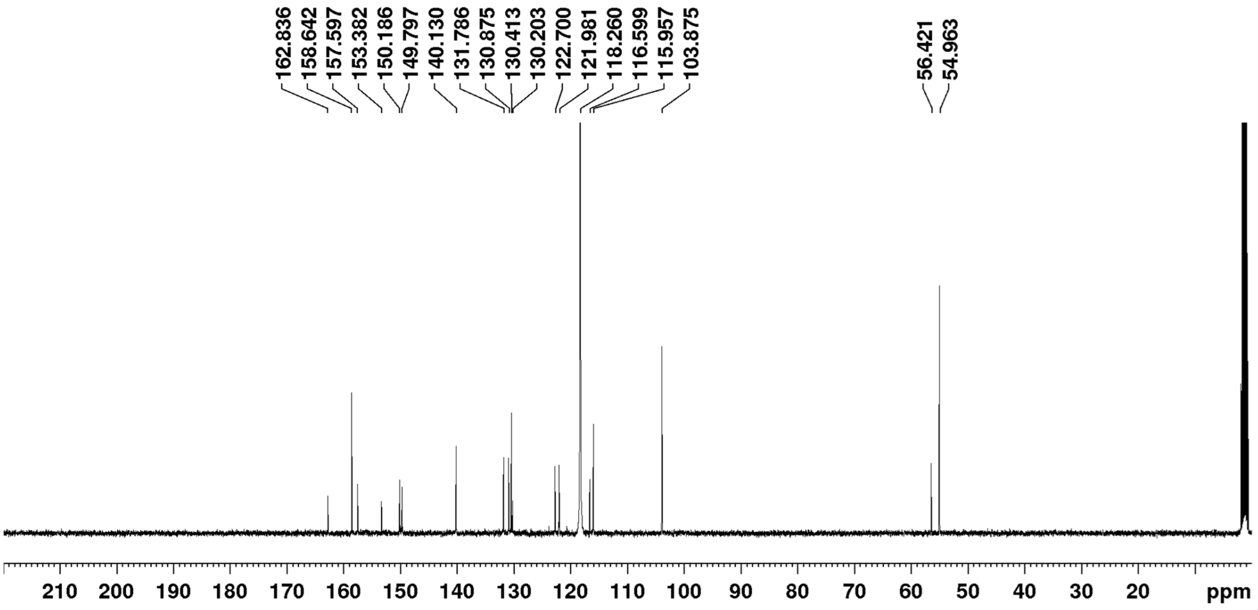

Figure S22. ^{13}C NMR spectrum (100 MHz, CD_3CN) of [Cd$\mathbf{L}^a{}_2$].

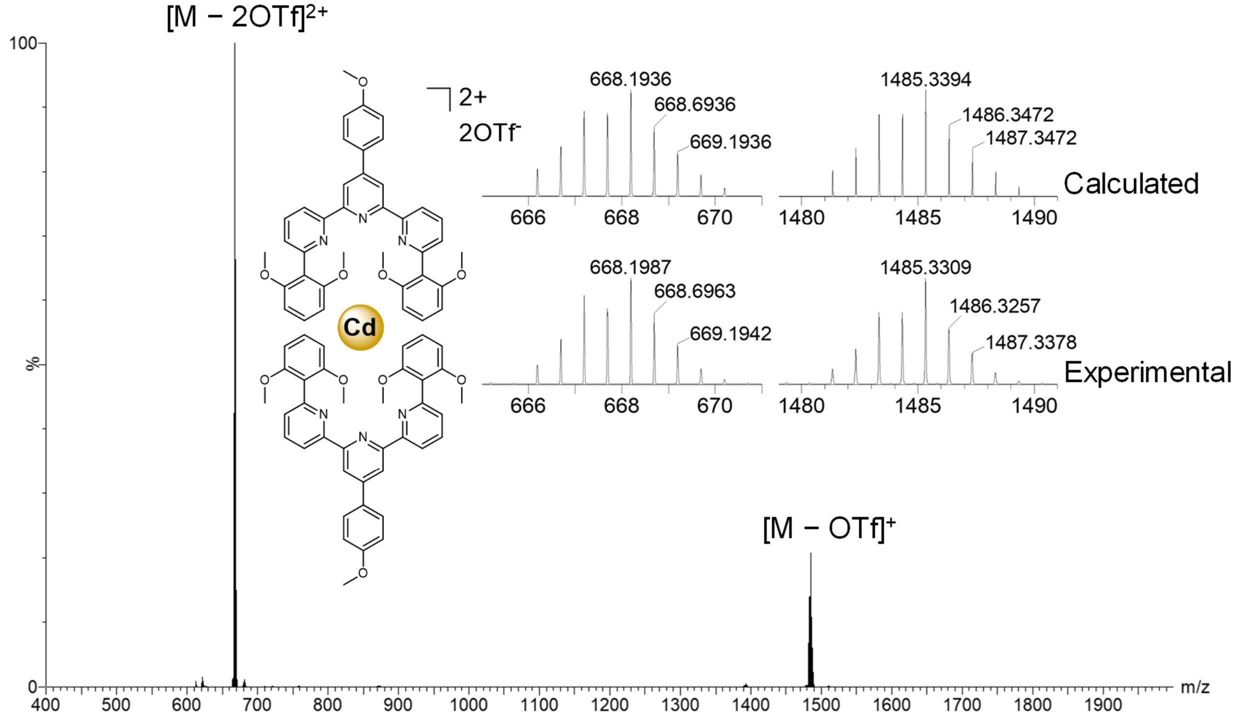

Figure S23. ESI-MS spectrum of [Cd$\mathbf{L}^a{}_2$]$(OTf)_2$ and the corresponding isotope patterns.

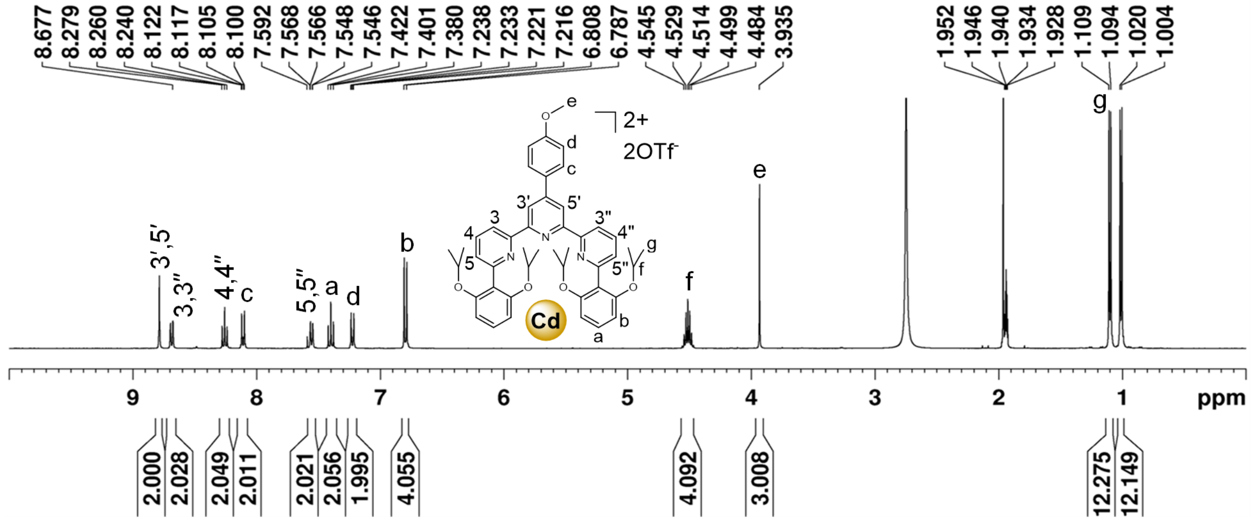

Figure S24. ^{1}H NMR spectrum (400 MHz, CD_3CN) of [Cd**L^b^**]$(OTf)_2$.

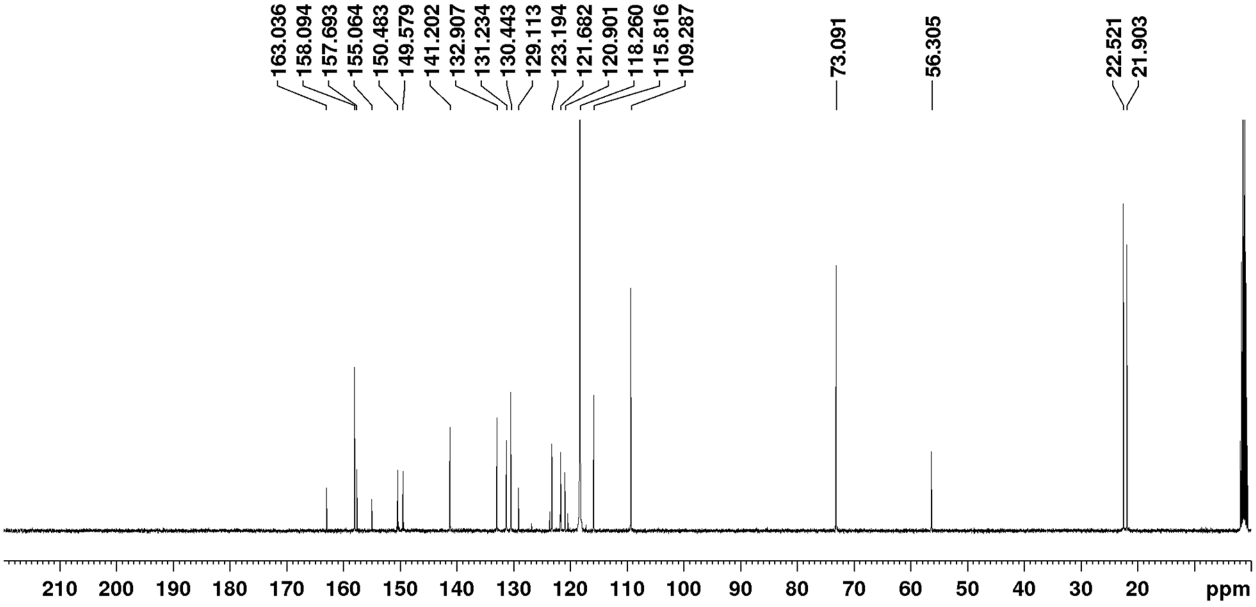

Figure S25. ^{13}C NMR spectrum (100 MHz, CD_3CN) of [Cd**L^b^**]$(OTf)_2$.

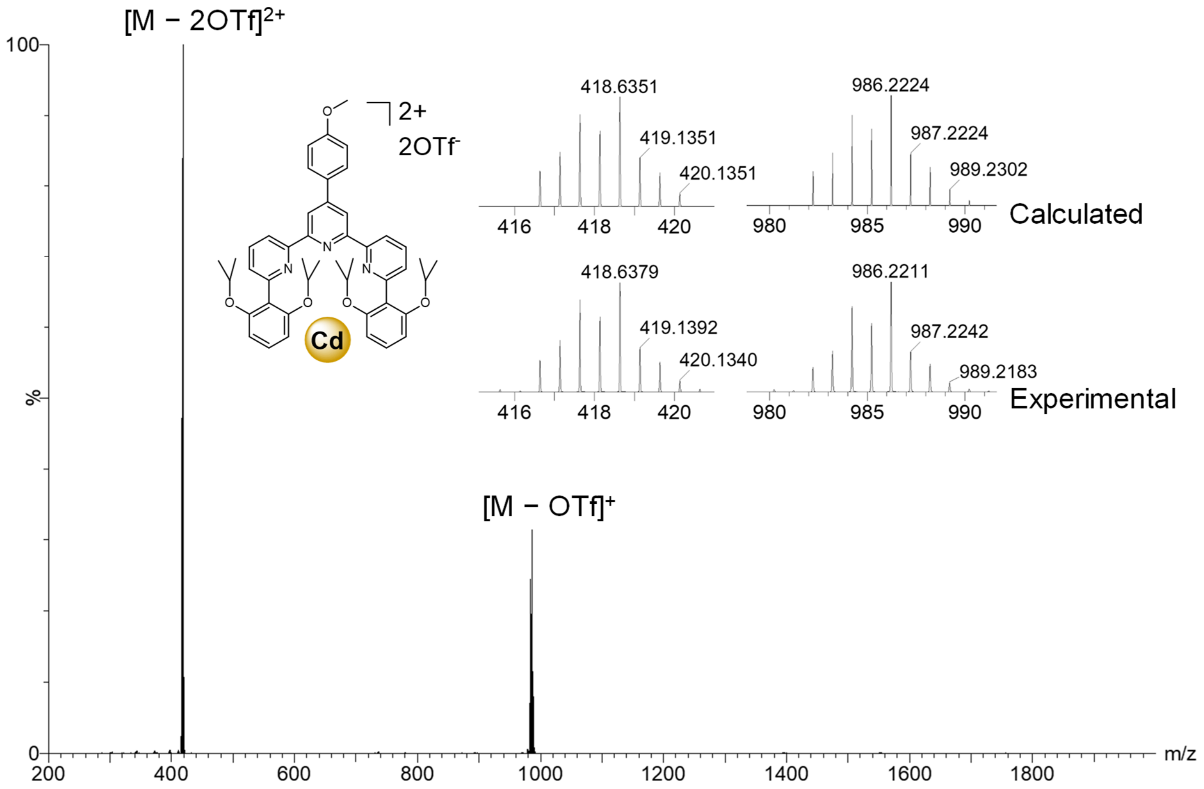

Figure S26. ESI-MS spectrum of [Cd**L**$^{\mathbf{b}}$](OTf)$_2$ and the corresponding isotope patterns.

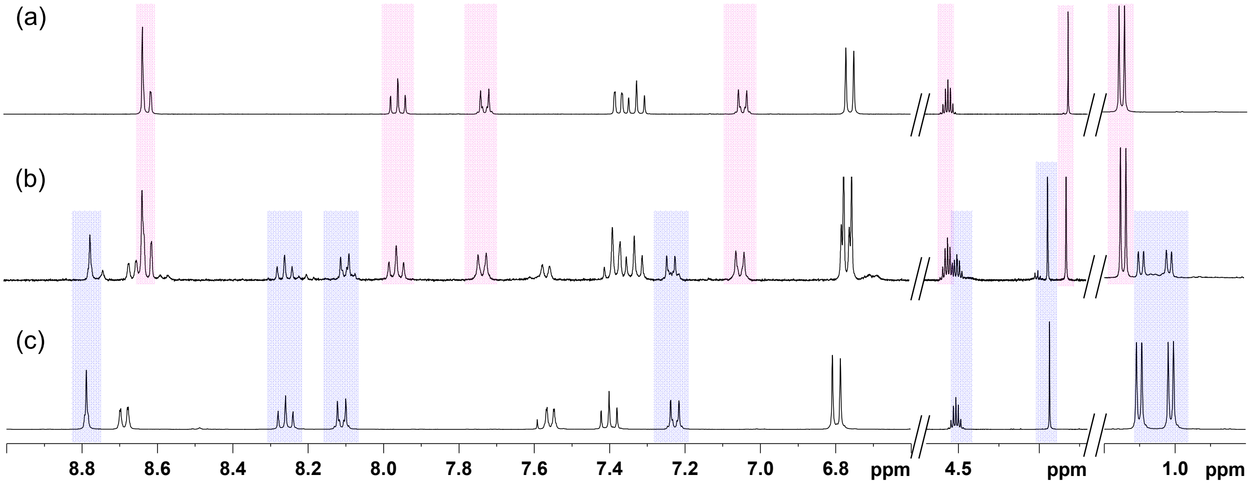

Figure S27. ^{1}H NMR spectra (400 MHz, CD_3CN) of (a) ligand **L**$^{\mathbf{b}}$, (b) a 2:1 mixture of ligand **L**$^{\mathbf{b}}$ and Cd(OTf)$_2$ heated at 80 °C for 3 days, and (c) [Cd**L**$^{\mathbf{b}}$].

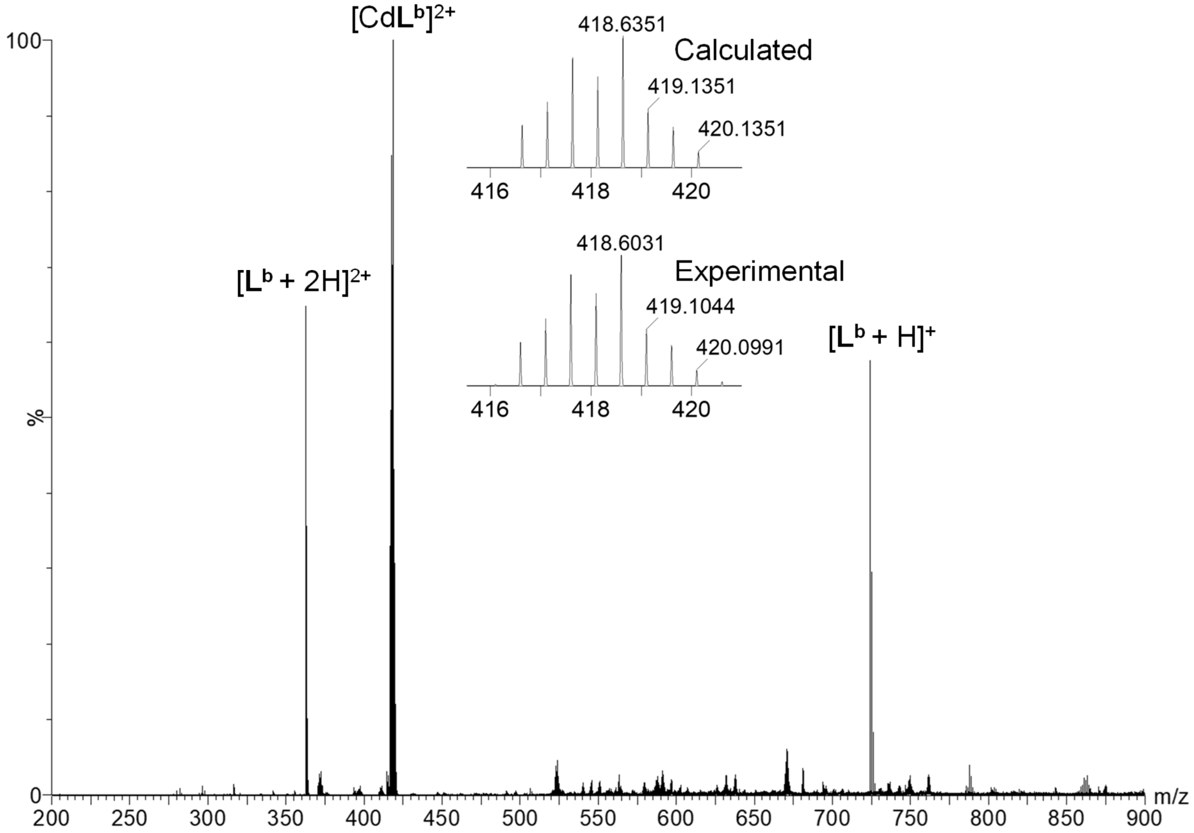

Figure S28. ESI-MS spectrum of a 2:1 mixture of ligand $\mathbf{L^b}$ and $Cd(OTf)_2$ after heating at 80 °C for 3 days and the isotope pattern of $[Cd\mathbf{L^b}]^{2+}$.

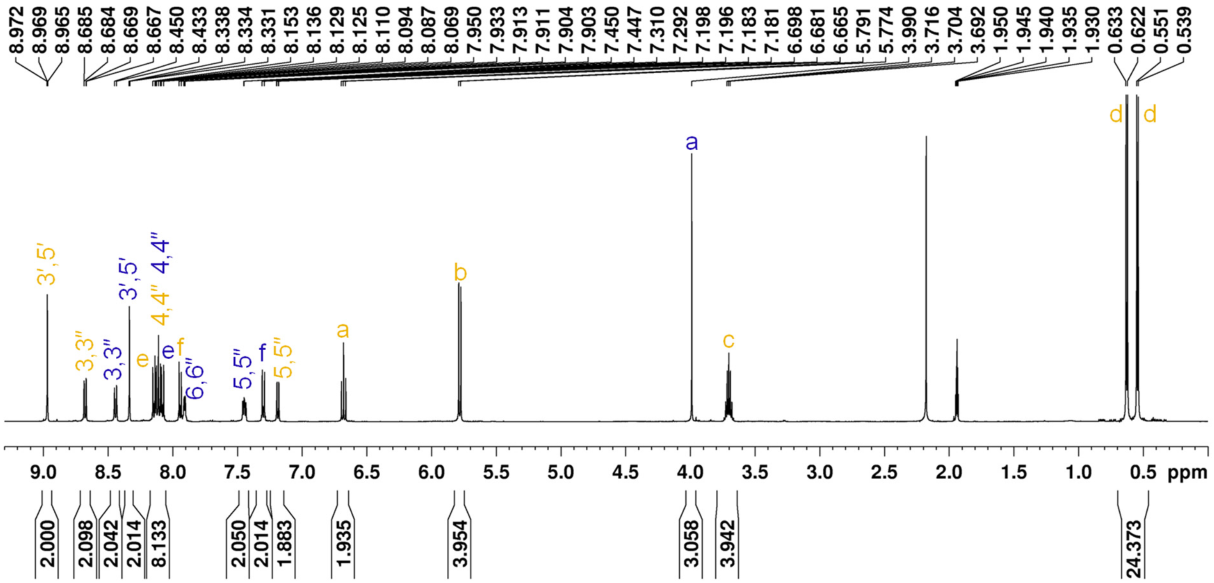

Figure S29. ^1H NMR spectrum (500 MHz, CD_3CN) of $[Cd\mathbf{L^c}\mathbf{L^d}]$.

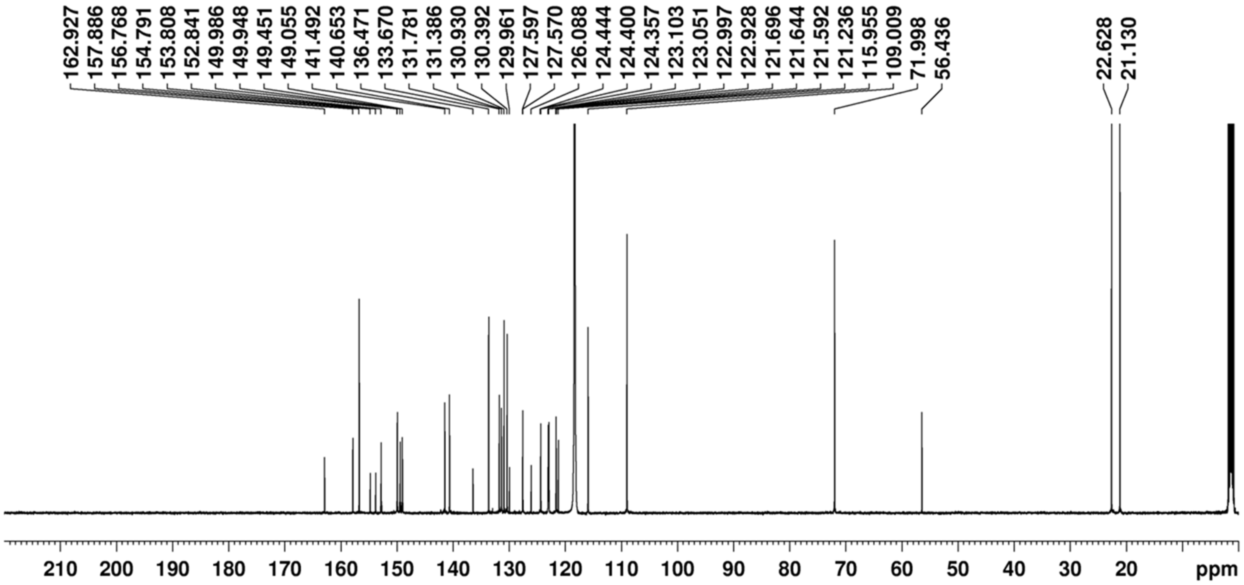

Figure S30. ^{13}C NMR spectrum (125 MHz, CD_3CN) of [Cd**L**^{c}**L**^{d}].

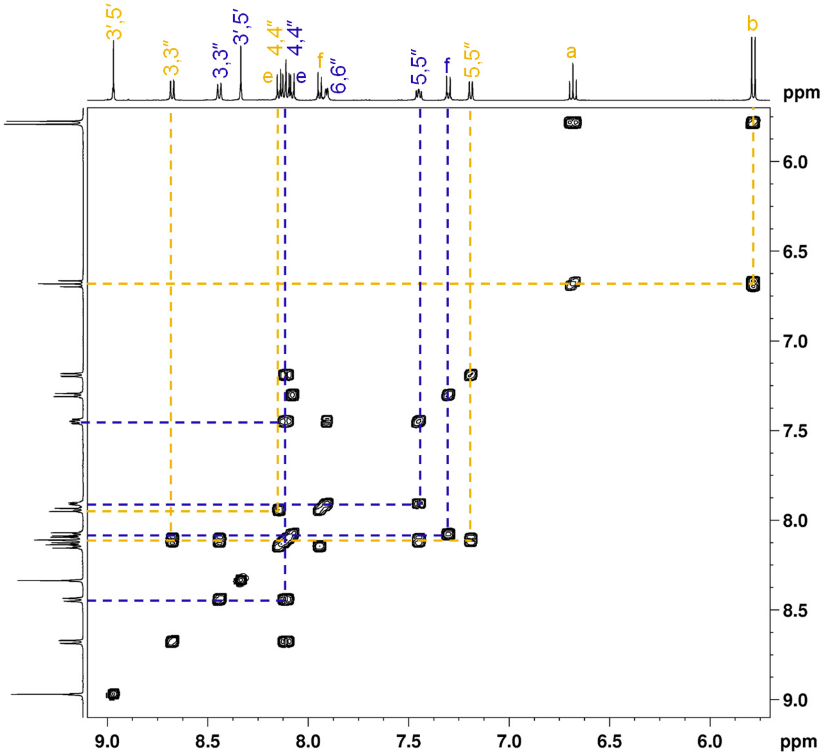

Figure S31. Partial COSY spectrum (500 MHz, CD_3CN) of [Cd**L**^{c}**L**^{d}].

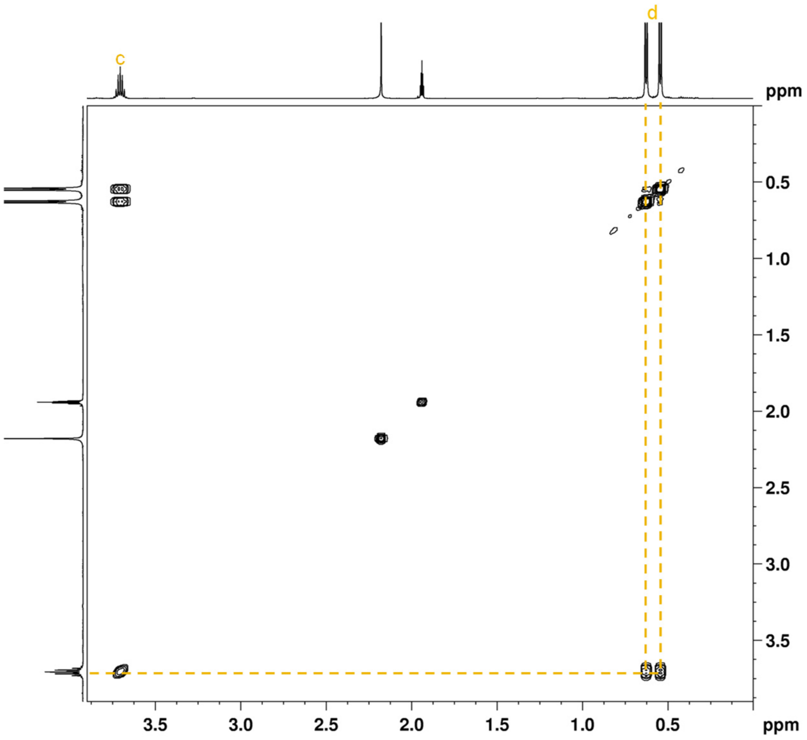

Figure S32. Partial COSY spectrum (500 MHz, CD_3CN) of [Cd$\mathbf{L^cL^d}$].

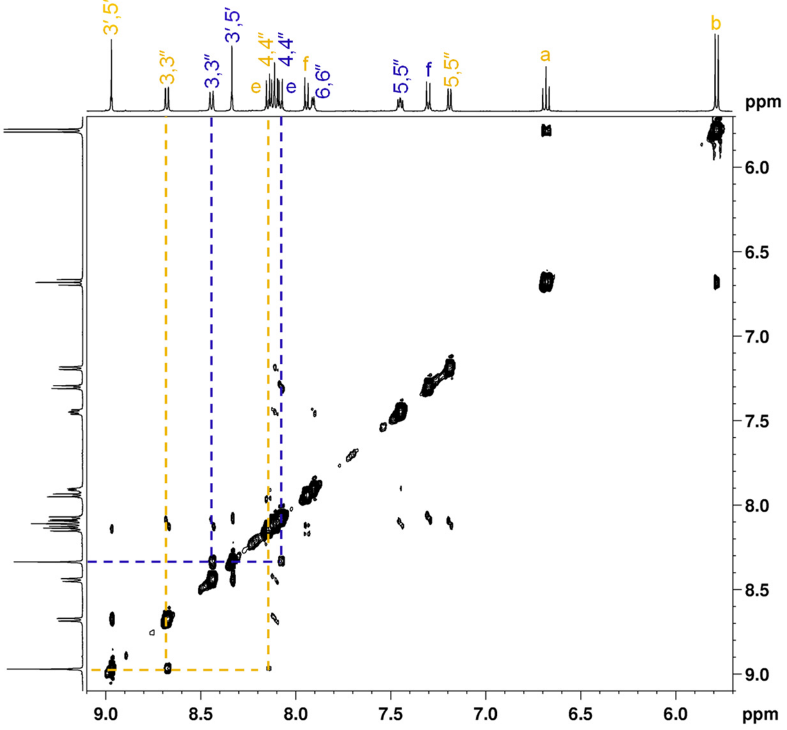

Figure S33. Partial ROESY spectrum (500 MHz, CD_3CN) of [Cd$\mathbf{L^cL^d}$].

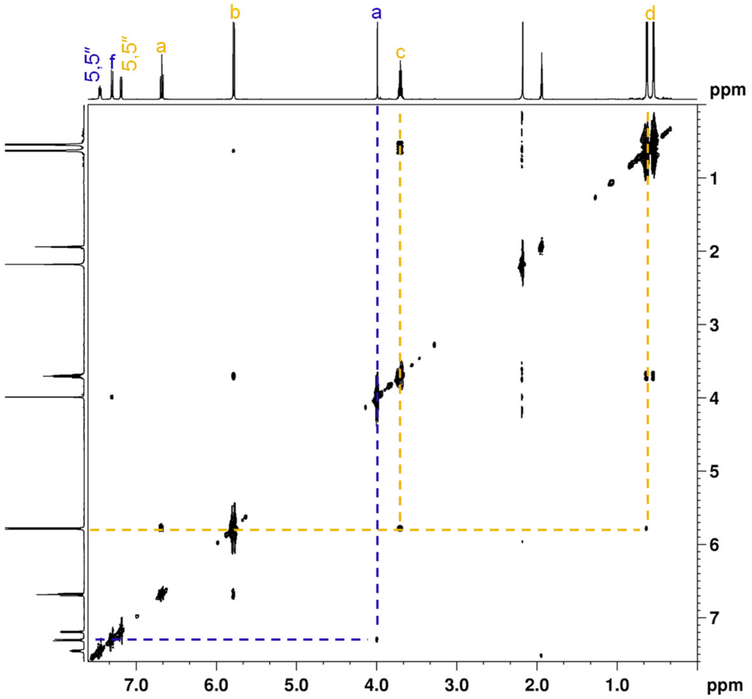

Figure S34. Partial ROESY spectrum (500 MHz, CD_3CN) of [Cd**L**^{c}**L**^{d}].

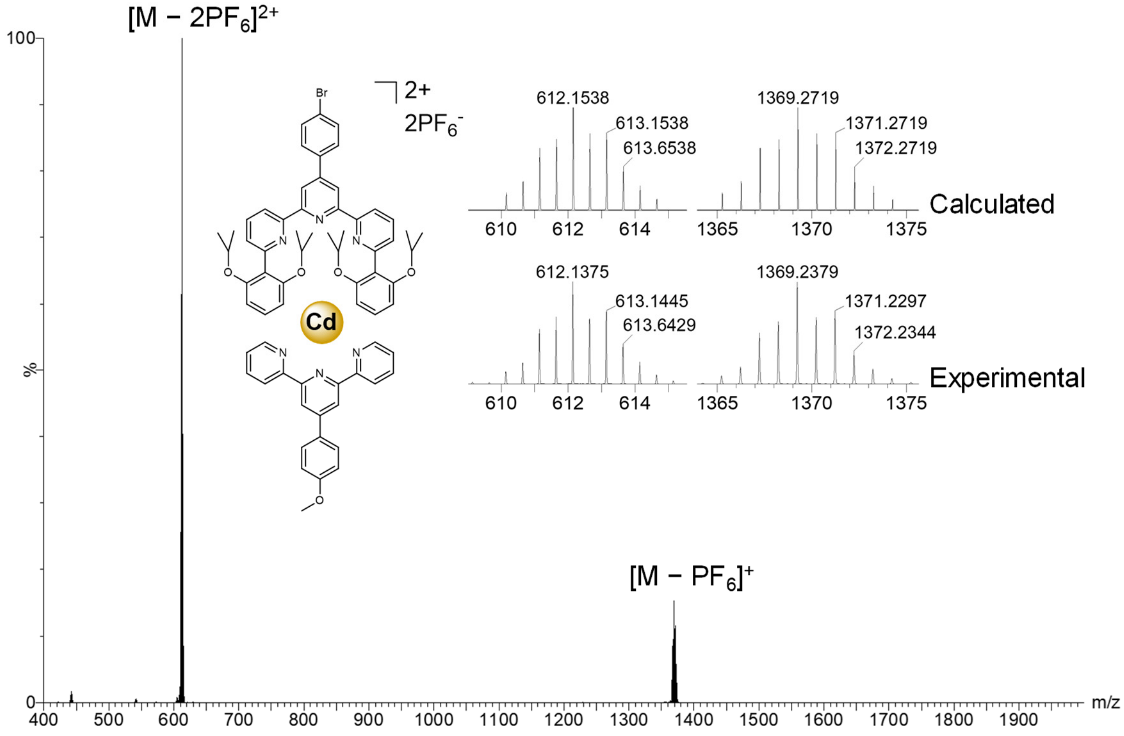

Figure S35. ESI-MS spectrum of [Cd**L**^{c}**L**^{d}] and the corresponding isotope patterns.

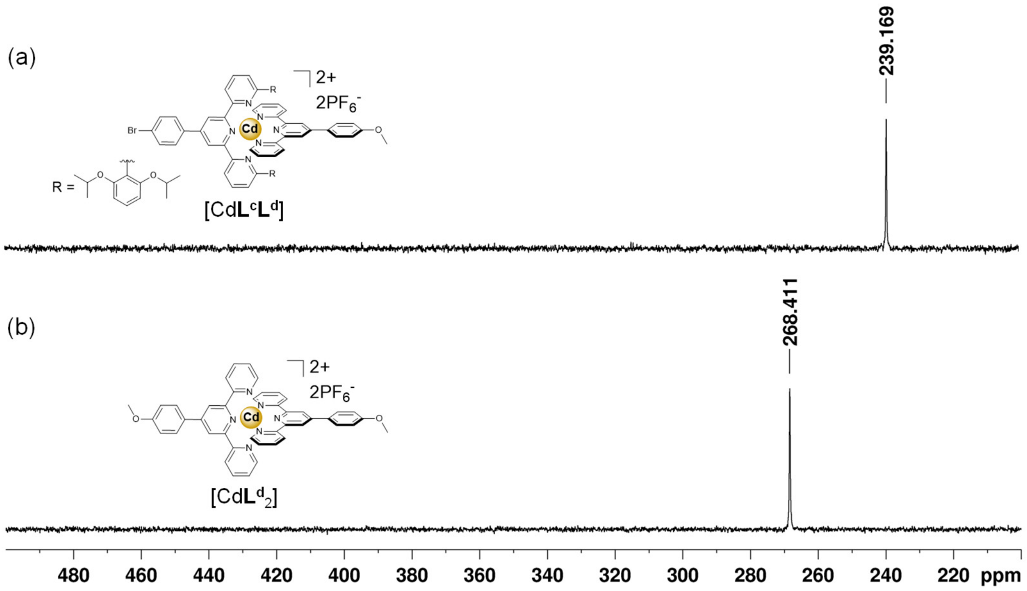

Figure S36. ^{113}Cd NMR spectra (111 MHz, CD_3CN) of (a) [Cd**L**^{c}**L**^{d}] and (b) [Cd**L**$^d{}_2$] in CD_3CN.

Isothermal Titration Calorimetry Experiments. All isothermal titrations were conducted using a low volume Affinity ITC (TA Instruments) at 298 K. The data fitting was executed by using the NanoAnalyze program from TA instruments. The binding constants of complex [Cd**L**^{b}**L**^{d}] in MeCN were determined by ITC experiments. The first binding constant K_1 ($4.18 \pm 0.56 \times 10^7$ M^{-1}) was obtained by titrating $Cd(NTf_2)_2$ (0.27 mM) with **L**^{b} (2.50 mM). The second binding constant K_2 ($3.45 \pm 0.44 \times 10^7$ M^{-1}) was obtained by titrating [Cd**L**^{b}]$(NTf_2)_2$ (0.22 mM) with **L**^{d} (2.50 mM). The data were corrected by subtracting heat-of-dilution measurements, and the fitting curve was generated by independent (one-site binding) model.

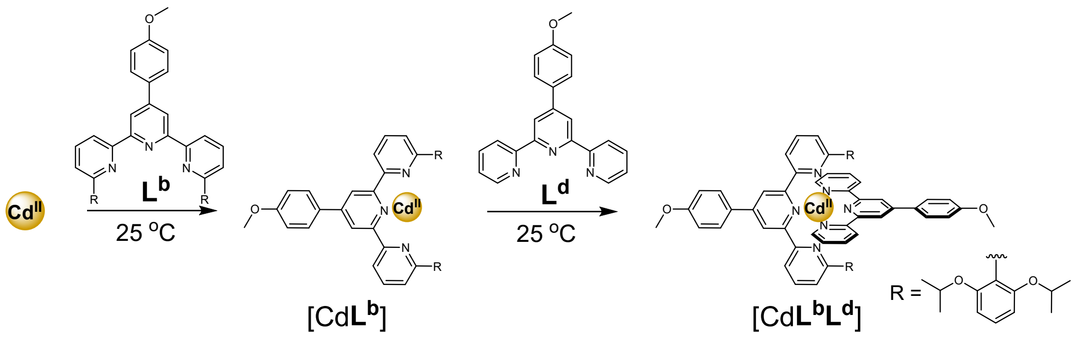

Scheme S2. Titration experiments for binding constant measurements.

$$\mathbf{L^b} + \mathrm{Cd(NTf_2)_2} \rightleftharpoons [\mathrm{Cd}\mathbf{L^b}]\mathrm{(NTf_2)_2} \quad K_1 = \frac{[[\mathrm{Cd}\mathbf{L^b}](\mathrm{NTf_2})_2]}{[\mathbf{L^b}][\mathrm{Cd(NTf_2)_2}]}$$

$$\mathbf{L^d} + [\mathrm{Cd}\mathbf{L^b}]\mathrm{(NTf_2)_2} \rightleftharpoons [\mathrm{Cd}\mathbf{L^b}\mathbf{L^d}]\mathrm{(NTf_2)_2} \quad K_2 = \frac{[[\mathrm{Cd}\mathbf{L^b}\mathbf{L^d}](\mathrm{NTf_2})_2]}{[\mathbf{L^d}][[\mathrm{Cd}\mathbf{L^b}](\mathrm{NTf_2})_2]}$$

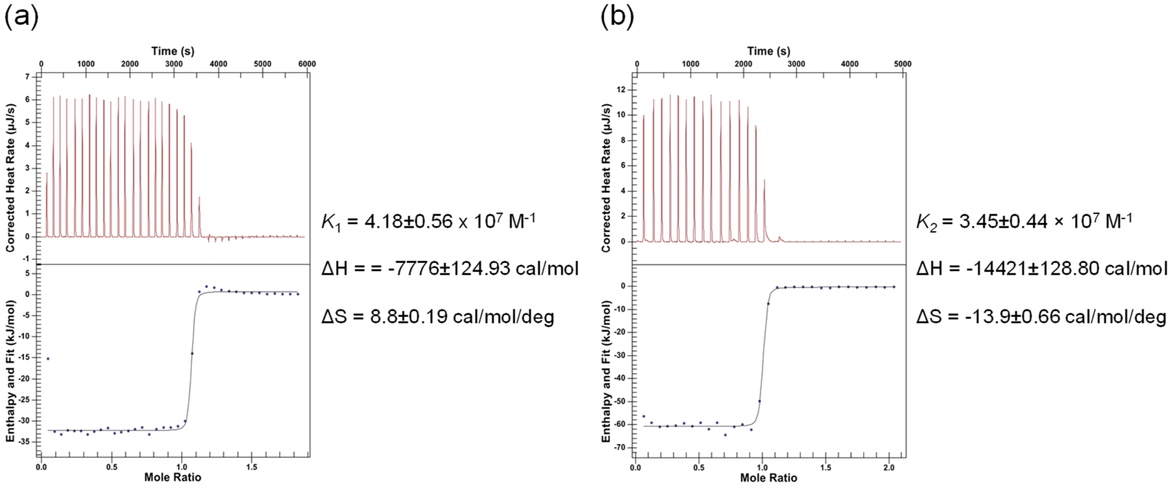

Figure S37. ITC results for formation of (a) $[\mathrm{Cd}\mathbf{L^b}]\mathrm{(NTf_2)_2}$ and (b) $[\mathrm{Cd}\mathbf{L^b}\mathbf{L^d}]\mathrm{(NTf_2)_2}$.

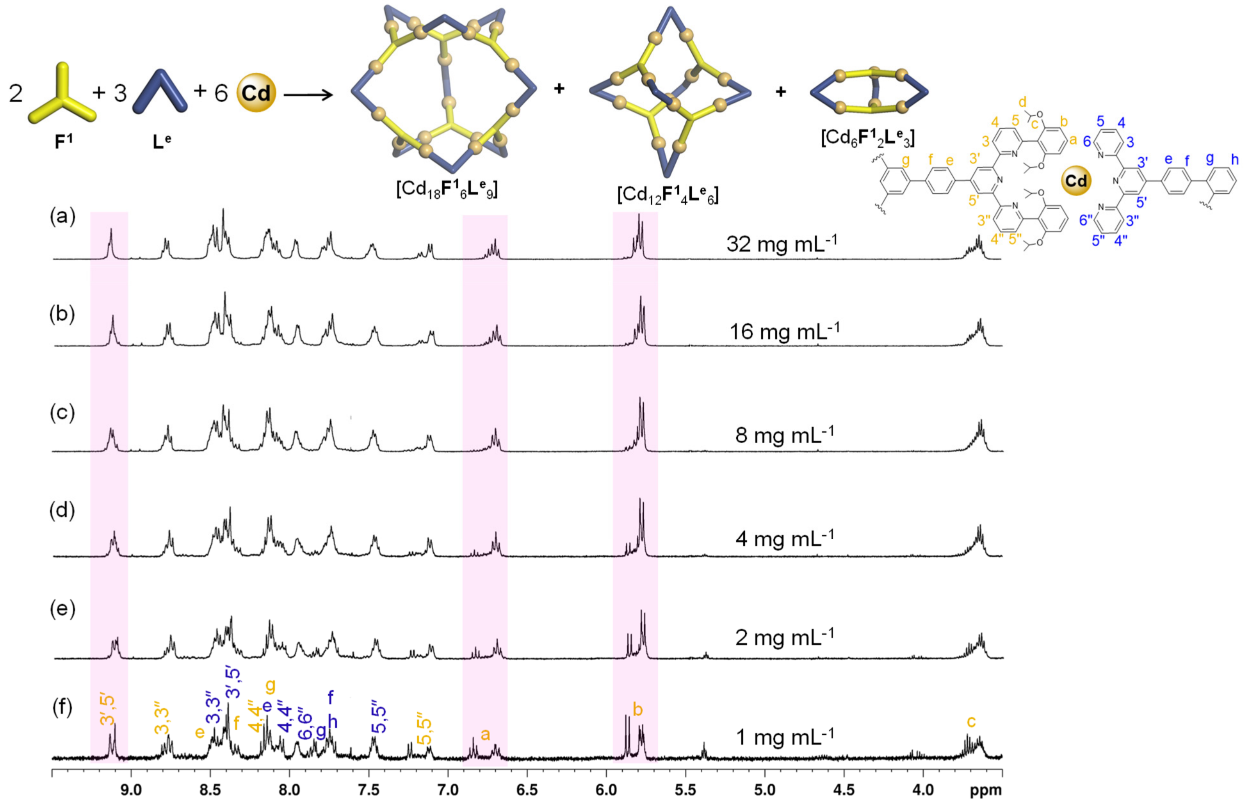

Figure S38. ^{1}H NMR spectra (400 MHz, CD_3CN) of a mixture of complexes $[\mathrm{Cd_{18}}\mathbf{F^1}_6\mathbf{L^e}_9]$, $[\mathrm{Cd_{12}}\mathbf{F^1}_4\mathbf{L^e}_6]$, and $[\mathrm{Cd_6}\mathbf{F^1}_2\mathbf{L^e}_3]$ at different concentrations: (a) 32, (b) 16, (c) 8, (d) 4, (e) 2, and (f) 1 mg mL^{-1}.

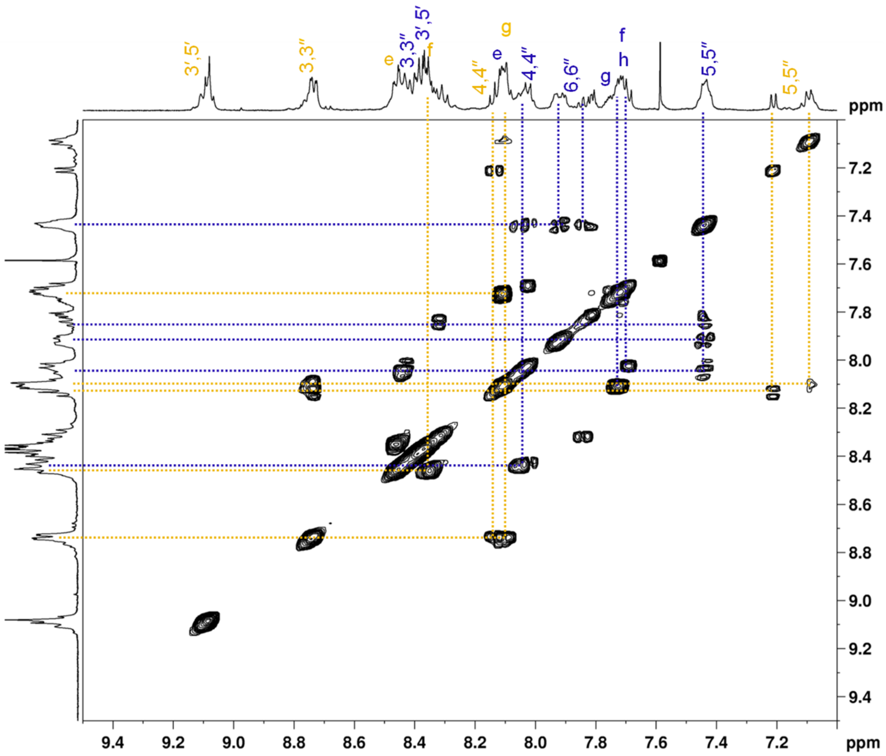

Figure S39. Partial COSY spectrum (500 MHz, CD_3CN) of a mixture of $[Cd_{6n}\mathbf{F}^{1}_{2n}\mathbf{L}^{e}_{3n}]$.

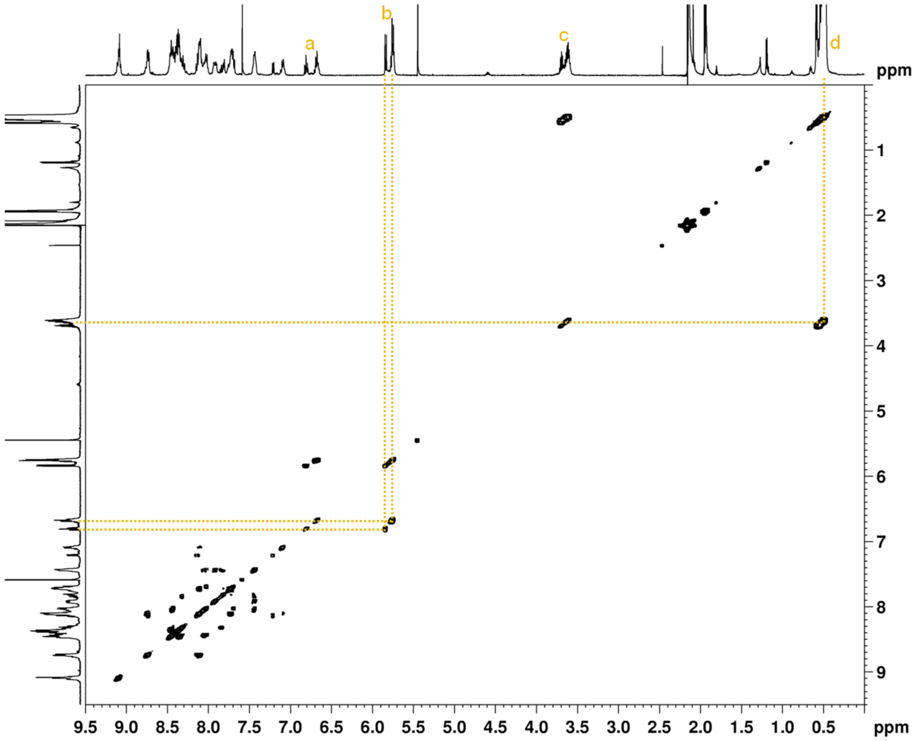

Figure S40. Partial COSY spectrum (500 MHz, CD_3CN) of a mixture of $[Cd_{6n}\mathbf{F}^{1}_{2n}\mathbf{L}^{e}_{3n}]$.

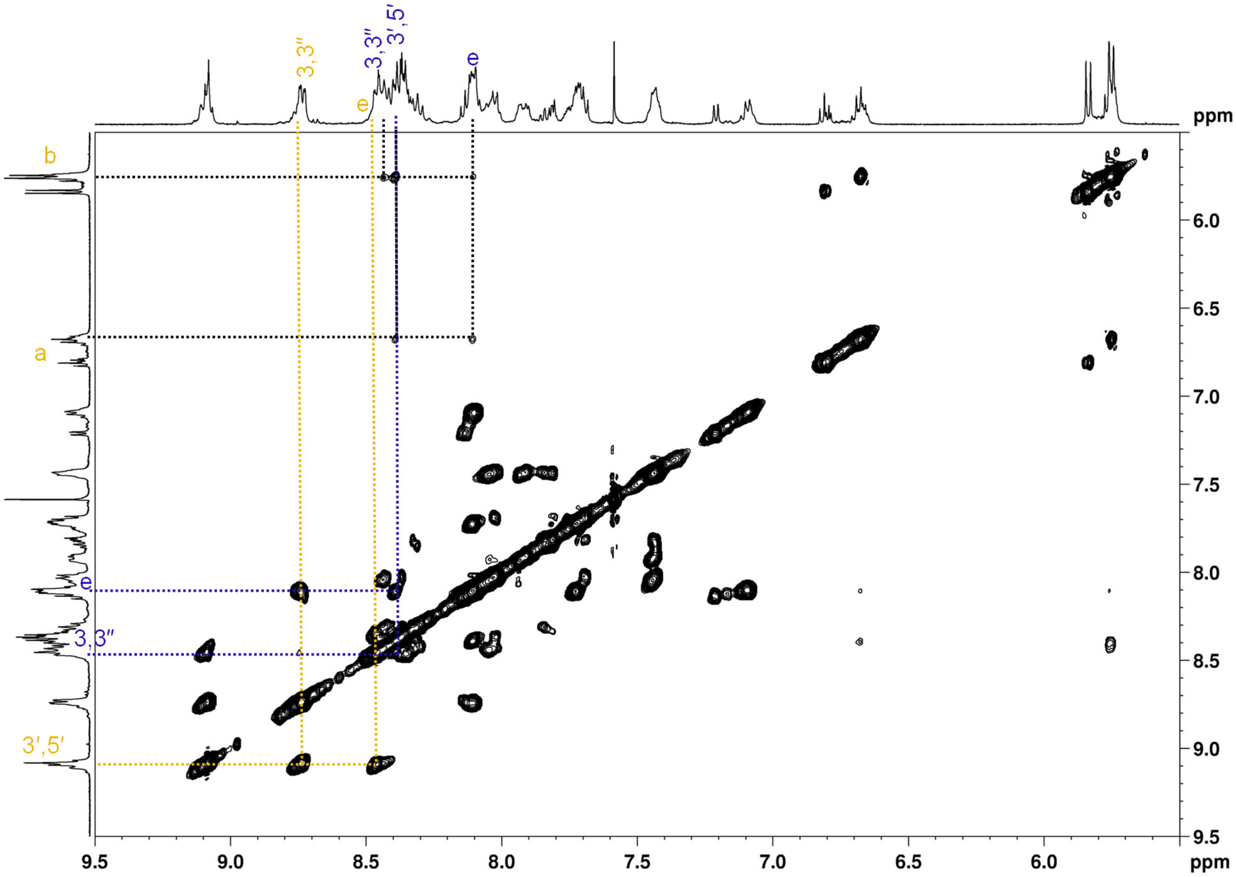

Figure S41. Partial ROESY spectrum (500 MHz, CD_3CN) of a mixture of [$Cd_{6n}\mathbf{F}^1{}_{2n}\mathbf{L}^e{}_{3n}$].

DOSY NMR Experiments. The diffusion coefficients were calculated using an internal reference value of 2.18×10^{-9} m^2s^{-1} for the CHD_2CN signal in CD_3CN at 298 K.[14] The hydrodynamic radii (r_H) were subsequently calculated via the Stokes–Einstein equation[15] and compared with the simulated molecular dimensions derived from geometry-optimized models (Figure S43). For each complex, the experimentally derived r_H values show good agreement with the theoretically estimated cage sizes. The calculated radii (r_c) were estimated from geometry-optimized structures as follows: for the [$Cd_{30}\mathbf{F}^1{}_8\mathbf{V}_6$] and [$Cd_{12}\mathbf{F}^1{}_4\mathbf{L}^e{}_6$], r_c was defined as half of the maximum molecular dimension; for [$Cd_{18}\mathbf{F}^1{}_6\mathbf{L}^e{}_9$] and [$Cd_6\mathbf{F}^1{}_2\mathbf{L}^e{}_3$], r_c was approximated by averaging the height and width/diameter dimensions, i.e., r_c = (height + width/diameter) / 4.

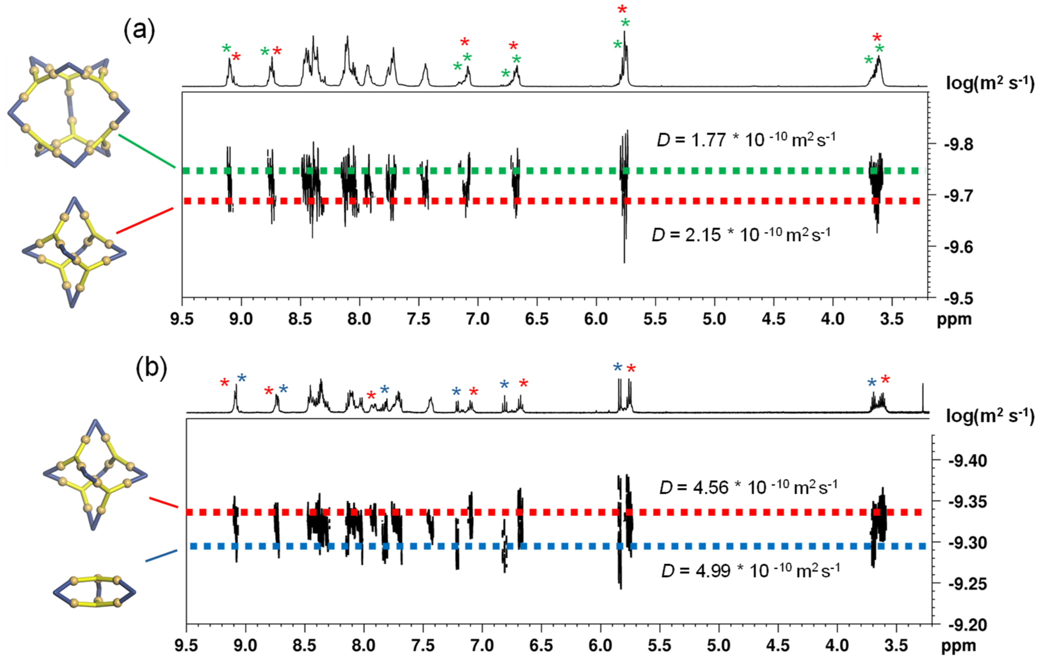

Figure S42. DOSY spectra (500 MHz, CD_3CN) of (a) a mixture of [$Cd_{18}\mathbf{F^1}_6\mathbf{L^e}_9$] and [$Cd_{12}\mathbf{F^1}_4\mathbf{L^e}_6$] (10 mg mL^{-1}) and (b) a mixture of [$Cd_{12}\mathbf{F^1}_4\mathbf{L^e}_6$] and [$Cd_6\mathbf{F^1}_2\mathbf{L^e}_3$] (1.5 mg mL^{-1}). Dotted green, red, and blue lines correspond to [$Cd_{18}\mathbf{F^1}_6\mathbf{L^e}_9$], [$Cd_{12}\mathbf{F^1}_4\mathbf{L^e}_6$], and [$Cd_6\mathbf{F^1}_2\mathbf{L^e}_3$], respectively.

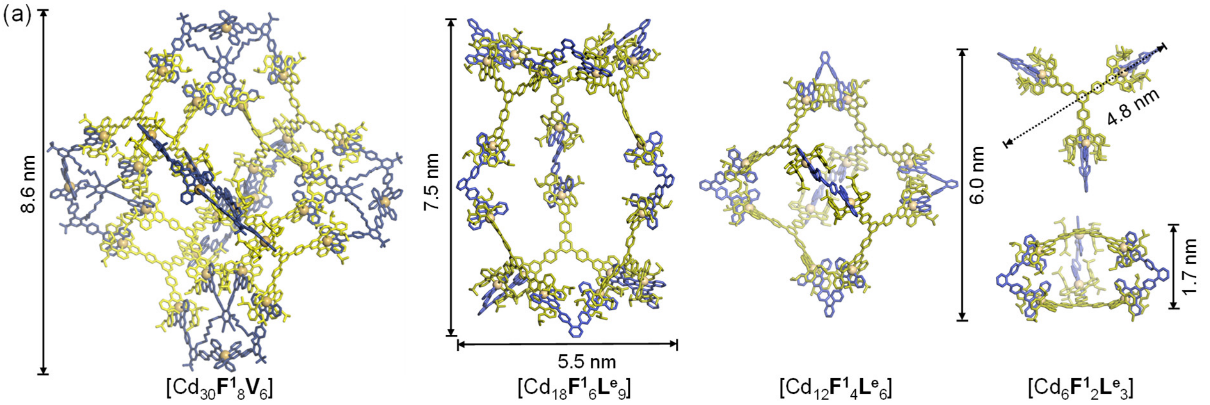

(b)

Complex	D [10^{-10} m^{2}s^{-1}][a]	r_H (exp) [nm][b]	r_c (calcd) [nm][c]
[$Cd_{30}\mathbf{F^1}_8\mathbf{V}_6$]	1.50	3.96	4.30
[$Cd_{18}\mathbf{F^1}_6\mathbf{L^e}_9$]	1.77	3.36	3.25
[$Cd_{12}\mathbf{F^1}_4\mathbf{L^e}_6$]	2.15	2.77	3.00
[$Cd_6\mathbf{F^1}_2\mathbf{L^e}_3$]	4.99	1.19	1.63

Figure S43. (a) Geometry-optimized structures with corresponding dimensions, and (b) diffusion coefficients, hydrodynamic radii, and calculated molecular radii of [$Cd_{30}\mathbf{F^1}_8\mathbf{V}_6$], [$Cd_{18}\mathbf{F^1}_6\mathbf{L^e}_9$], [$Cd_{12}\mathbf{F^1}_4\mathbf{L^e}_6$], and [$Cd_6\mathbf{F^1}_2\mathbf{L^e}_3$]. [a]Diffusion coefficients determined by 2D DOSY NMR experiments in CD_3CN at 298 K. [b]Hydrodynamic radii calculated using the Stokes-Einstein equation: $D = k_B T / 6\pi\eta r_H$ (k_B, Boltzmann constant; T, absolute temperature; η = 0.367 mPa·s, viscosity of CD_3CN at 298 K). [c]Molecular radii estimated from the geometry-optimized structures.

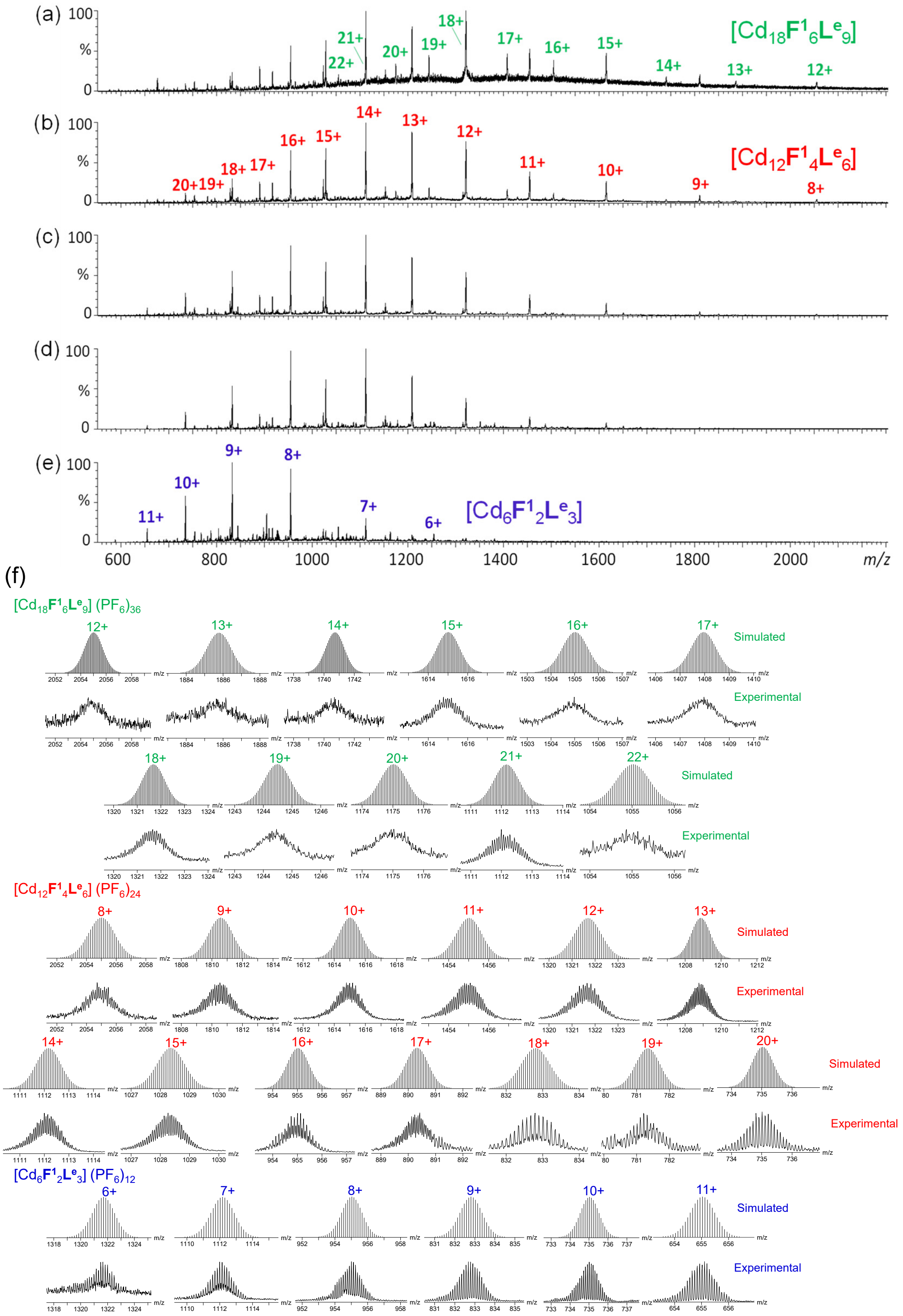

Figure S44. ESI-MS spectra of a mixture of $[Cd_{6n}\mathbf{F}^{1}_{2n}\mathbf{L}^{e}_{3n}]$ at different concentrations in MeCN: (a) 16, (b) 8, (c) 4, (d) 2, and (e) 1 mg mL^{-1}. Signals colored in green, red, and blue correspond to $[Cd_{18}\mathbf{F}^{1}_{6}\mathbf{L}^{e}_{9}]$, $[Cd_{12}\mathbf{F}^{1}_{4}\mathbf{L}^{e}_{6}]$, and $[Cd_{6}\mathbf{F}^{1}_{2}\mathbf{L}^{e}_{3}]$, respectively. (f) The corresponding experimental and simulated isotope patterns.

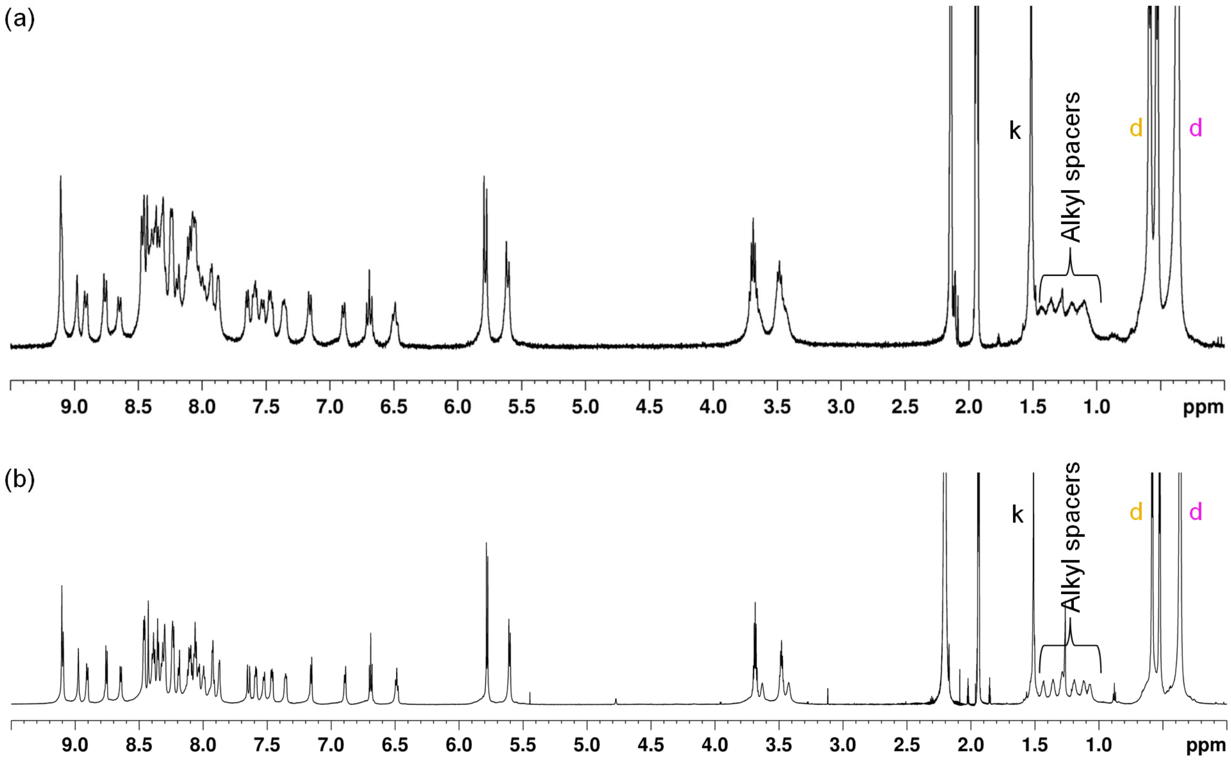

Figure S45. ^{1}H NMR spectra recorded on (a) 400 MHz and (b) 800 MHz spectrometers in CD_3CN for [Cd_{30}**F**$^{1}{}_{8}$**V**$_{6}$].

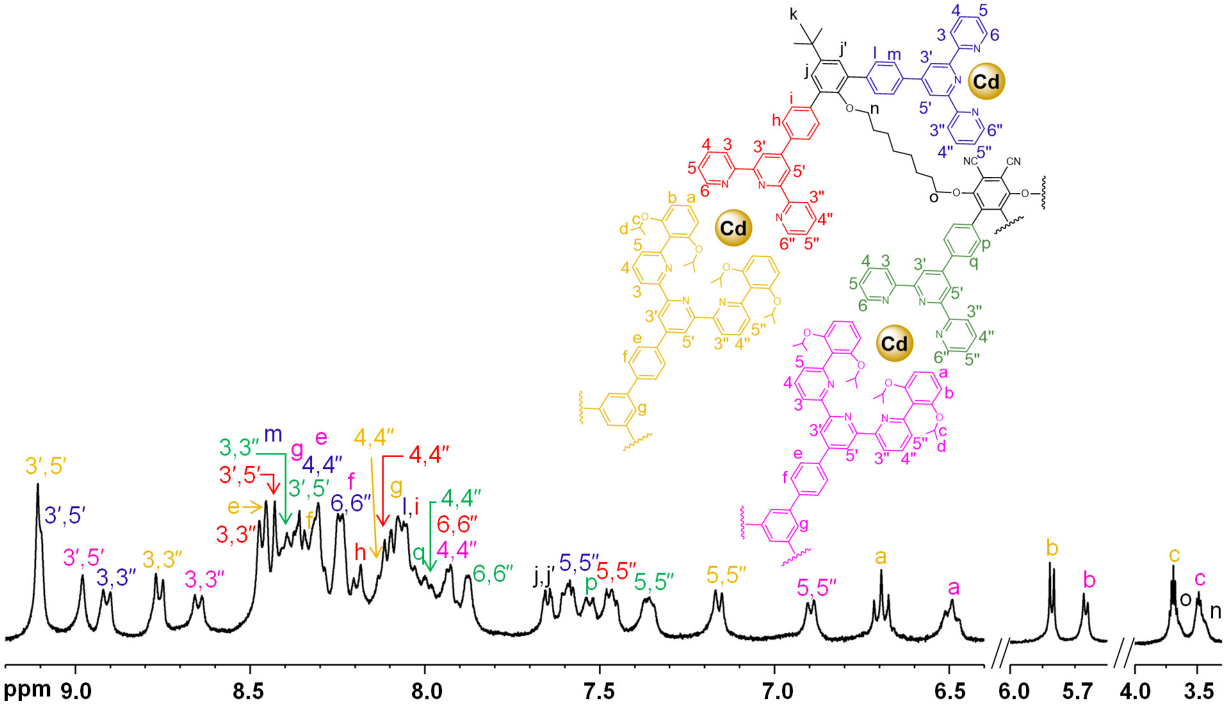

Figure S46. Partial ^{1}H NMR spectrum (400 MHz, CD_3CN) of [Cd_{30}**F**$^{1}{}_{8}$**V**$_{6}$].

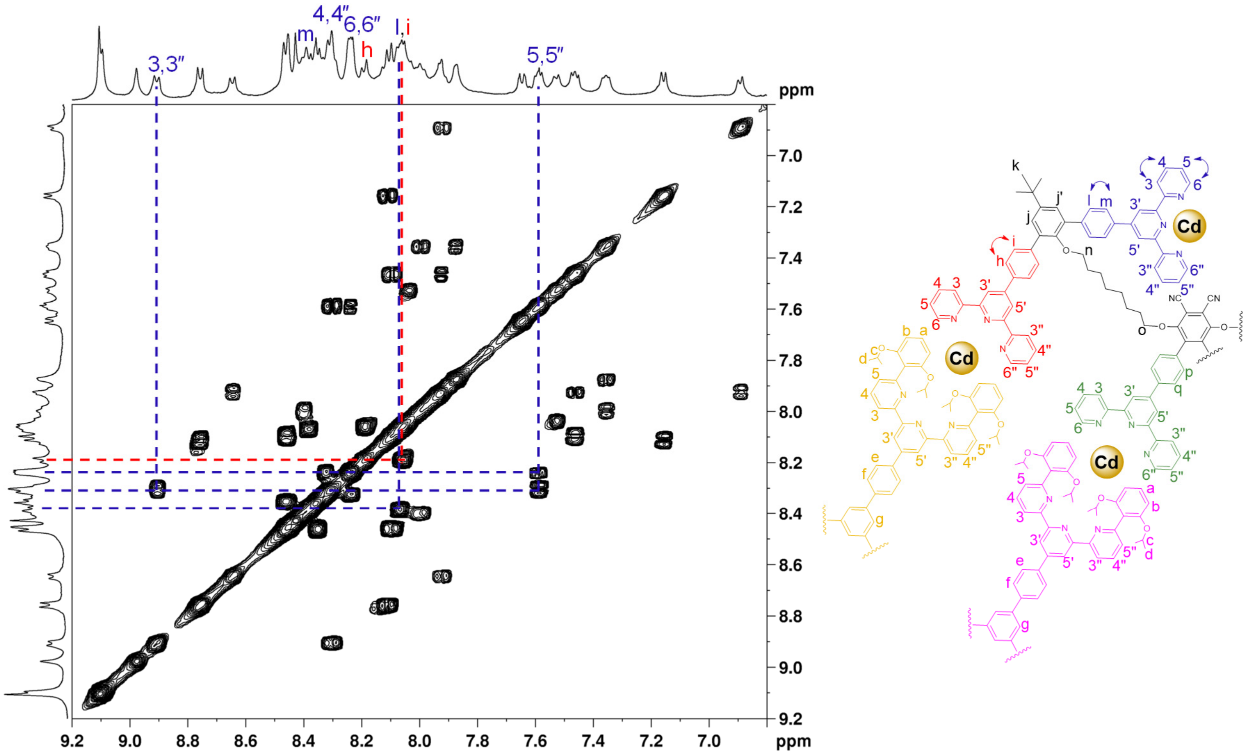

Figure S47. Partial COSY spectrum (500 MHz, CD_3CN)of [Cd_{30}**F**[1]$_8$**V**$_6$].

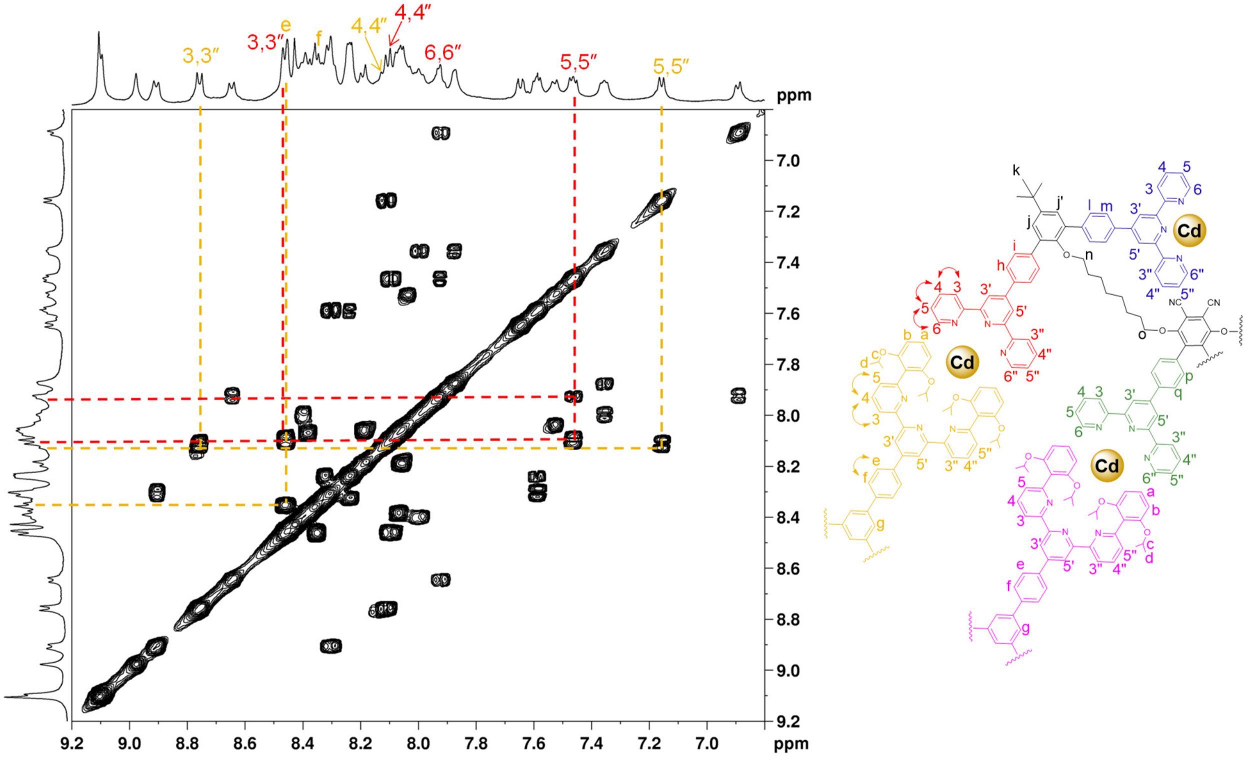

Figure S48. Partial COSY spectrum (500 MHz, CD_3CN) of [Cd_{30}**F**[1]$_8$**V**$_6$].

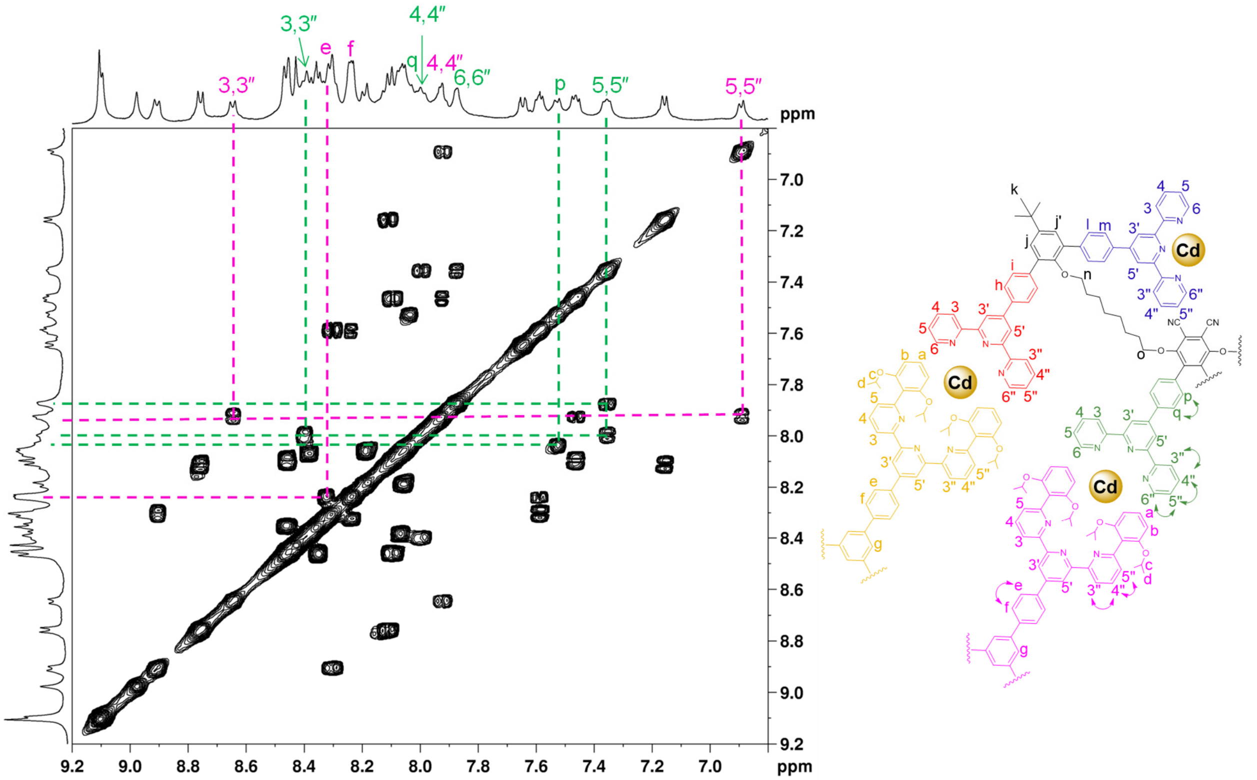

Figure S49. Partial COSY spectrum (500 MHz, CD_3CN) of [$Cd_{30}\mathbf{F^1}_8\mathbf{V}_6$].

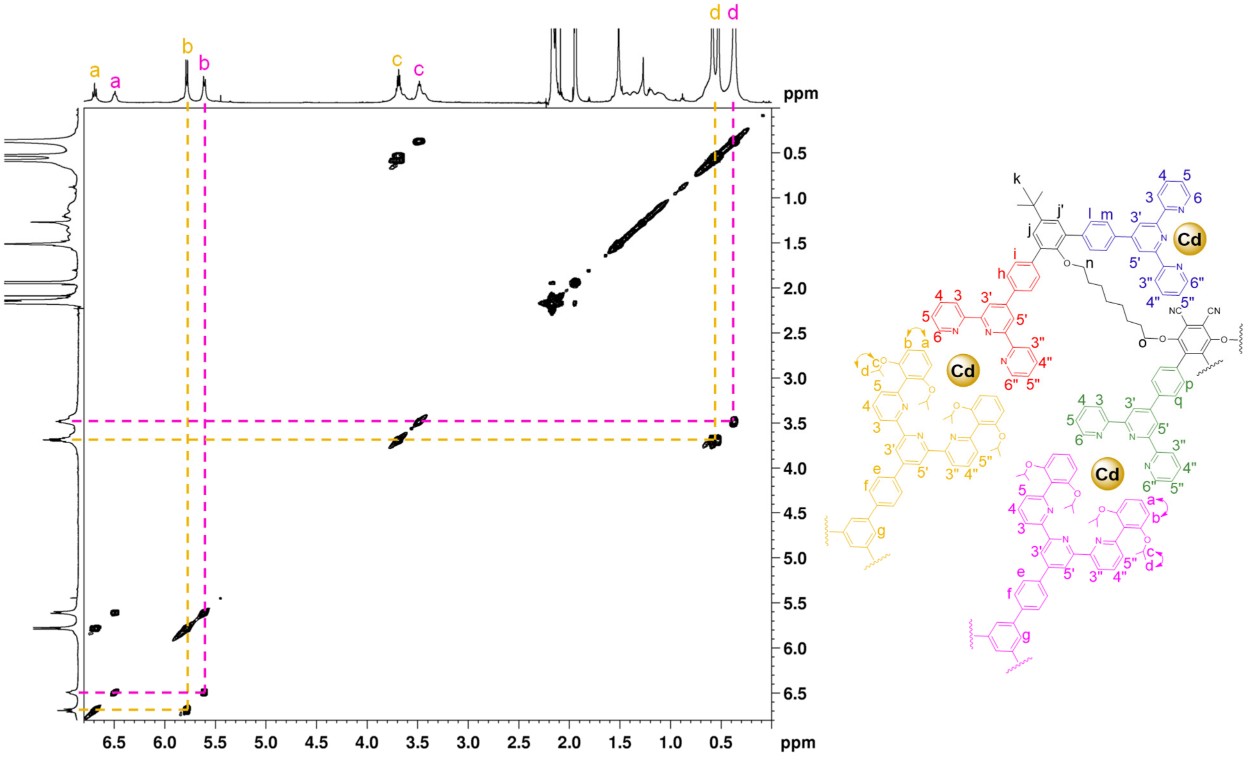

Figure S50. Partial COSY spectrum (500 MHz, CD_3CN) of [$Cd_{30}\mathbf{F^1}_8\mathbf{V}_6$].

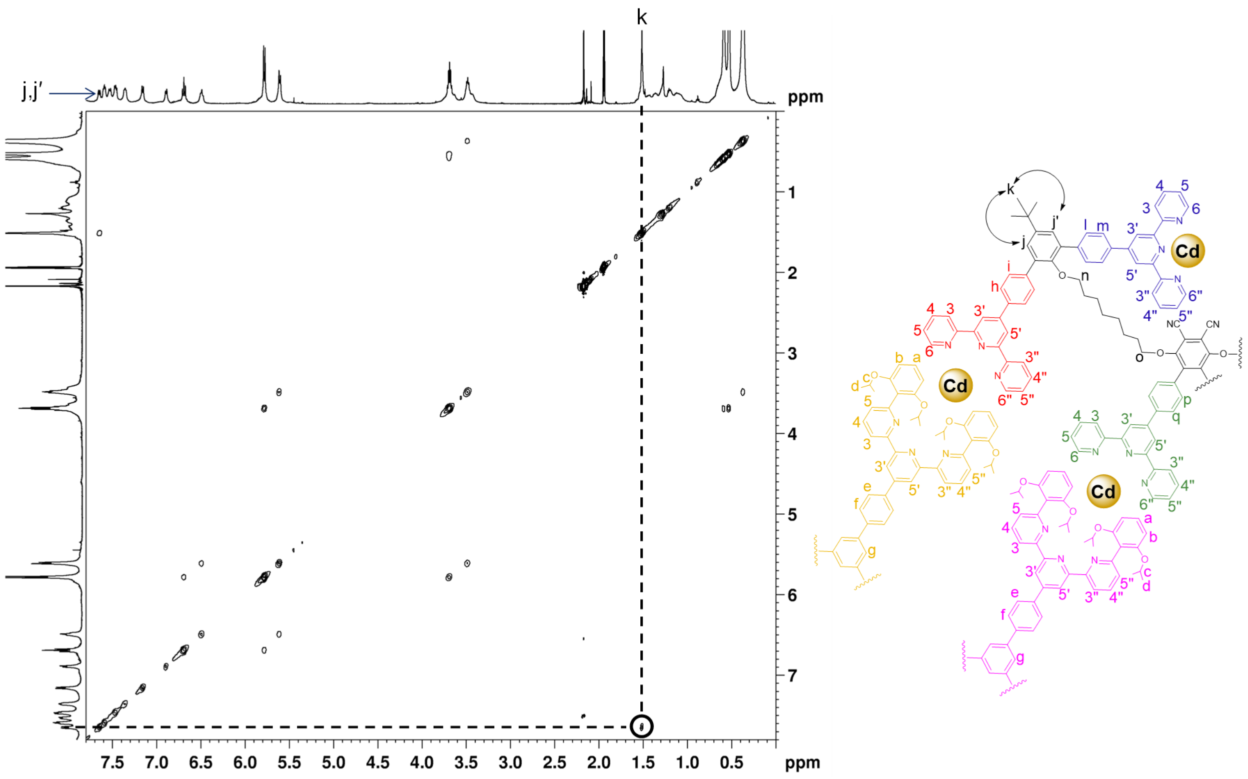

Figure S51. Partial ROESY spectrum (500 MHz, CD_3CN) of [Cd_{30}**F^1**$_8$**V**$_6$].

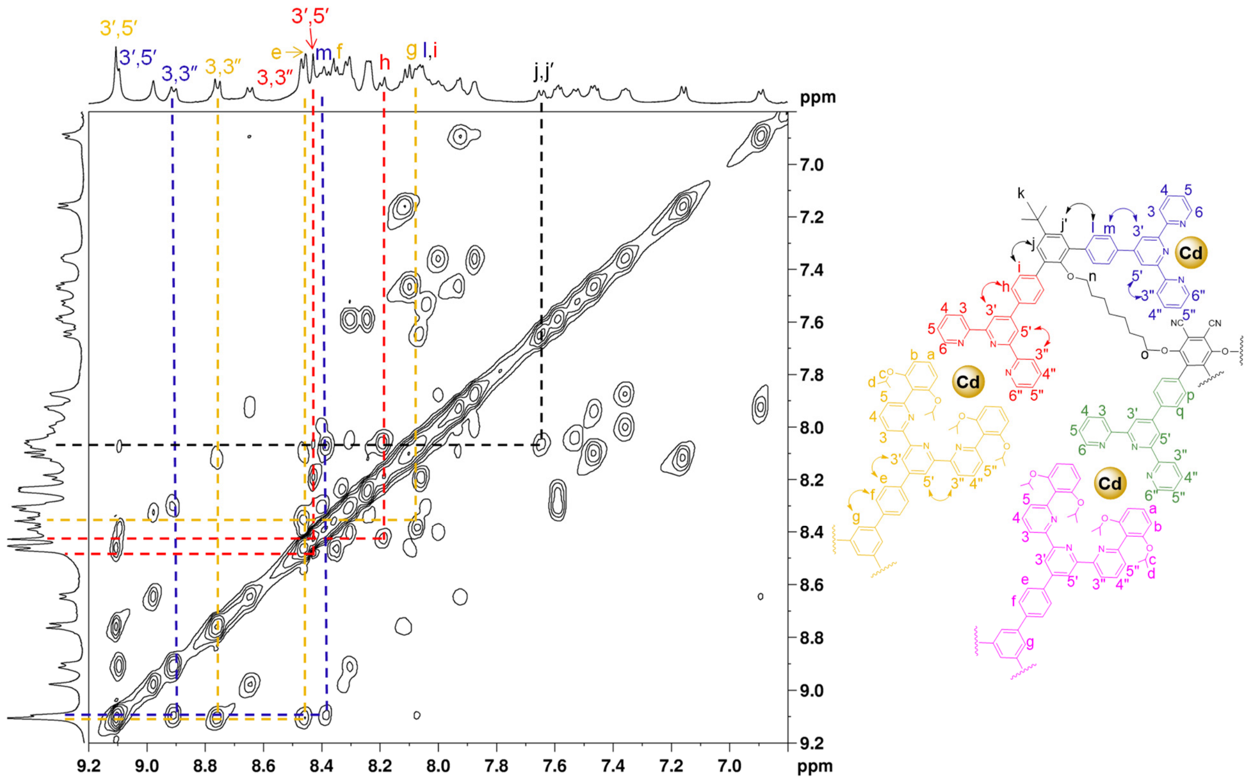

Figure S52. Partial ROESY spectrum (500 MHz, CD_3CN) of [Cd_{30}**F^1**$_8$**V**$_6$].

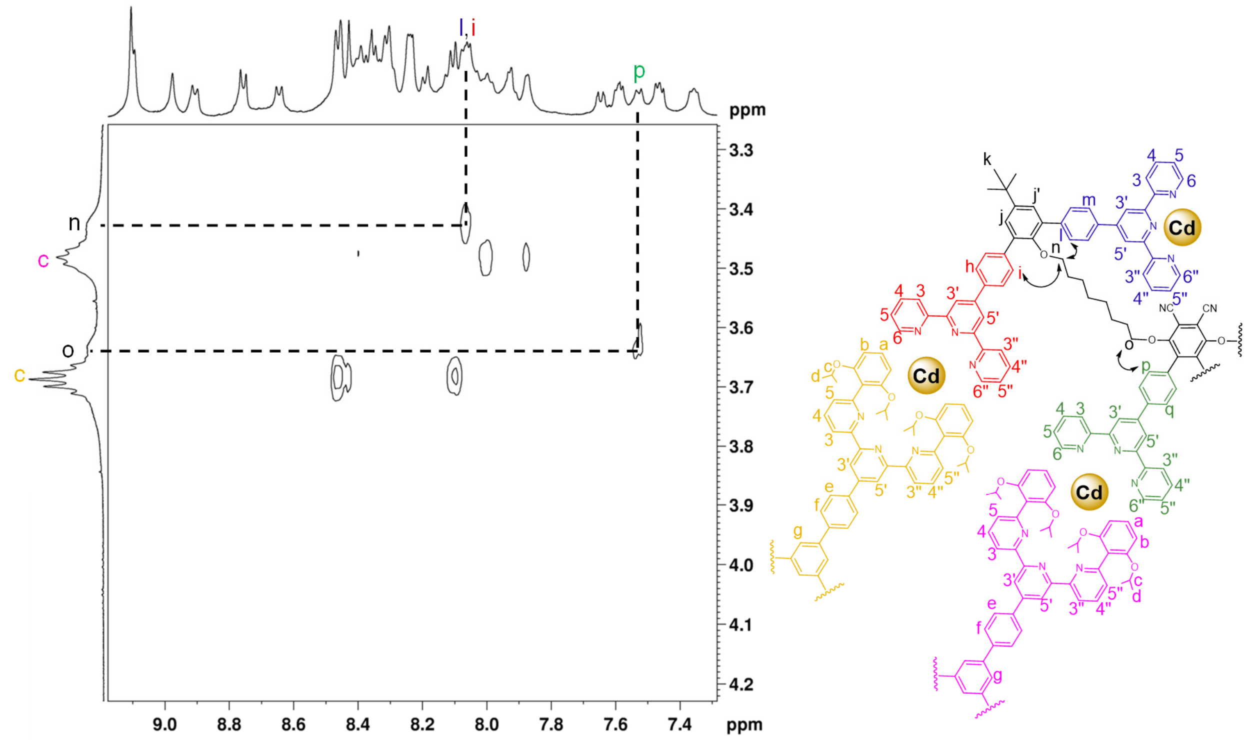

Figure S53. Partial ROESY spectrum (500 MHz, CD_3CN) of [Cd_{30}**F^1**$_8$**V**$_6$].

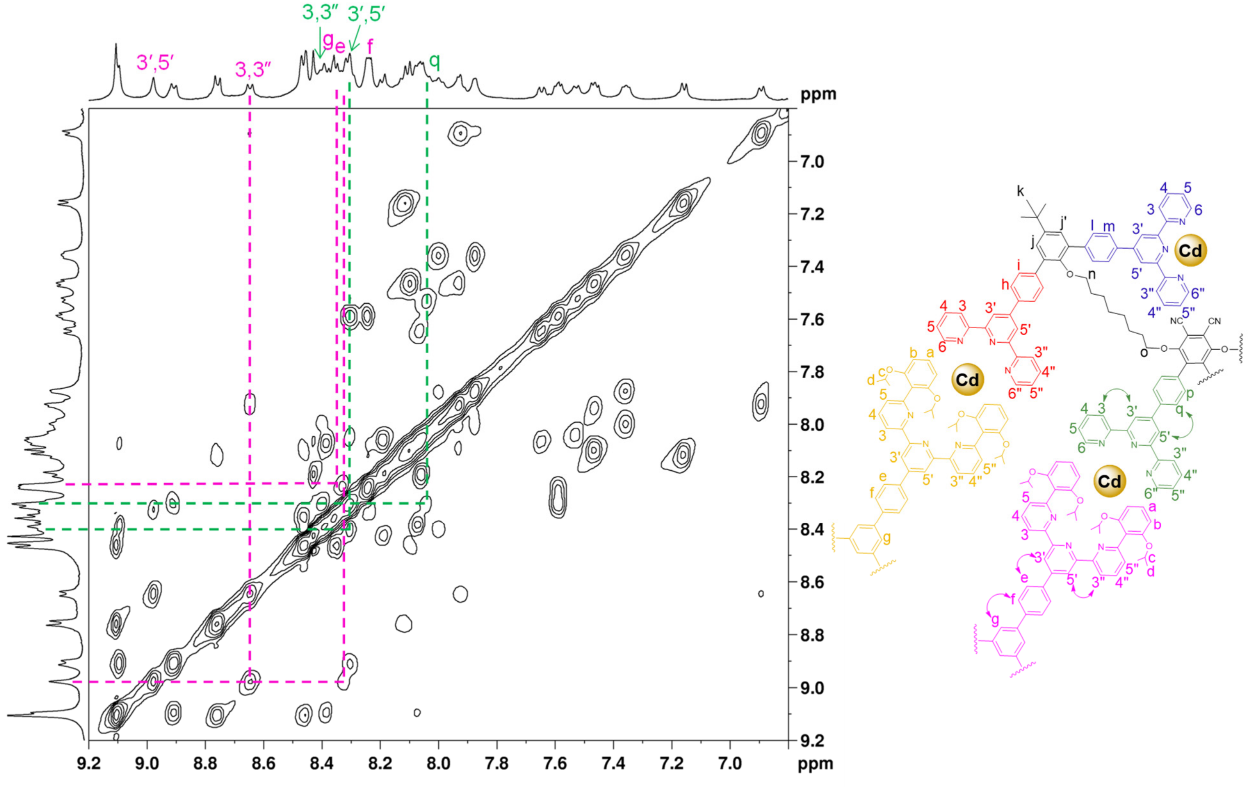

Figure S54. Partial ROESY spectrum (500 MHz, CD_3CN) of [Cd_{30}**F^1**$_8$**V**$_6$].

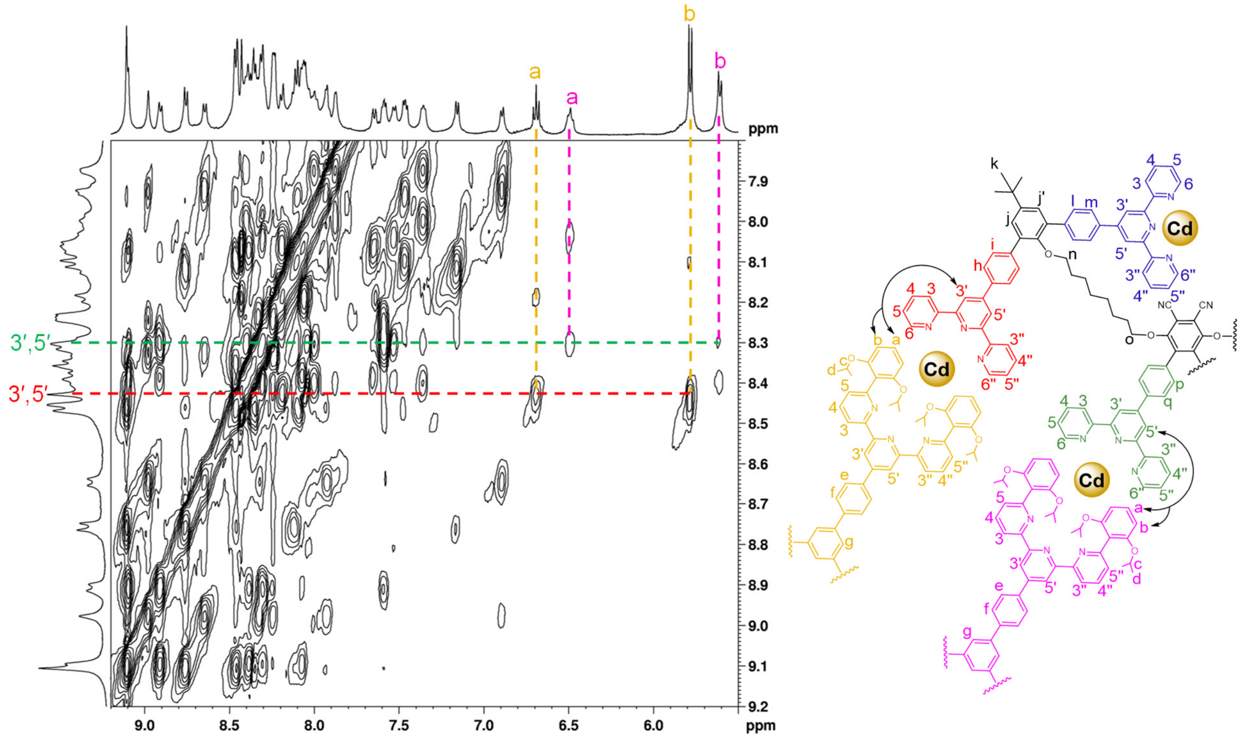

Figure S55. Partial ROESY spectrum (500 MHz, CD_3CN) of [Cd_{30}**F**^{1}$_8$**V**$_6$].

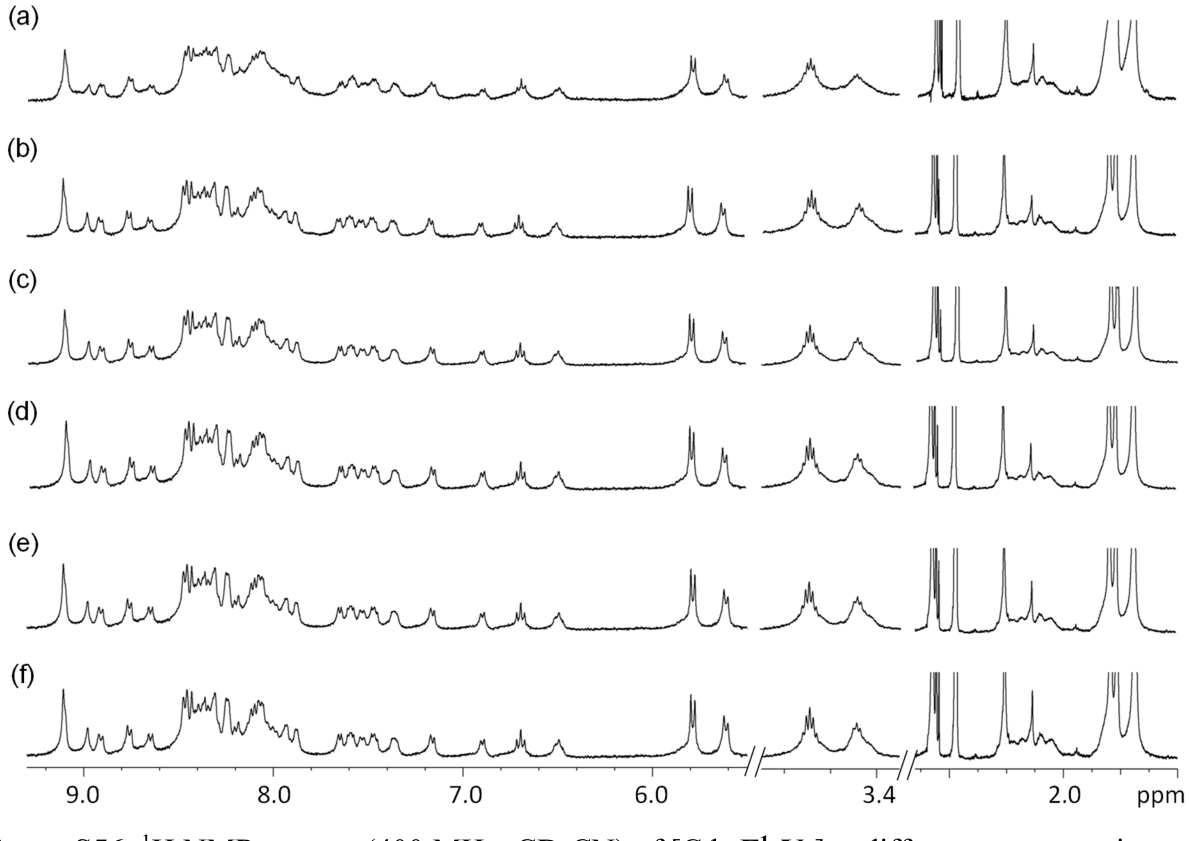

Figure S56. ^{1}H NMR spectra (400 MHz, CD_3CN) of [Cd_{30}**F**^{1}$_8$**V**$_6$] at different concentrations: (a) 1, (b) 2, (c) 4, (d) 8, (e) 16, and (f) 32 mg mL^{-1}.

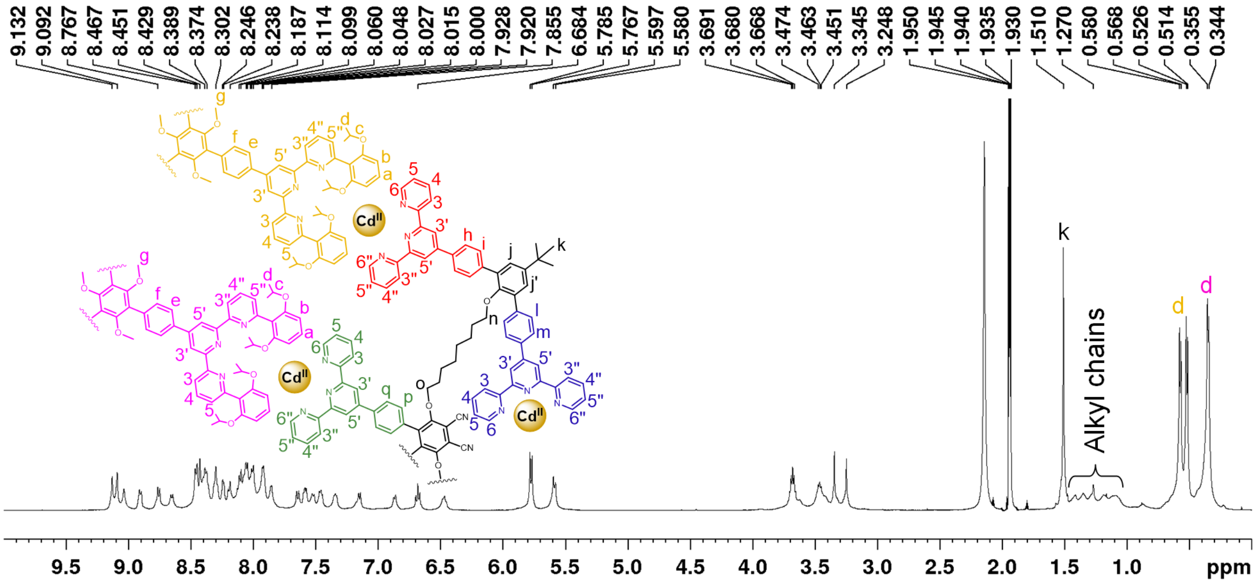

Figure S57. ^{1}H NMR spectrum (500 MHz, CD_3CN) of [Cd_{30}**F**$^2{}_8$**V**$_6$].

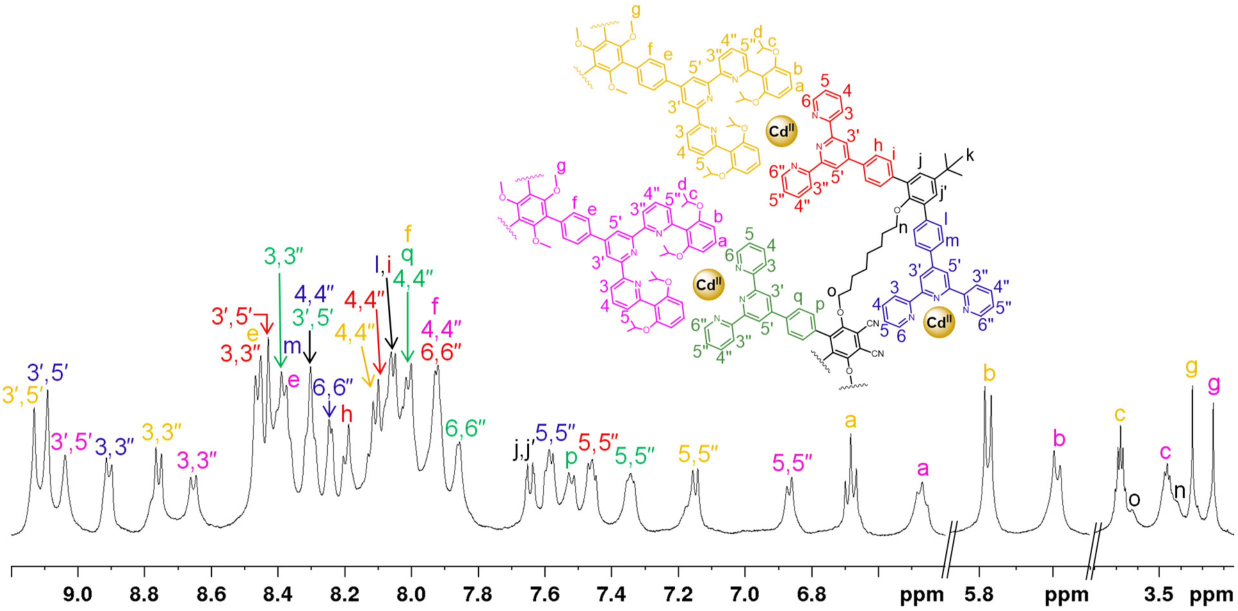

Figure S58. Partial ^{1}H NMR spectrum (500 MHz, CD_3CN) of [Cd_{30}**F**$^2{}_8$**V**$_6$].

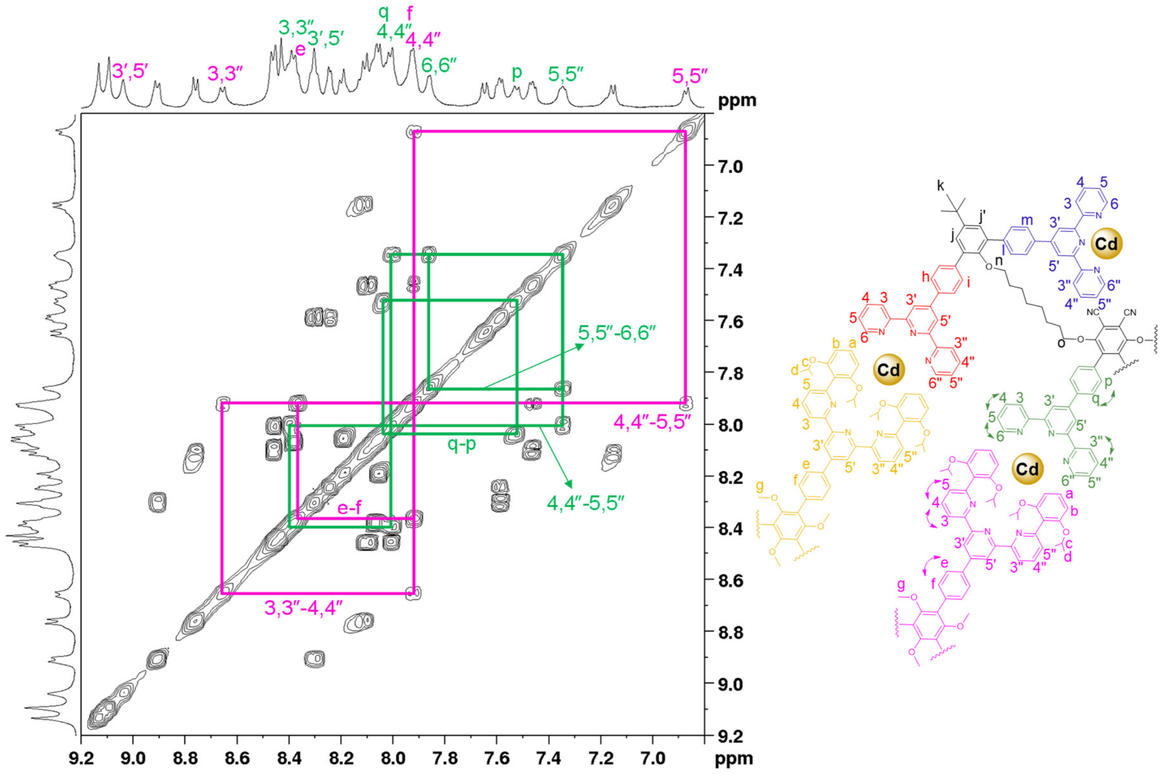

Figure S59. Partial COSY spectrum (500 MHz, CD_3CN) of [$Cd_{30}$$\mathbf{F^2}_8$$\mathbf{V}_6$].

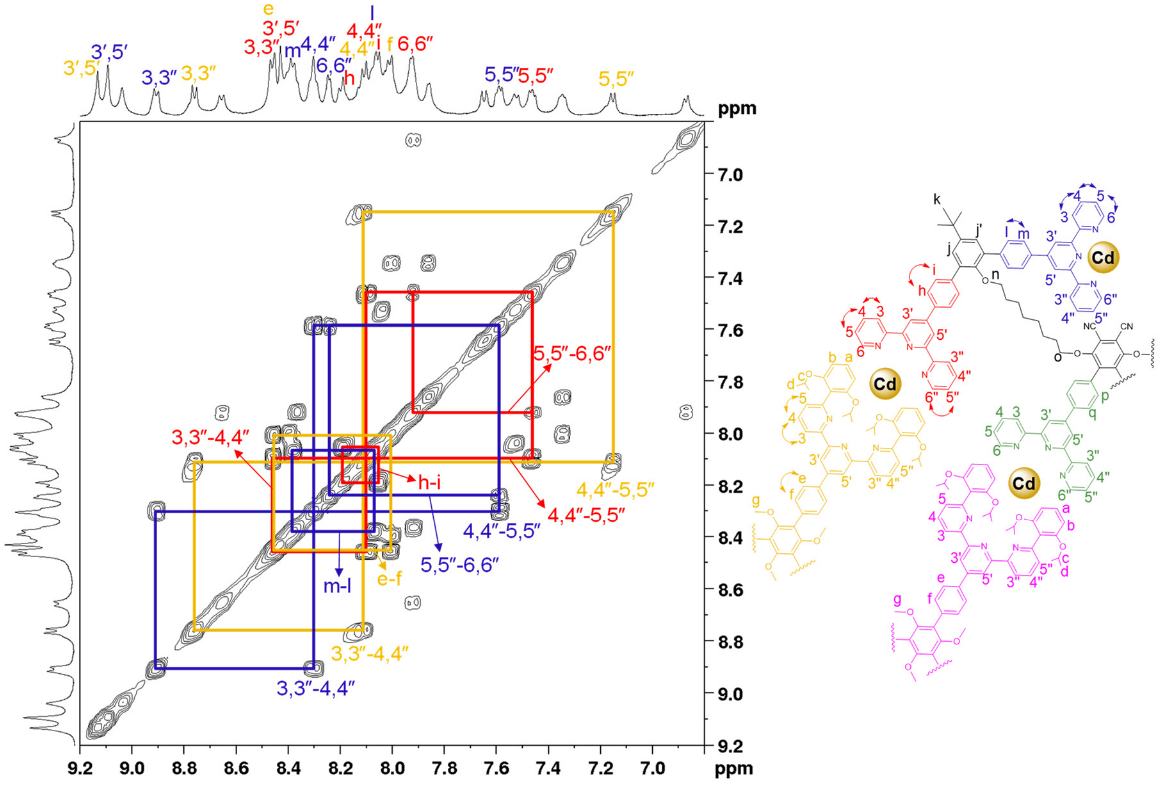

Figure S60. Partial COSY spectrum (500 MHz, CD_3CN) of [$Cd_{30}$$\mathbf{F^2}_8$$\mathbf{V}_6$].

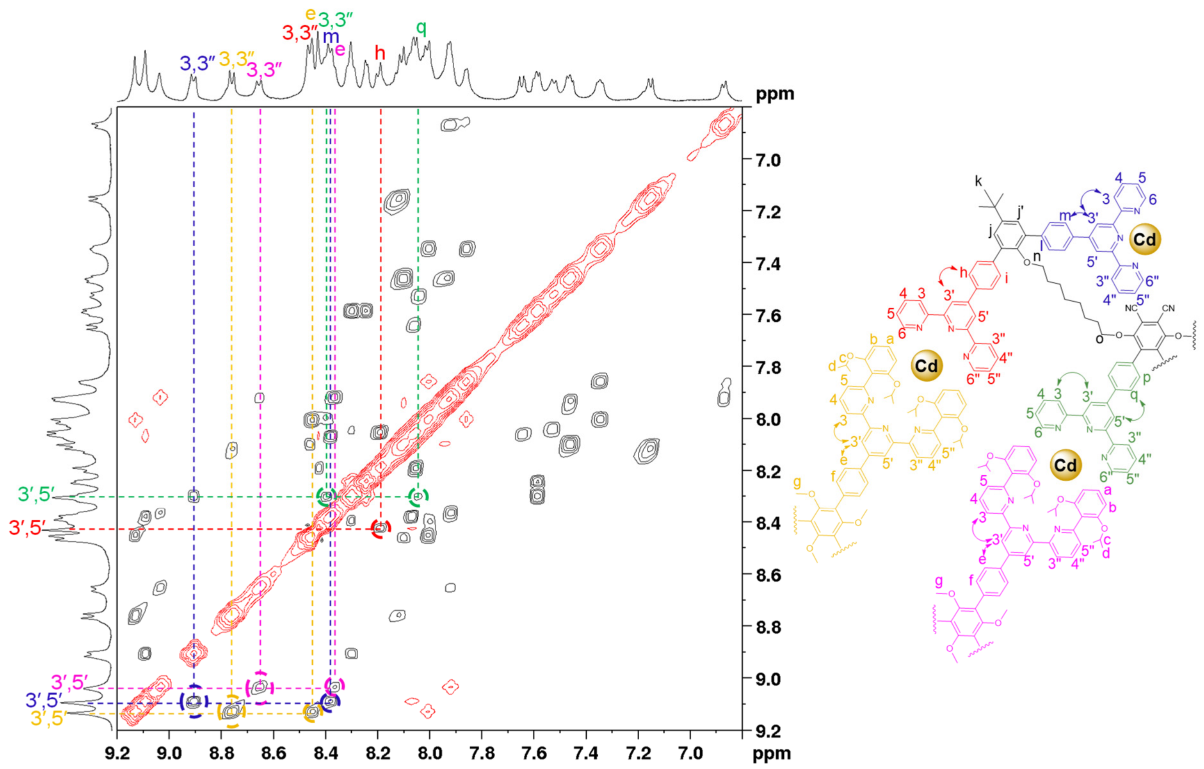

Figure S61. Partial ROESY spectrum (500 MHz, CD_3CN) of [$Cd_{30}$$\mathbf{F^2}_8$$\mathbf{V}_6$].

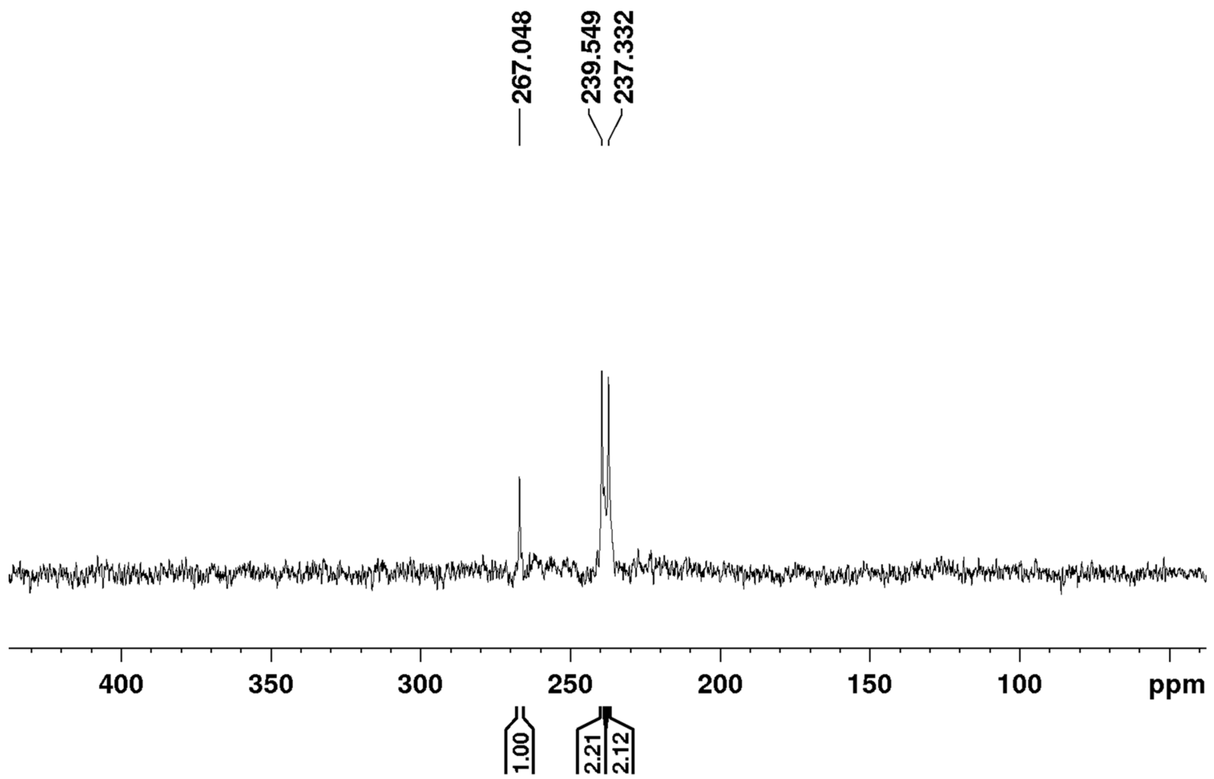

Figure S62. ^{113}Cd NMR spectrum (500 MHz, CD_3CN) of [$Cd_{30}$$\mathbf{F^2}_8$$\mathbf{V}_6$].

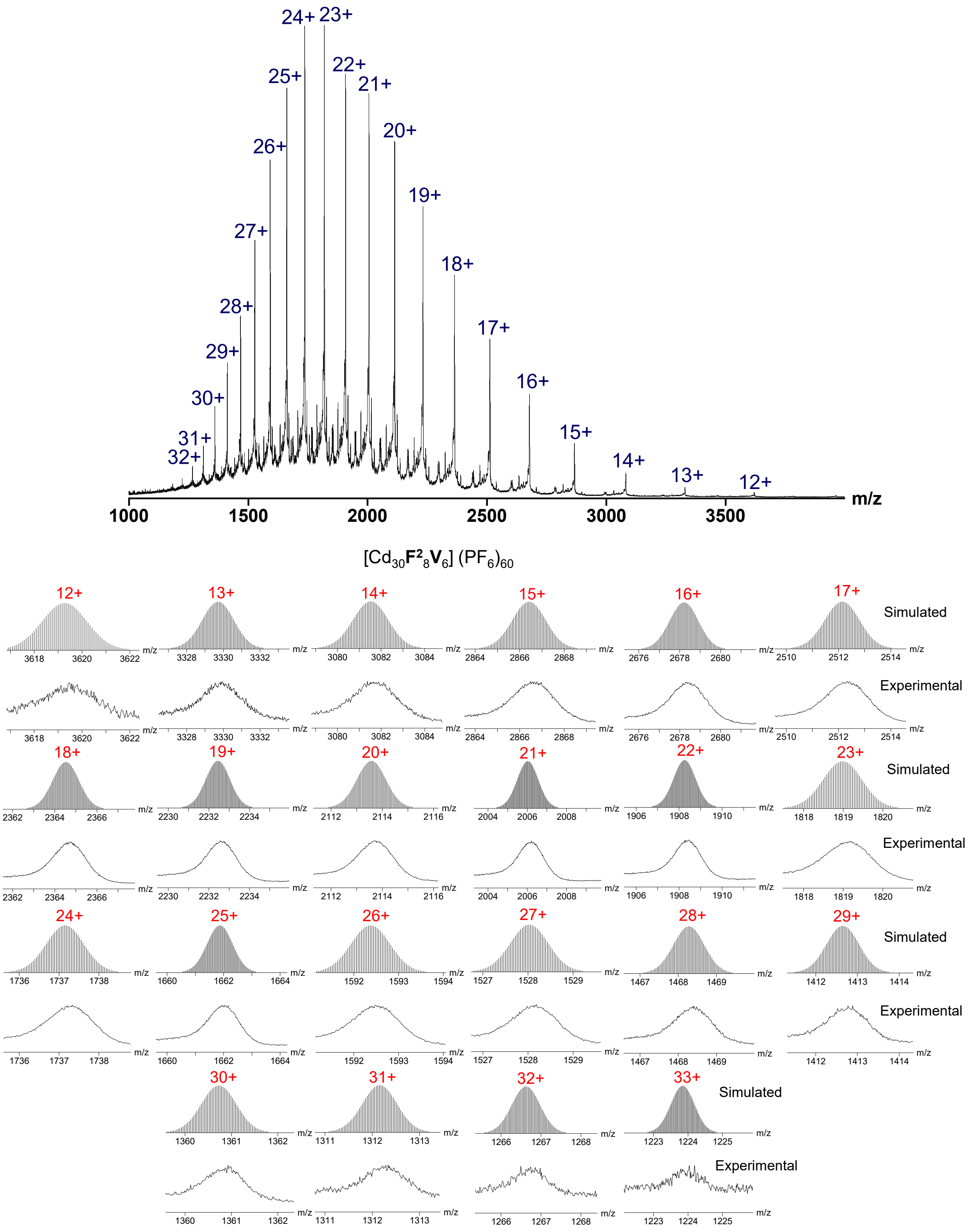

Figure S63. ESI-MS spectrum of $[Cd_{30}\mathbf{F^{2}}_{8}\mathbf{V}_{6}]$ with the experimental and simulated isotope patterns.

Ligand Exchange Experiments. The ligand exchange process between two cages, [$Cd_{30}\mathbf{F^1}_8\mathbf{V}_6$] and [$Cd_{30}\mathbf{F^2}_8\mathbf{V}_6$], was monitored using ESI-MS. To evaluate the ionization efficiency of [$Cd_{30}\mathbf{F^1}_8\mathbf{V}_6$] and [$Cd_{30}\mathbf{F^2}_8\mathbf{V}_6$], the ESI-MS experiments were performed on several mixtures of these complexes. MeCN solutions of [$Cd_{30}\mathbf{F^1}_8\mathbf{V}_6$] ($8.72 \times 10^{-5}$ M) and [$Cd_{30}\mathbf{F^2}_8\mathbf{V}_6$] ($8.72 \times 10^{-5}$ M) were prepared and mixed in various volumetric ratios (8:12, 9:11, 10:10, 11:9, and 12:8). The resulting mixtures were subjected to ESI-MS analysis (Figure S64). The peak integration ratio of [$Cd_{30}\mathbf{F^1}_8\mathbf{V}_6$] relative to the total concentration of [$Cd_{30}\mathbf{F^1}_8\mathbf{V}_6$] and [$Cd_{30}\mathbf{F^2}_8\mathbf{V}_6$] was plotted against the corresponding molar traction of [$Cd_{30}\mathbf{F^1}_8\mathbf{V}_6$] (Figure S66b). The observed linear relationship indicated that [$Cd_{30}\mathbf{F^1}_8\mathbf{V}_6$] and [$Cd_{30}\mathbf{F^2}_8\mathbf{V}_6$] exhibited equal ionization efficiency. Following the confirmation of ionization efficiency, a mixture containing [$Cd_{30}\mathbf{F^1}_8\mathbf{V}_6$] ($4.36 \times 10^{-5}$ M in MeCN, 2.0 mL) and [$Cd_{30}\mathbf{F^2}_8\mathbf{V}_6$] ($4.36 \times 10^{-5}$ M in MeCN, 2.0 mL) was analyzed by ESI-MS at various time intervals after mixing at 25 °C: 0.6 h, 73.5 h, 193.8 h, 266.4 h, and 291.7 h (Figure S65). Although ligand exchange could occur within both the inner and outer frameworks of the cages, the corresponding exchange rates were assumed to be comparable. A gradually emerging peak was assigned to the ligand-exchanged [$Cd_{30}\mathbf{F^1}_7\mathbf{F^2}_1\mathbf{V}_6$]. From the peak integration ratios of [$Cd_{30}\mathbf{F^1}_8\mathbf{V}_6$] and [$Cd_{30}\mathbf{F^1}_7\mathbf{F^2}_1\mathbf{V}_6$], the concentrations of [$Cd_{30}\mathbf{F^1}_8\mathbf{V}_6$] at various mixing times were determined. The corresponding kinetic plot (Figure S66c) revealed an apparent rate constant (k_{obs}) of 4.20 M^{-1} h^{-1} and the calculated half-life of [$Cd_{30}\mathbf{F^1}_8\mathbf{V}_6$] was estimated to be 227 days.

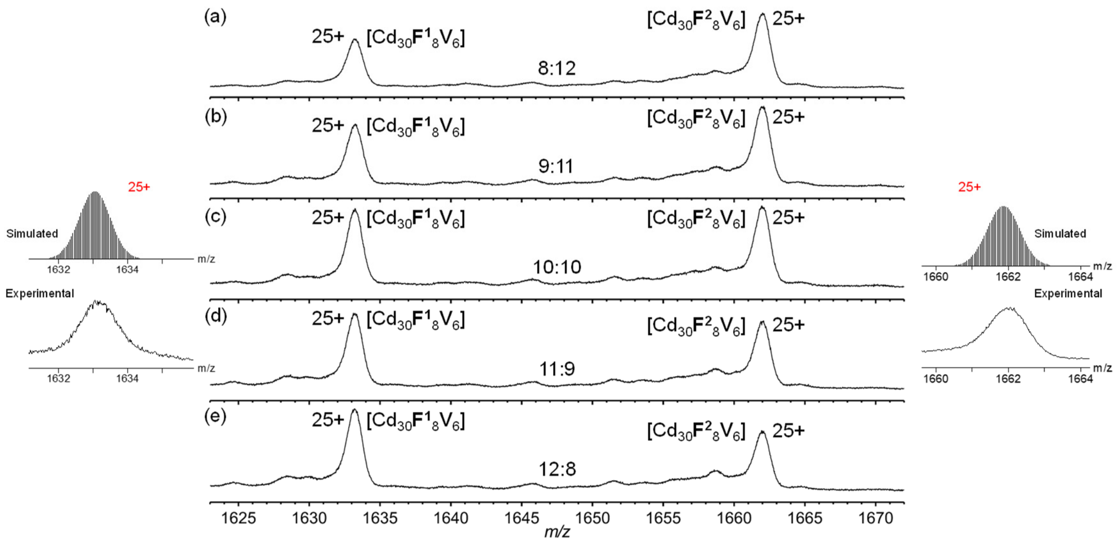

Figure S64. Partial ESI-MS spectra of a mixture of [$Cd_{30}\mathbf{F^1}_8\mathbf{V}_6$] and [$Cd_{30}\mathbf{F^2}_8\mathbf{V}_6$] for 25+ ions at different volumetric ratios: (a) 8:12, (b) 9:11, (c) 10:10, (d) 11:9, and (e) 12:8. The total concentration was fixed to 8.72×10^{-5} M for each measurement.

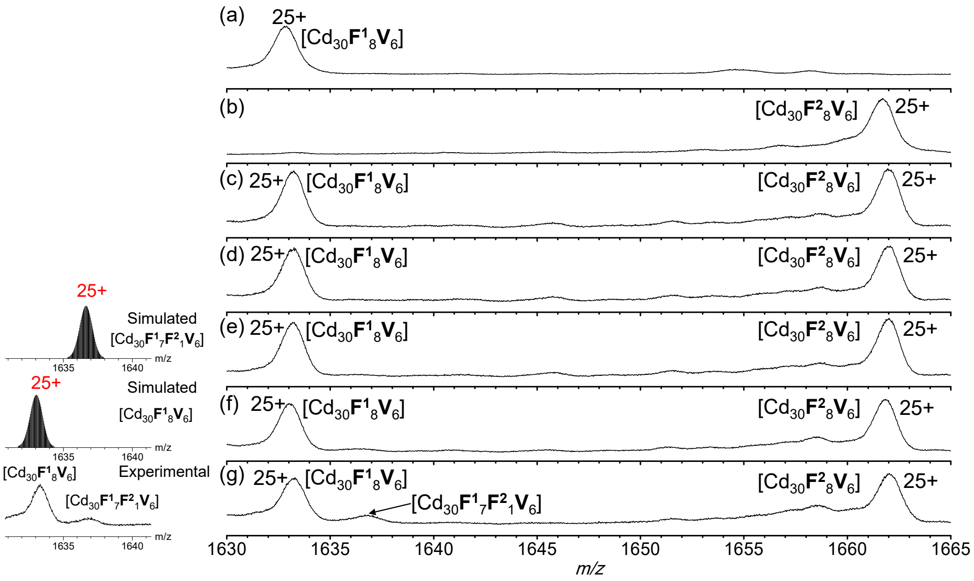

Figure S65. Partial ESI-MS spectra (MeCN, PF_6^- salt) of (a) [Cd_{30}**F**[1]$_8$**V**$_6$], (b) [Cd_{30}**F**[2]$_8$**V**$_6$], an equimolar mixture of [Cd_{30}**F**[1]$_8$**V**$_6$] and [Cd_{30}**F**[2]$_8$**V**$_6$] for 25+ ions mixed after (c) 0.6 h, (d) 23.0 h, (e) 73.5 h, (f) 291.7 h, and (g) 1248.9 h.

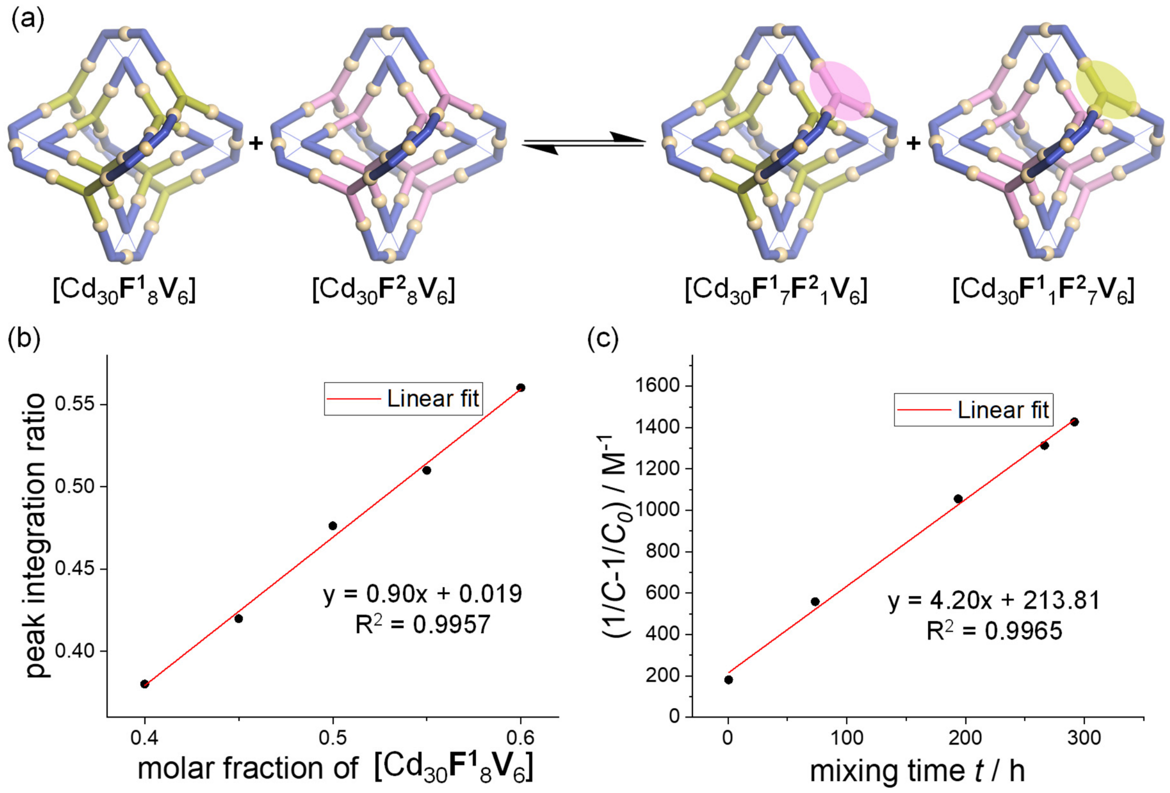

Figure S66. Ligand exchange experiments. (a) Cartoon representation of the tritopic ligand exchange between [Cd_{30}**F**[1]$_8$**V**$_6$] and [Cd_{30}**F**[2]$_8$**V**$_6$]. (b) Plot of peak integration ratio against the molar fraction ratio of [Cd_{30}**F**[1]$_8$**V**$_6$]. (c) Plot of ($1/C$–$1/C_0$) against the mixing time. C_0 and C are the initial concentration and the concentration after a certain mixing time for [Cd_{30}**F**[1]$_8$**V**$_6$], respectively.

To compare the k_{obs} with the ligand exchange rate of [Cd**L**^{c}**L**^{d}], the experiment illustrated in Figure S67a was conducted. An equimolar amount of free ligand **L**^{c} and [Cd**L**^{c}**L**^{d}] (1.0 mM each in CD_3CN) was prepared, and the ligand exchange rate was determined using ^1H exchange spectroscopy (EXSY) NMR experiments.[16] The integration of diagonal (I_{AA} and I_{BB}) and cross (I_{AB} and I_{BA}) peaks was acquired under various mixing time (100–350 ms). To avoid peak overlap, the signals corresponding to the H^c protons of both coordinated and uncoordinated **L**^{c} were recorded. A plot of $\ln[(r+1)/(r–1)]$ as a function of mixing time (T_m) was generated, where $r = 4X_AX_B(I_{AA}+I_{BB})/(I_{AB}+I_{BA}) – (X_A–X_B)^2$. Here, X_A and X_B represent the mole fractions of **L**^{c} and [Cd**L**^{c}**L**^{d}], respectively.[17] The exchange rate constant ($k_{ex} = k_1 + k_{-1}$) was calculated from the slope of linear regression of $\ln[(r+1)/(r–1)]$ against T_m (Figure S67b). The exchange rate constant at 25 °C (k_{ex}) was estimated to be 2.05×10^{-2} s^{-1}, corresponding to a half-life of 34 s. The significantly shorter half-life observed for [Cd**L**^{c}**L**^{d}] indicated that the nested cage structure exhibited enhanced stability compared to the mononuclear heteroleptic complex.

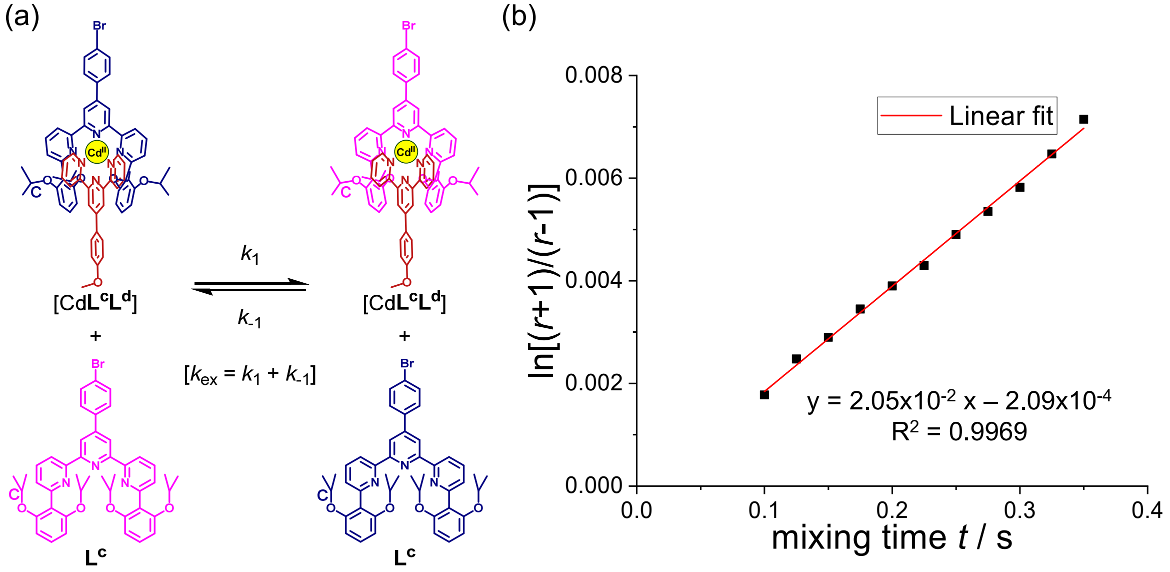

Figure S67. (a) Schematic illustration of the ligand exchange reaction between [Cd**L**^{c}**L**^{d}]$(PF_6)_2$ and **L**^{c}. (b) Plot of $\ln[(r+1)/(r–1)]$ against T_m generated from the EXSY experiments in CD_3CN at 298 K.

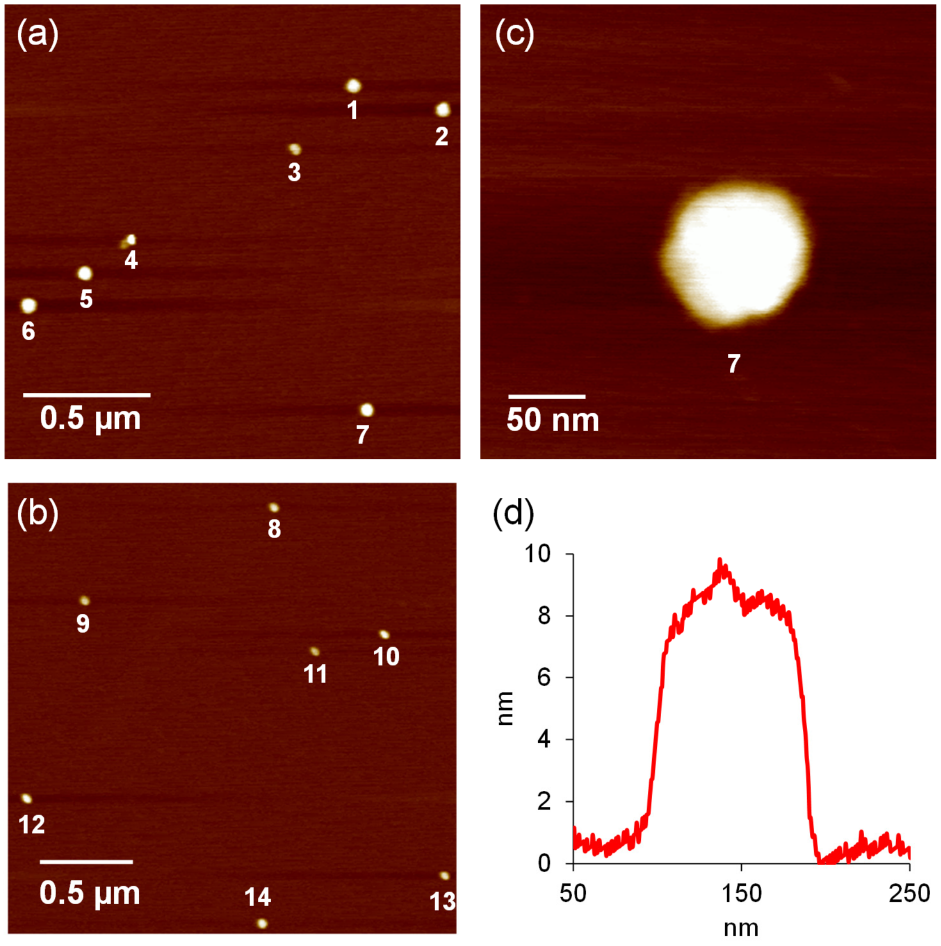

Spot	Height (nm)
1	7.6
2	8.6
3	5.8
4	8.7
5	8.7
6	8.6
7	8.6
8	6.0
9	5.3
10	6.1
11	5.3
12	6.3
13	6.1
14	6.1

Figure S68. (a-c) AFM images of [$Cd_{30}\mathbf{F}^{1}{}_{8}\mathbf{V}_{6}$] and (d) the height profile of spot 7.

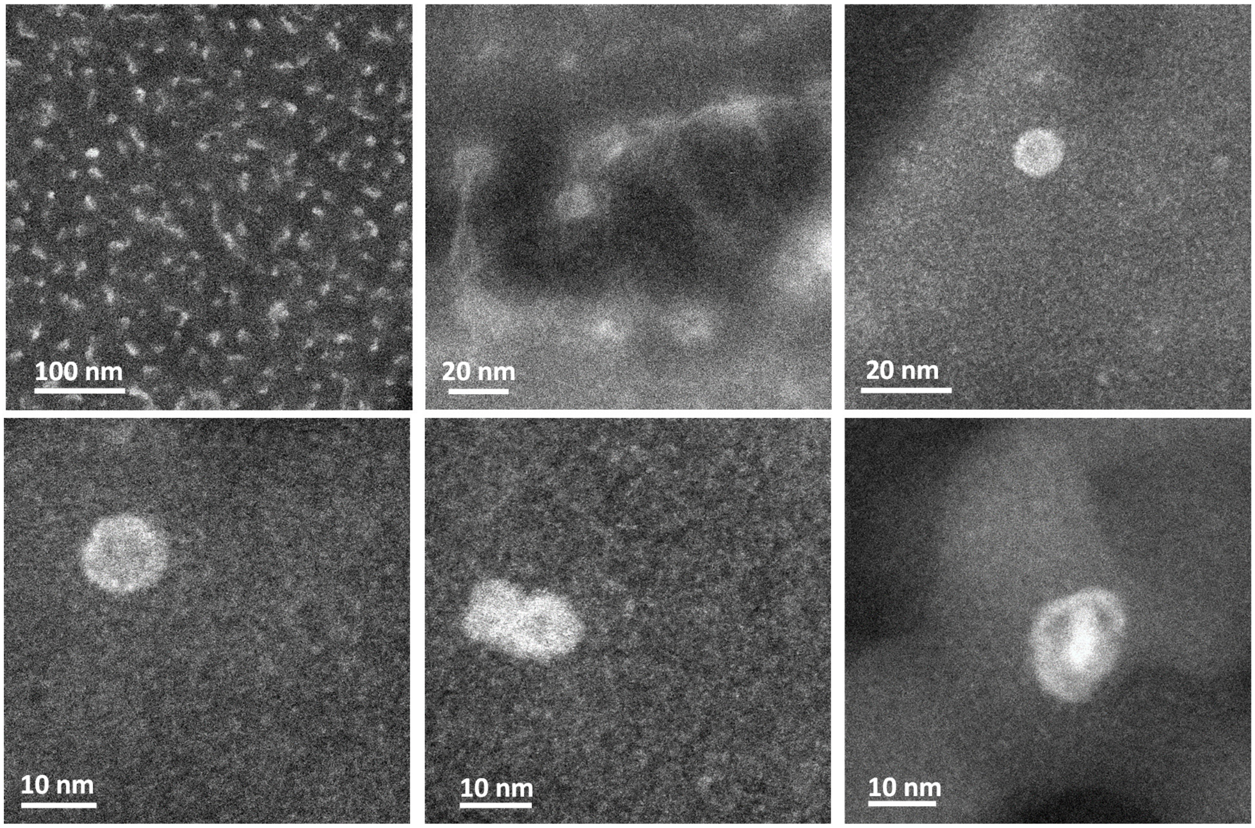

Figure S69. HAADF-STEM images of [$Cd_{30}$$\mathbf{F^1}_8$$\mathbf{V}_6$].

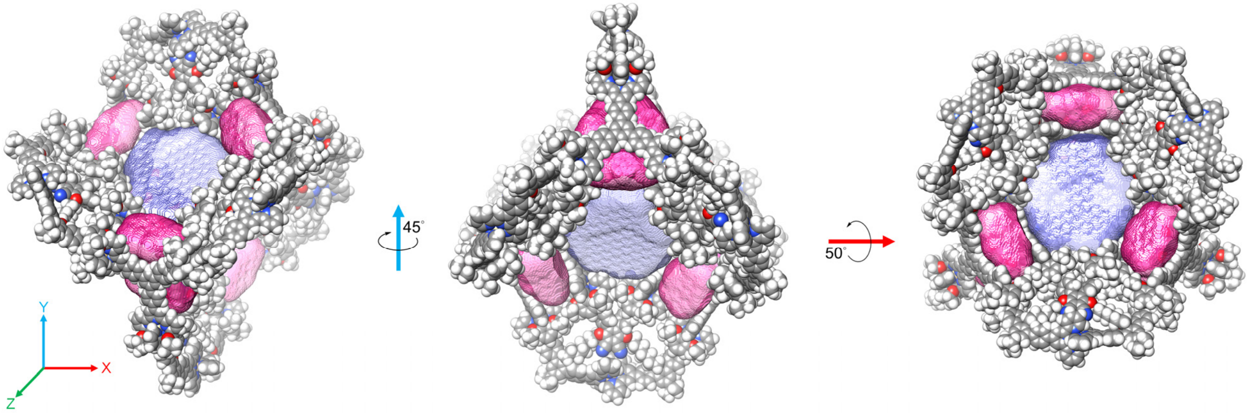

Figure S70. Computed cavities of [$Cd_{30}$$\mathbf{F^1}_8$$\mathbf{V}_6$] generated using MoloVol in a single-probe mode with a probe radius of 3.7 Å and a grid resolution of 0.2 Å. The central cavity (14,847 Å^3), visualized in purple, is surrounded by four smaller cavities (3,280 ± 94 Å^3), shown in pink.

Synthesis of Au@Cage. To a vial containing MeCN (400 μL) and [Cd_{30}**F**$^1{}_8$**V**$_6$] (50 μL, 10^{-4} M in MeCN), $HAuCl_4 \cdot 3H_2O$ (50 μL, 1 mg mL^{-1} in MeCN) was added. The mixture was stirred at room temperature for 1 h. Subsequently, $NaBH_4$ (20 μL, 1 mg mL^{-1} in MeOH) was added to the solution, which was stirred for an additional 1 h. After the solvent was removed under reduced pressure, the residue was washed twice with MeOH (0.5 mL) and the solvent was carefully removed by a glass pipette. The resultant solid was dried *in vacuo* to afford Au@Cage, which was re-dispersed in MeCN (3 mL).

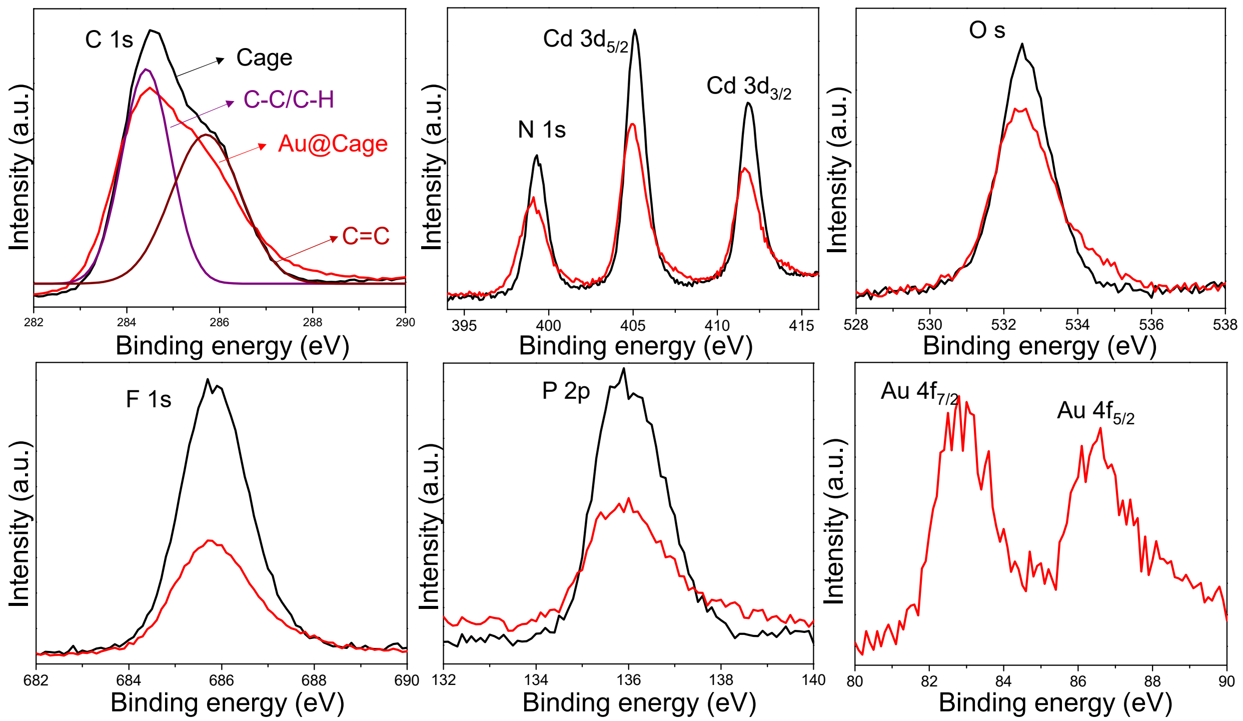

Figure S71. XPS analysis for [Cd_{30}**F**$^1{}_8$**V**$_6$] (black line) and Au@Cage (red line).

Control Experiments. The control experiments were conducted following the same protocol as that of Au@Cage, using the flat metallomacrocycle (FM), [Cd_9**V**$_3$], (50 μL, 8.8×10^{-5} M) and, separately in the absence of any metal complexes.

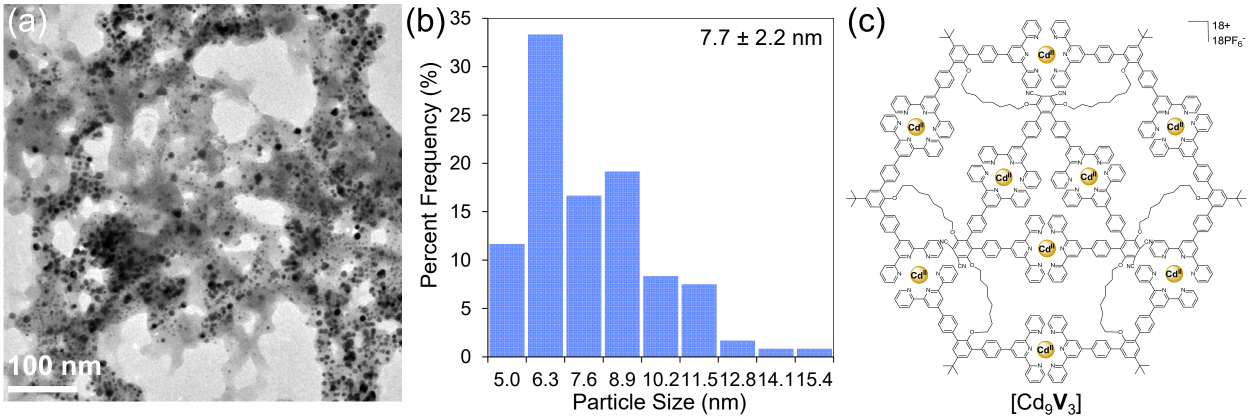

Figure S72. (a) TEM image and (b) particle size distribution histogram of Au@FM. (c) Chemical structure of FM.

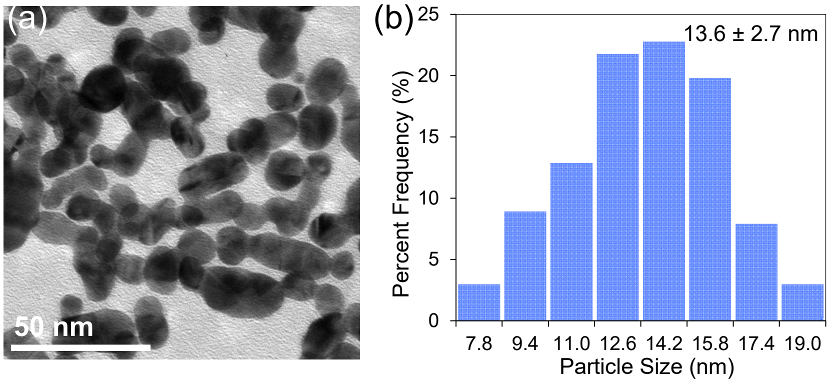

Figure S73. (a) TEM image and (b) particle size distribution histogram of pure Au nanoparticles.

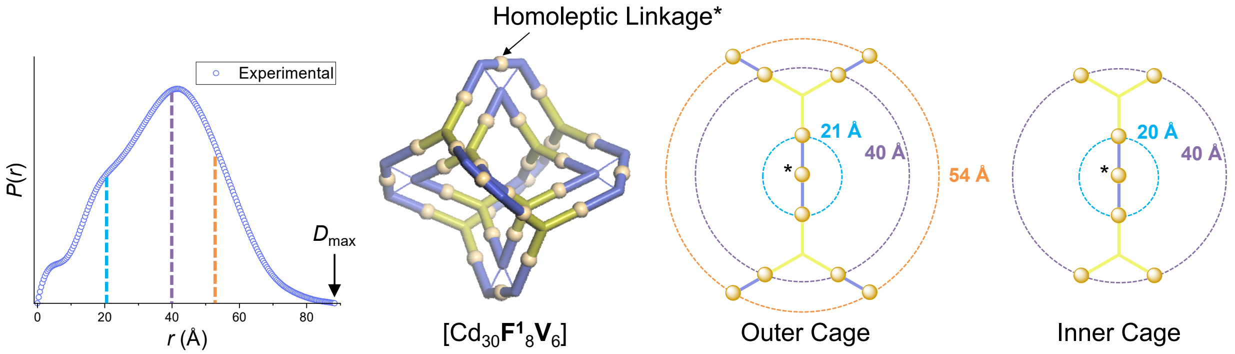

Figure S74. Pair distance distribution function derived from the experimental SAXS profile shown alongside the corresponding distances between the homoleptic metal center and the metal centers in the outer and inner cages, illustrated through a 2D unfolding of the nested cage structure.

Table S1. Summary of X-ray crystallographic data collection and refinement parameters for [Cd**L**[c]**L**[d]].

Parameters	[Cd**L**[c]**L**[d]]$(PF_6)_2$
Chemical formula	$C_{67}H_{63}BrCdF_{12}N_6O_5P_2$
M_r	1514.48
Temperature (K)	150 K
Morphology	cube
Crystal size	0.25 × 0.25 × 0.20
Crystal system	monoclinic
Space group	*P2(1)/c*
a (Å)	24.0525(5)
b (Å)	12.8501(3)
c (Å)	22.3831(6)
α (°)	90
β (°)	103.456(2)
γ (°)	90
V (Å^3)	6728.2(3)
Z	4
D_{calc} (g mm^{-3})	1.495
μ (mm^{-1})	1.05
F(000)	3072
T_{min}	0.973
T_{max}	1.0
h, k, l (min, max)	(-31, 31)
	(-16, 12)
	(-29, 28)
Reflns collected	47753
Unique reflns	15139
Observed reflns	10047
R_{int}	0.0428
No. of parameters	847
GoF	1.021
$R_1[I > 2\sigma(I)]$	0.0704
$wR_2[I > 2\sigma(I)]$	0.1942
R_1_all data	0.1019
wR_2_all data	0.2274
$\Delta\rho_{max}, \Delta\rho_{min}$(e Å^{-3})	1.8, -0.92
CCDC No.	2456141

Table S2. Experimental and theoretical collision cross-sections (CCSs) of [Cd_{30}**F^1**$_8$**V**$_6$](PF_6)$_{60}$.

Drift time (ms)	Exp. CCS (Å^2)	Average CCS (Å^2)	Calcd Avg. CCS (Å^2)
9.48 (24+)	3980.6		
9.15 (25+)	4046.9		
8.82 (26+)	4103.7		
8.38 (27+)	4112.7		
8.05 (28+)	4147.0		
7.83 (29+)	4212.5		
7.61 (30+)	4217.1		
7.17 (31+)	4230.1		3398.5±98.8[a]
6.95 (32+)	4270.2	4214.3±110.6	
6.62 (33+)	4251.4		4192.2±144.9[b]
6.39 (34+)	4268.8		
6.17 (35+)	4282.8		
5.95 (36+)	4288.4		
5.84 (37+)	4346.9		
5.73 (38+)	4401.6		

The calculated values were obtained by [a]projection approximation (PA) and [b]trajectory method (TM) from 170 annealed structures using MOBCAL.

References

(1) He, L.; Hsu, H.-K.; Li, L.; Lin, L.-T.; Tu, T.-H.; Ong, T.-G.; Liou, G.-G.; Chan, Y.-T., A 10-nm-sized multicompartment cuboctahedron and its 2D hierarchical arrays observed by cryo-EM. *Chem* **2022**, *8*, 494-507.
(2) Liang, Y.-P.; He, Y.-J.; Lee, Y.-H.; Chan, Y.-T., Self-assembly of triangular metallomacrocycles using unsymmetrical bisterpyridine ligands: isomer differentiation via TWIM mass spectrometry. *Dalton Trans.* **2015**, *44*, 5139-5145.
(3) Covey, T. R.; Bonner, R. F.; Shushan, B. I.; Henion, J.; Boyd, R. K., The determination of protein, oligonucleotide and peptide molecular weights by ion-spray mass spectrometry. *Rapid Comm. Mass Spectrom.* **1988**, *2*, 249-256.
(4) Guinier, A.; Fournet, G., *Small-angle Scattering of X-rays*. Wiley: 1955.
(5) Chan, Y.-T.; Li, X.; Yu, J.; Carri, G. A.; Moorefield, C. N.; Newkome, G. R.; Wesdemiotis, C., Design, synthesis, and traveling wave ion mobility mass spectrometry characterization of iron(II)- and ruthenium(II)-terpyridine metallomacrocycles. *J. Am. Chem. Soc.* **2011**, *133*, 11967-11976.
(6) Mesleh, M. F.; Hunter, J. M.; Shvartsburg, A. A.; Schatz, G. C.; Jarrold, M. F., Structural Information from Ion Mobility Measurements: Effects of the Long-Range Potential. *J. Phys. Chem.* **1996**, *100*, 16082-16086.
(7) Sheldrick, G., A short history of SHELX. *Acta Crystallogr. A.* **2008**, *64*, 112-122.
(8) Zhou, Y.; Kijima, T.; Izumi, T., The synthesis and application of 2-acetyl-6-(1-naphthyl)-pyridine oxime as a new ligand for palladium precatalyst in Suzuki coupling reaction. *J. Heterocycl. Chem.* **2009**, *46*, 116-118.
(9) Ono, F.; Watanabe, H.; Shinkai, S., Structural optimization of super-gelators derived from naturally-occurring mannose and their morphological diversity. *RSC Adv.* **2014**, *4*, 25940-25947.
(10) Zhang, K.-F.; Christoffel, F.; Baudoin, O., Barbier–Negishi Coupling of Secondary Alkyl Bromides with Aryl and Alkenyl Triflates and Nonaflates. *Angew. Chem., Int. Ed.* **2018**, *57*, 1982-1986.
(11) Tamura, Y.; Takezawa, H.; Fujita, M., A Double-Walled Knotted Cage for Guest-Adaptive Molecular Recognition. *J. Am. Chem. Soc.* **2020**, *142*, 5504-5508.
(12) Schultz, A.; Cao, Y.; Huang, M.; Cheng, S. Z.; Li, X.; Moorefield, C. N.; Wesdemiotis, C.; Newkome, G. R., Stable, trinuclear Zn(II)- and Cd(II)-metallocycles: TWIM-MS, photophysical properties, and nanofiber formation. *Dalton Trans.* **2012**, *41*, 11573-11575.
(13) Fu, J.-H.; Lee, Y.-H.; He, Y.-J.; Chan, Y.-T., Facile Self-Assembly of Metallo-Supramolecular Ring-in-Ring and Spiderweb Structures Using Multivalent Terpyridine Ligands. *Angew. Chem. Int. Ed.* **2015**, *54*, 6231-6235.
(14) Blanco, V.; García, M. D.; Platas-Iglesias, C.; Peinador, C.; Quintela, J. M., Dynamic formation of self-organized corner-connected square metallocycles by stoichiometric control. *Chem. Commun.* **2010**, *46*, 6672-6674.
(15) Lee, H.; Venable, R. M.; MacKerell, A. D.; Pastor, R. W., Molecular Dynamics Studies of Polyethylene Oxide and Polyethylene Glycol: Hydrodynamic Radius and Shape Anisotropy. *Biophys. J.* **2008**, *95*, 1590-1599.
(16) Perrin, C. L.; Dwyer, T. J., Application of two-dimensional NMR to kinetics of chemical exchange. *Chem. Rev.* **1990**, *90*, 935-967.
(17) Suzuki, Y.; Nakamura, T.; Iida, H.; Ousaka, N.; Yashima, E., Allosteric Regulation of Unidirectional Spring-like Motion of Double-Stranded Helicates. *J. Am. Chem. Soc.* **2016**, *138*, 4852-4859.
